# Elevated prenatal IL-6 levels disrupt early patterns of hippocampal electrical activity and chloride homeostasis by targeting GABAergic signaling at perinatal stage

**DOI:** 10.1038/s41467-026-78479-9

**Published:** 2026-10-15

**Authors:** Genni Desiato, Edoardo Fraviga, Thomas Cassani, Riccardo Grassi, Martina Vitali, Francesca Fanuele, Filippo Mirabella, Clara A. Amegandjin, Federica Ruggeri, Marco Cremonesi, David Andruszewski, Andrea Contestabile, Laura Cancedda, Riccardo Fesce, Graziella Di Cristo, Ilgiz A. Mufazalov, Marinos Kallikourdis, Michela Matteoli, Sebastiano Bariselli, Davide Pozzi

**Affiliations:** 1 Institute of Neuroscience (IN-CNR), Consiglio Nazionale delle Ricerche, 20854 Vedano al Lambro, Italy; 2 IRCCS Humanitas Research Hospital, Via Manzoni 56, 20089 Rozzano, Milan, Italy; 3 Department of Biomedical Sciences, Humanitas University, Via Rita Levi Montalcini 4, 20072 Pieve Emanuele, Milan, Italy; 4 Human Technopole, Milan, Italy; 5 Centre de Recherche Azrieli, CHU Sainte-Justine/Université de Montréal, Montréal (Québec), QC Canada; 6 Institute for Molecular Medicine, University Medical Center of the Johannes Gutenberg-University Mainz, Mainz, Germany; 7 Brain Development and Disease Laboratory, Istituto Italiano di Tecnologia, Genova, Italy

**Keywords:** Neuronal development, Synaptic development, Neuroimmunology

## Abstract

Early patterns of neuronal electrical activity are fundamental for driving critical developmental processes, including neuronal maturation and synapse formation. While genetic programs are key in orchestrating these events, the influence of environmental stressors remains poorly defined. Maternal immune activation (MIA) during pregnancy induces a systemic cytokine surge that can profoundly impact fetal brain development, increasing the risk of psychiatric and neurodevelopmental disorders. Here, we show that maternal elevation of interleukin-6 (IL-6) – a key cytokine upregulated during MIA – perturbs early hippocampal electrical activity in male offspring during the first postnatal week, a critical window for circuit formation. By triggering a STAT3-dependent modulation of early GABAergic synaptic transmission, IL-6 disrupts chloride homeostasis, causing a premature shift in the developmental GABA polarity switch. Our findings reveal a direct link between maternal immune signaling and early GABA-driven network maturation, providing insights into how inflammatory events during pregnancy may predispose male offspring to neurodevelopmental disorders.

## Abstract (web summary)

The study shows that elevated maternal IL-6 alters early hippocampal electrical activity in male offspring by disrupting GABAergic signaling and KCC2-dependent chloride homeostasis, highlighting a potential mechanism linking maternal inflammation to neurodevelopmental vulnerability.

## Introduction

During the perinatal period, the GABAergic system functions as a central hub of hyperconnected neurons^1^, providing trophic excitatory signaling^2^ and driving the emergence of primitive patterns of spontaneous electrical activity^3–5^. During that period, the elevated neuronal intracellular chloride concentration ([Cl⁻]_i_) in neurons is mostly maintained by the activity of the chloride importer Na^+^-K^+^−2Cl^−^ co-transporter 1 (NKCC1), which determines the depolarizing action of GABA signaling in the developing hippocampus and contributes to the generation of the so-called giant depolarizing potentials (GDPs)^4,6,7^. The early spontaneous electrical activity during this critical window is essential for proper circuit formation^8,9^, and is thought to represent a proto-ensemble—an early and immature precursor of neuronal assemblies—that later orchestrates a wide range of cognitive functions in adulthood^10^. As neuronal development proceeds, [Cl⁻]_i_ gradually declines due to the progressive upregulation of the chloride extruder K^+^-Cl^−^ co-transporter (KCC2), resulting in the hyperpolarizing GABAergic signaling typical of mature neural circuits, the so-called GABA polarity developmental switch process.

Maternal Immune Activation (MIA) is a well-established risk factor for neurodevelopmental disorders, as indicated by epidemiological studies showing a strong association between prenatal infections and behavioral abnormalities in offspring, including schizophrenia and autism^11,12^. Preclinical models of MIA have identified a critical role for maternal immune molecules, particularly proinflammatory cytokines^13^, soluble factors capable of crossing the placenta^14,15^, able to influence the physiological trajectory of key factors in embryonic development, including the immune^16,17^ and central nervous systems^18,19^. Among these molecules, Interleukin-6 (IL-6) has emerged as a central immune mediator during MIA, whose elevation in response to bacterial^17,20^ or viral maternal infections^18,21,22^, disrupts fetal development and is associated with behavioral abnormalities and altered brain connectivity in the offspring^23–25^. Although the precise molecular mechanisms by which maternal cytokines impact embryonic brain development remain largely unclear, most studies have focused on adult offspring, in an attempt to link behavioral abnormalities to underlying neuronal dysfunctions. Consequently, the early pathological events occurring during the perinatal period, which may set the stage for later abnormalities observed in adulthood, remain poorly understood. This gap is particularly significant in the study of neurodevelopmental disorders, which are characterized by early onset and rapid symptom progression that lead to disabling conditions later in life^26–28^. Clarifying which early biological processes in newborns are affected by maternal immune molecules is critical to uncover the pathological mechanisms responsible for neurodevelopmental disorders caused by prenatal inflammation.

Here, we investigated whether the transient maternal IL-6 elevation affects early neuronal activity in the offspring’s hippocampus. We show that prenatal exposure to IL-6 induces a biphasic alteration in hippocampal electrical activity in newborns. This begins with an early increase driven by enhanced GABAergic transmission, which leads to a premature upregulation of KCC2 expression, accelerating the developmental GABA switch, thus subsequently resulting in a reduction of neuronal activity. Moreover, this precocious KCC2 upregulation is responsible for early behavioral defects in newborns prenatally exposed to IL-6. Altogether, these data unveil a mechanism through which maternal IL-6 elevation affects the neurodevelopmental trajectory of the offspring.

## Results

### Prenatal IL-6 elevation reduces hippocampal LFP occurrence rate in newborn mice

**Fig. 1:**
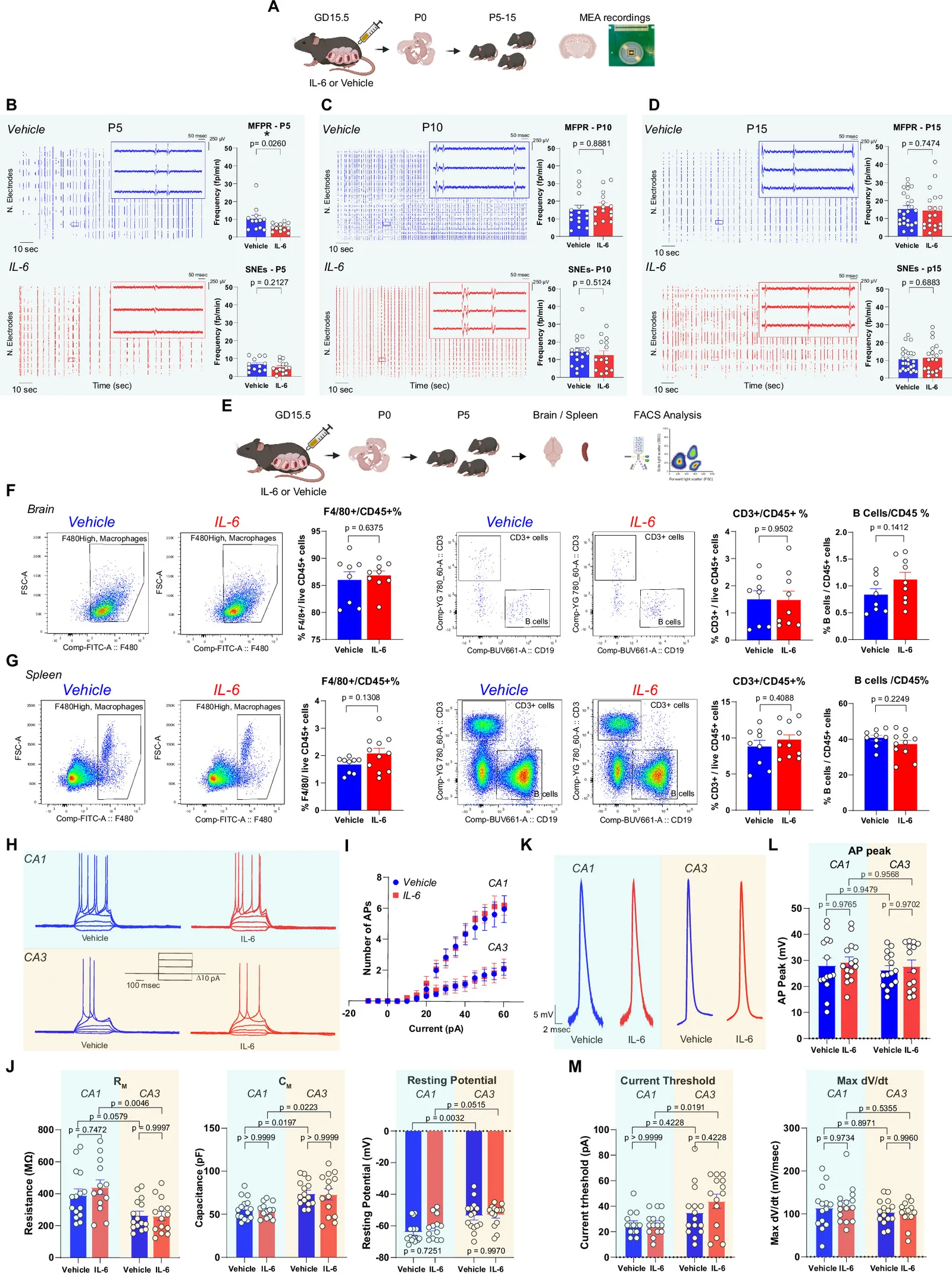
Prenatal IL-6 elevation alters the early hippocampal network activity of newborn mice. **A** Schematic cartoon showing the experimental design. **B-D** Representative raster plot with SNEs and LFPs (shown in the insets) and relative quantification in either Vehicle (top) or IL-6 (bottom) offspring at different postnatal developmental stages, P5 (**B**), P10 (**C**), P15 (**D**). Data are presented as mean ± SEM of *n* = 11 Vehicle vs *n* = 14 IL-6 slices from *N* = 5 Vehicle and *N* = 5 IL-6 P5 mice; *n* = 11 Vehicle vs *n* = 11 IL-6 slices from *N* = 3 Vehicle and *N* = 3 IL-6 P10 mice; *n* = 18 Vehicle vs *n* = 16 IL-6 slices from *N* = 3 Vehicle and *N* = 3 IL-6 P15 mice, from at least three independent experiments. Two-tailed unpaired *t*-test, *p < 0.05. **E** Schematic cartoon summarizing the experimental design. **F** Representative fluorescent-activated cell sorting (FACS) gating strategy and relative analysis of the percentage of macrophages (F4/80 + ) out of CD45+ (left panels) and the percentage of CD3+ or B cells out of CD45+ (right panels) isolated from the brain of P5 mice born to IL-6 or vehicle-injected mothers. Dots represent *N* = 8 Vehicle and *N* = 9 IL-6 -exposed mice from three independent experiments. Data are presented as mean ± SEM. Two-tailed unpaired *t*-test, ns > *p* 0.05. **G** Representative fluorescent-activated cell sorting (FACS) gating strategy and relative analysis of the percentage of macrophages (F4/80 + ) out of CD45+ (left panels) and the percentage of CD3+ or B cells out of CD45+ (right panels) isolated from the spleen of P5 mice born to IL-6 or vehicle-injected mothers. Dots represent *N* = 9 Vehicle and *N* = 11 IL-6 -exposed mice from three independent experiments. Data are presented as mean ± SEM. Two-tailed Mann-Whitney test, ns > *p* 0.05. **H** Representative traces of intrinsic neuronal excitability recorded in whole-cell current-clamp configuration from CA1 and CA3 pyramidal neurons in acute brain slices of P5 mice treated with Vehicle (blue) or IL-6 (red). Action potentials were evoked by incremental depolarizing current steps (10 pA increments). **I** Quantitative analysis of the number of action potentials in response to depolarizing current steps in CA1 and CA3 pyramidal neurons from Vehicle- and IL-6 exposed mice. **J** Quantification of passive membrane properties in Vehicle and IL-6 exposed mice from CA1 and CA3 hippocampal subfields, including Capacitance (C_M_), Membrane resistance (R_M_), and Resting potential. Data are presented as mean ± SEM of *n* = 15 vs *n* = 13 cells for CA1, *n* = 15 vs *n* = 14 for CA3, from *N* = 2 Vehicle and *N* = 2 IL-6 exposed mice, from two independent experiments. Two-way ANOVA, followed by a between-group Sidak multiple-comparison test. **K** Representative action potential waveforms recorded from CA1 and CA3 hippocampal pyramidal neurons in acute brain slices from P5 mice exposed to Vehicle (blue) or IL-6 (red). **L-M** Quantification of action potential properties in CA1 and CA3 pyramidal neurons from Vehicle and IL-6 exposed mice ; Action potential (AP) peak (**L**), current threshold, and maximum rate of membrane potential rise (maximal dV/dt) (**M**). Data are presented as mean ± SEM of n = 15 vs *n* = 13 cells for CA1, *n* = 15 vs *n* = 14 for CA3, from *N* = 2 Vehicle and *N* = 2 IL-6 exposed mice, from two independent experiments. Action potential: Two-way ANOVA, followed by between- group Sidak multiple-comparison test. Source data are provided as a Source Data file. **A** and **E**: Created in BioRender. Pozzi, D. (2026) https://BioRender.com/hoa1gtk.

The generation of early spontaneous electrical activity during postnatal development plays a crucial role in shaping neural circuit formation^29–31^, and any disruption to its physiological pattern can result in long-lasting abnormalities, including altered synaptic connectivity^32,33^ and behavioral deficits^34^ in adulthood. To investigate the early effects of maternal IL-6 elevation on this process, we took advantage of high-density multi-electrode array (HD-MEA) technology to quantitatively and dynamically monitor hippocampal electrical activity across different postnatal stages (Fig. 1A). Acute hippocampal slices from pups prenatally exposed to either vehicle (control mice) or IL-6 (IL-6 mice) at gestational day 15.5 were examined at P5, P10 and P15 through HD-MEA (Fig. 1B-D). The number of active electrodes was stable across all experimental conditions, with no differences between ages and treatments, thus reflecting a robust and reliable detection of network electrical activity (Fig. S1A). At P5, the electrical activity of the neonatal hippocampus in control mice was characterized by multiple local field potentials (LFPs) organized into synchronous network events (SNEs) (Fig. S1B, top), which propagated throughout the hippocampus and engaged neuronal populations in a TTX-sensitive manner (Fig. S1B, bottom). The frequency of LFPs, quantified as Mean Field Potential Rate (MFPR), was approximately 0.16 per second, with an average of 1,6 LFPs per SNE. This temporal pattern was consistent with the oscillatory events of GDPs previously observed in other in vitro preparations of neonatal hippocampus^5,6,35–38^ which typically engage the entire hippocampal network^39^ and are considered the in vitro counterpart of hippocampal sharp-waves (SPW) recorded in vivo^3,5,6,38,40,41^. Brain slices obtained from IL-6 mice at P5 displayed a significantly lower MFPR than control mice, accompanied by a decrease, although not statistically significant, in SNE frequency (Fig. 1B). Conversely, at P10 and P15 MFPR was comparable between control and IL-6 mice (Fig. 1C, D). The amplitude of LFPs was not altered under any of the conditions evaluated (Figure S1C). Moreover, quantification of the spike time tiling coefficient (STTC), which provides a measurement of the temporal correlation in neuronal spike times^42^, showed no alteration in either condition at any of the developmental stages analyzed (Fig. S1D, E). These data indicate that transient prenatal IL-6 exposure reduces hippocampal LFP occurrence rate at P5, without affecting network synchrony.

### The altered occurrence rate of hippocampal LFP events induced by IL-6 is not accompanied by either an early inflammatory environment or a modulation of intrinsic excitability

Previous studies have shown that prenatal immune challenges may lead to immune dysregulation in the offspring^16,17^, which may in turn affect brain activity. We have already demonstrated that prenatal IL-6 does not induce an inflamed environment within the brain parenchyma of P20 mice^19^. To assess whether immune changes occur at earlier stages of postnatal development, control and IL-6 mice were immune-profiled via multiparameter flow cytometry at P5. We examined brain tissue, as a potential site of local inflammation, and spleens, as a proxy site for systemic inflammation, evaluating both innate (F4/80^+^ macrophages, which may include microglia in the brain) and adaptive (CD3^+^ T cells and CD19^+^ B cells) immune cells (Fig. 1E–G, gating in Fig. S1F–G). We did not detect changes in the abundance of any of these cell subsets in IL-6 treated mice. Indeed, even a trend for reduction of B cells in the spleen and concomitant increase in the brain was not significant (Fig. 1F, G). Confirming this lack of significant signs of inflammation, qPCR analysis of key inflammatory markers (including cytokines, chemokines, and lineage markers that increase with inflammation^43^) in the hippocampus of pups at P5 revealed the absence of significant changes between IL-6 and control mice (Fig. S1H). Notably, the transcription factor STAT3, a key downstream molecular player in IL-6 signaling^19^, was found to be transcriptionally upregulated in the hippocampus of IL-6 mice (Fig. S1I). These data suggest that, while inducing STAT3 expression, IL-6 treatment does not produce detectable local or systemic inflammation. A reduced LFP event frequency could be the result of altered neuronal excitability^44–46^. To investigate this possibility, we assessed intrinsic excitability in CA1 and CA3 hippocampal subfields, two key regions for the generation of early electrical activity^36,39,47,48^, at P5 using whole-cell patch-clamp recordings. No differences were observed between control and IL-6 mice in the number of action potentials evoked at distinct current steps, nor in membrane resistance, capacitance or resting potential in either hippocampal subregion (Fig. 1H–J). Similarly, the kinetic properties of action potentials in CA1 and CA3 regions were comparable between the two conditions (Fig. 1K–M). Altogether, these data indicate that the reduced hippocampal LFP occurrence rate of newborns prenatally exposed to IL-6 is not attributable to an inflammatory environment and is not associated with alterations in intrinsic neuronal excitability within the hippocampus.

### IL-6 disrupts chloride homeostasis in hippocampal neurons in a STAT3 -dependent manner, leading to a premature developmental switch in GABAergic signaling

**Fig. 2:**
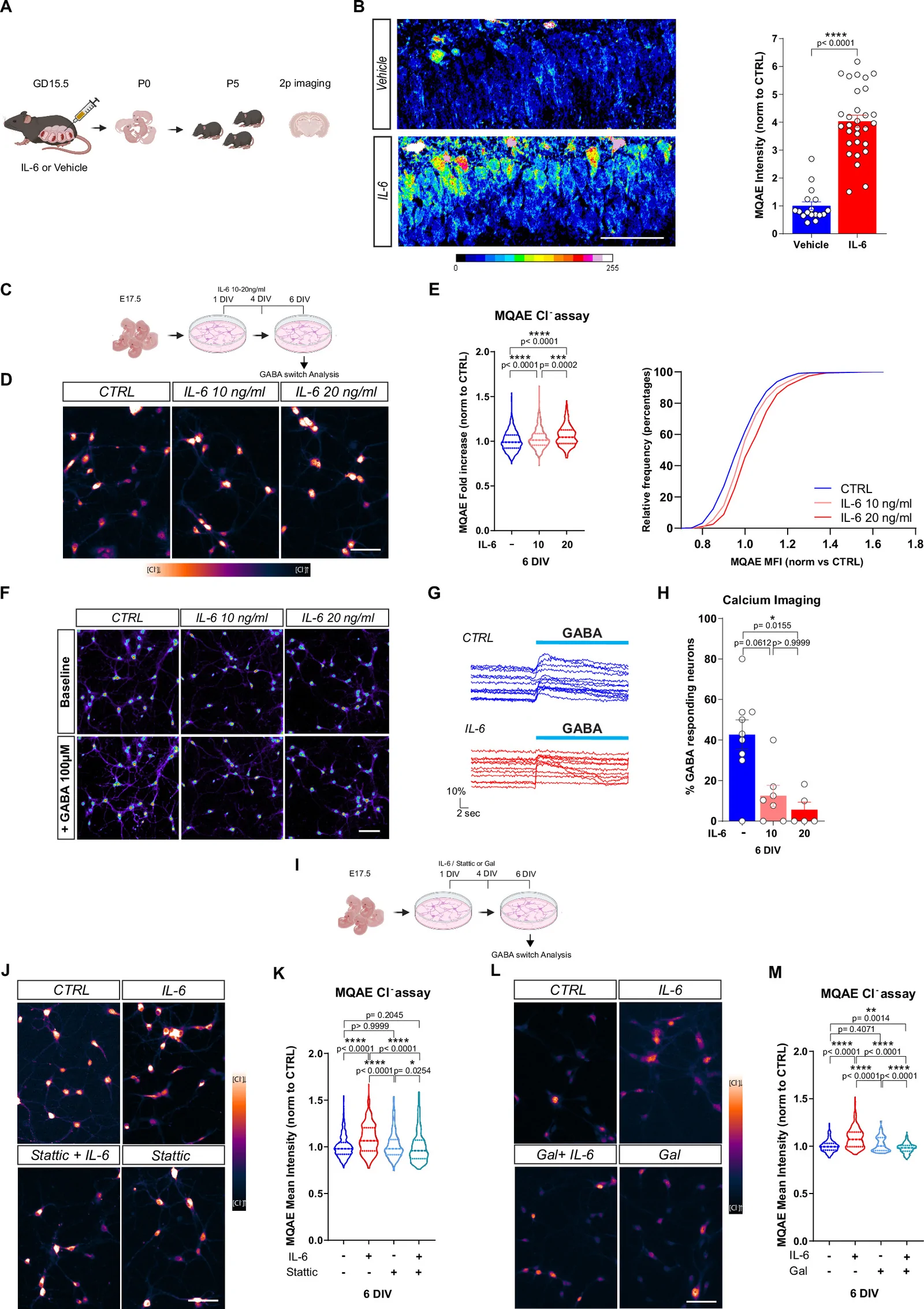
IL-6 influences the chloride homeostasis of hippocampal neurons in a STAT3 -dependent manner, boosting the GABA developmental switch. **A** Schematic cartoon showing the experimental design. **B** Representative images (left) and relative quantification (right) of Vehicle and IL-6 exposed acute hippocampal slices loaded with MQAE and imaged with multiphoton microscopy showing a higher relative fluorescence intensity in IL-6 vs Vehicle counterpart. Data are presented as mean ± SEM of *n* = 17 vs *n* = 30 cells from *N* = 3 Vehicle and *N* = 3 IL-6 exposed mice from three independent experiments. Two-tailed unpaired *t*-test, ****p < 0.001. **C** Schematic cartoon showing the experimental design for in vitro culture preparation and analysis. **D** Representative images showing MQAE chloride imaging recordings on 6 DIV hippocampal neurons administered with increasing concentrations of IL-6. Magnification 20X. Scale bar 50 µm. **E** Quantification (left) and related cumulative probability graph (right) indicating MQAE relative fluorescence increase in hippocampal neurons administered with increasing doses of IL-6. Violin plots showing mean ± SEM of *n* = 579 CTRL vs *n* = 551 IL-6 10 vs *n* = 399 IL-6 20 cells from *N* = 2 independent experiments. Kruskal-Wallis one-way ANOVA, ***p < 0.001, ****p < 0.0001. **F** Representative images showing GABA-induced calcium transients in 6 DIV hippocampal neurons treated with increasing concentrations of IL-6. Magnification 20X. Scale bar 100 µm. **G** Representative traces of calcium imaging recordings from Ctrl or IL-6 treated neurons. **H** Histograms showing a reduction of the percentage of GABA-responding neurons with increasing doses of IL-6. Data are presented as mean ± SEM of *n* = 8 ctrl vs *n* = 7 IL-6 10 ng/ml vs *n* = 5 IL-6 20 ng/ml fields of recording from *N* = 3 independent experiments. One-way ANOVA, *p < 0.05. **I** Schematic cartoon showing the experimental design for in vitro culture preparation and analysis. **J** Representative images showing MQAE chloride imaging recordings in hippocampal neurons treated with IL-6 or STAT3- cytoplasmic blocker Stattic. Magnification 20X. Scale bar 50 µm. **K** Violin plots showing the quantification of  relative MQAE fluorescence in hippocampal neurons demonstrating that the IL-6-dependent increase in MQAE fluorescence is prevented by Stattic administration. Data are represented as mean ± SEM of *n* = 591 CTRL vs *n* = 490 IL-6 vs *n* = 555 Stattic vs *n *= 400 Stattic+IL-6 cells from *N* = 3 independent experiments. Kruskal–Wallis One Way ANOVA, ****p < 0.0001. **L** Representative images showing MQAE chloride imaging recordings in 6 DIV hippocampal neurons treated with IL-6 or STAT3- genomic blocker Galiellalactone (Gal). Magnification 20X. Scale bar 50 µm. **M** Violin plots showing the quantification of MQAE relative fluorescence in hippocampal neurons demonstrating that the IL-6-dependent MQAE fluorescence increase is prevented by Galiellalactone administration. Data are represented as  mean ± SEM of *n* = 343 CTRL vs *n* = 404 IL-6 vs *n* = 130 Gal vs *n* = 453 Gal+IL-6 cells from *N* = 3 independent experiments. Kruskal–Wallis one-way ANOVA, **p < 0.001, ****p < 0.0001. Source data are provided as a Source Data file. **A**, **C**, **I**: Created in BioRender. Pozzi, D. (2026) https://BioRender.com/hoa1gtk.

In the neonatal hippocampus, spontaneous neuronal activity is largely sustained by the early depolarizing action of GABAergic signaling^3^, which relies on the elevated intracellular chloride concentration [Cl^-^]_i_, that progressively declines during postnatal development. Consistently, two-photon imaging using the chloride-sensitive dye MQAE, whose fluorescence is inversely proportional to [Cl-]_i_, revealed that chloride levels in CA1 neurons at P5 were significantly higher than those at P15 (Fig. S2A), with a lower decay time measured via Fluorescence Lifetime Intensity Measurement (FLIM) (Fig. S2B). Furthermore, the pharmacological reduction of [Cl-]_i_ through the loop diuretic bumetanide, a selective NKCC1 inhibitor, clearly reduced the MFPR at P5 (Fig. S2C, top) but not at P15 (Fig. S2C, bottom). These findings indicate that [Cl^-^]_i_ plays a crucial role in setting hippocampal electrical activity at early developmental stages^34,49–51^ and prompted us to explore the potential impact of prenatal IL-6 on [Cl^-^]_i_ in neonatal hippocampal neurons. Acute brain slices obtained from P5 control and IL-6 mice were loaded with MQAE and CA1 hippocampal neurons were imaged via two-photon microscopy (Fig. 2A). We found that neurons from IL-6- exposed mice displayed higher MQAE fluorescence than controls, reflecting a significantly lower [Cl^-^]_i_ (Fig. 2B), indicating that prenatal IL-6 elevation modulates hippocampal chloride homeostasis in neurons at early postnatal stages. To better dissect this phenomenon, we took advantage of cultured hippocampal neurons^19^ (Fig. S2D), which also undergo a dynamic regulation of chloride homeostasis during development, as demonstrated by i) the progressive increase of MQAE fluorescence, reflecting the lowering of [Cl-]_i_ (Fig. S2E–F); ii) the sharp upregulation of the KCC2 transcript expression (*Slc12a5*) paralleled by a downregulation of the chloride importer NKCC1 (*Slc12a2*) (Fig. S2G); iii) the percentage of neurons responding with calcium transients to GABA during development, which is the highest at early stages (Fig. S2H) (4 Days In Vitro, DIV, high [Cl^-^]_i_, 91.2% ± 3.171) and progressively decreases over time (Fig. S2I), revealing a significant correlation between [Cl⁻]ᵢ and GABA responsiveness at the various developmental time points (Fig. S2J). Hippocampal cultured neurons transiently treated at early stages of development (1–4 DIV^19^) with exogenous IL-6 (10 -20 ng/ml) and then imaged at 6 DIV (Fig. 2C) displayed a dose-dependent enhancement of MQAE fluorescence (Fig. 2D-E), indicating that IL-6 can prematurely lower [Cl-]_i_. Accordingly, calcium imaging recordings showed that the percentage of GABA-responsive neurons was reduced upon IL-6 treatment (Fig. 2F-H). Of note, a transient application of IL-6 for 2 h at 1 DIV was sufficient to produce a significant increase in MQAE fluorescence in neurons at 6 DIV (Fig. S2K), whereas no effect on chloride homeostasis was observed if IL-6 was acutely applied for 2 hours at 6 DIV (Fig. S2L), suggesting that a neuronal transcriptional modification is required. In line with the role played by the transcription factor STAT3 in IL-6-dependent signaling, blocking either the phosphorylation or the DNA-binding activity of STAT3 via the small molecules Stattic and Galiellalactone^19^ (Fig. 2I), respectively, occluded the IL-6-mediated effect on chloride homeostasis (Fig. 2J-M). Altogether, these data indicate that IL-6 produces a premature lowering of [Cl-]_i_ in neurons and depresses GABA depolarizing responses, via the genomic effect of STAT3.

### KCC2 expression is prematurely increased in neurons prenatally exposed to IL-6 and its inhibition affects both circuit activity and behavioral outcomes

**Fig. 3:**
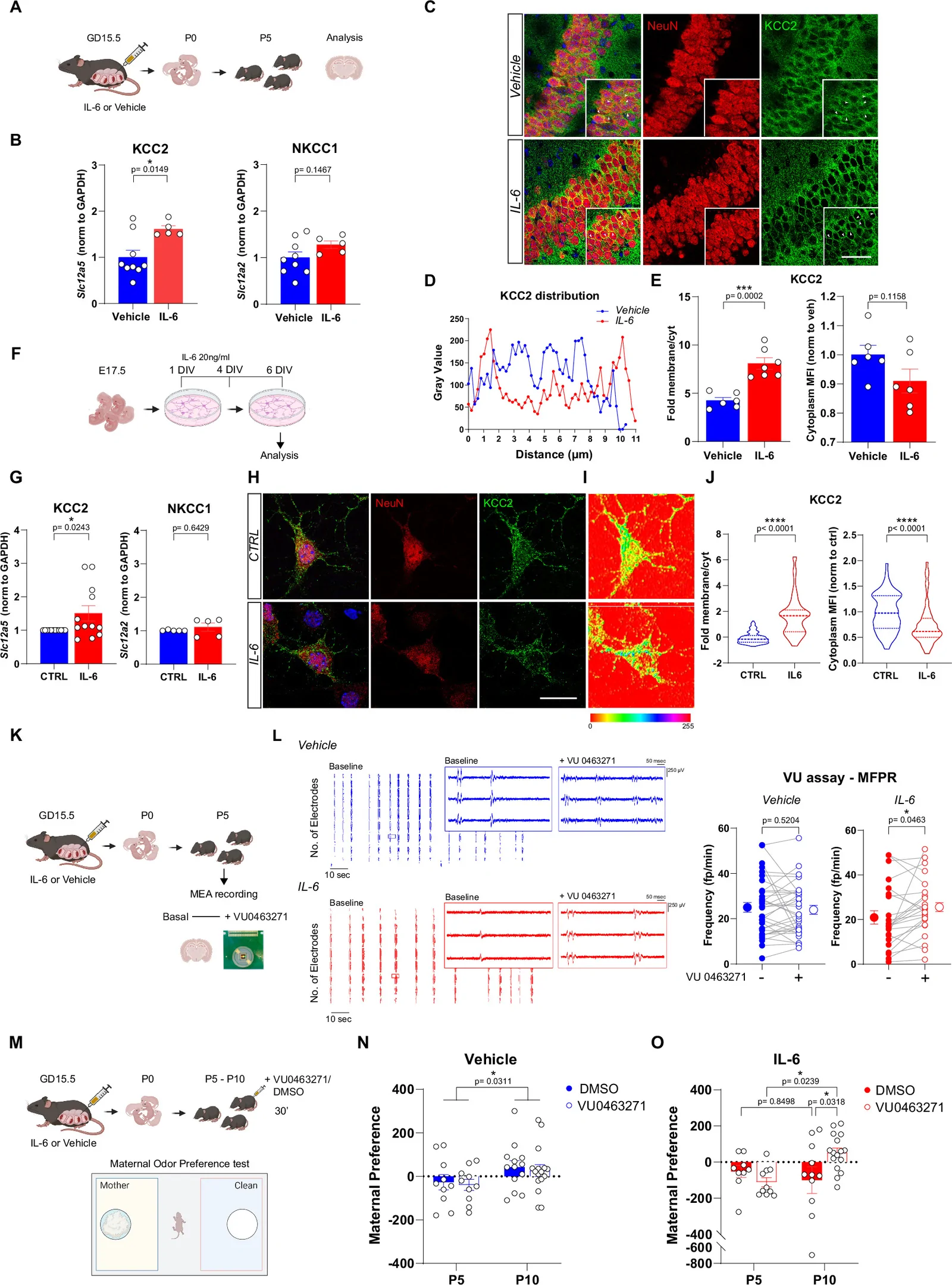
KCC2 expression is prematurely enhanced in neurons prenatally exposed to IL-6. **A** Schematic cartoon showing the experimental design. **B** Quantification of the relative mRNA expression of chloride transporters KCC2 *(Slc12a5)* and NKCC1 (*Slc12a2)* in the hippocampi of Vehicle and IL-6 exposed mice. Dots represent *N* = 9 Vehicle vs *N* = 5 IL-6 exposed mice from at least three independent experiments. Data are presented as mean ± SEM. Two-tailed unpaired *t*-test. *p < 0.05. **C** Representative confocal images of CA1 hippocampal pyramidal neurons from Vehicle or IL-6 exposed mice immunostained against neuronal marker NeuN (red) and chloride transporter extruder KCC2 (green). Nuclei counterstained with Hoechst (blue). Magnification 40X. Scale bar 50 µm. **D** Representative line-scan profiles of KCC2 fluorescence intensity in  Vehicle and IL-6 conditions. **E** Quantification of  KCC2 immunoreactivity showing the fold increase in the membrane-to-cytoplasmic immunoreactivity ratio (left) and baseline cytoplasmic KCC2 mean fluorescence intensity (MFI) normalized to the control value (right). Dots represent *N* = 6 Vehicle vs *N* = 7 IL-6 mice from at least three independent experiments. Data are presented as mean ± SEM. Two-tailed unpaired *t*-test, ***p < 0.001. **F** Schematic cartoon showing the experimental in vitro design. **G** Quantification of the relative mRNA expression of chloride transporters KCC2, *Slc12a5* (left) and NKCC1, *Slc12a2* (right) in CTRL and IL-6-treated hippocampal neurons. Data are presented as mean ± SEM of *N* = 12 (*Slc12a5*) and *N* = 5 (*Slc12a2*) independent experiments. Two-tailed unpaired *t*-test, *p < 0.05. **H** Representative confocal images of 6 DIV hippocampal neurons, with or without IL-6 administration, immunostained against neuronal marker NeuN (red) and chloride transporter extruder KCC2 (green). Nuclei counterstained with Hoechst (blue). Magnification 40X. Scale bar 10 µm. **I** Representative images showing KCC2 intensity surface plot comparison between CTRL and IL-6 treatment. **J** Violin plots showing the membrane-to-cytoplasmic KCC2 immunoreactivity ratio (left) and baseline cytoplasmic KCC2 mean fluorescence intensity (MFI) normalized to control values (right). Data are presented as mean ± SEM of *n* = 102 CTRL vs n = 94 IL-6-treated cells from *N* = 3 independent experiments. Data are presented as mean ± SEM. Two-tailed unpaired t-test, ****p < 0.001. **K** Schematic cartoon representing the in vivo experimental design. **L** Representative raster plots with SNEs and LFPs (shown in the insets) from either Vehicle or IL-6 exposed P5 mice before and after VU0463271 (VU) administration (left) and relative MFPR quantification (right). Data are presented as a pre-post graph of *n* = 34 Vehicle vs *n* = 24 IL-6 slices from *N* = 5 Vehicle and *N* = 6 IL-6 exposed mice from at least three independent experiments. Two-tailed paired *t*-test, *p < 0.05. **M** Schematic cartoon representing the behavioral test design. **N-O** Maternal Odor Preference Test. Graphs showing the maternal preference quantified as time spent in the “mother” zone minus the combined time spent in the “clean” and “neutral” zones in Vehicle or IL-6 exposed P5 vs P10 mice treated with VU0463271 30 minutes before the test. Dots represent: for P5 mice, *N* = 10 Vehicle + DMSO vs *N* = 10 Vehicle + VU, *N* = 9 IL-6 + DMSO vs *N* = 10 IL-6 + VU; for P10 mice, *N* = 13 Vehicle + DMSO vs *N* = 16 Vehicle + VU, *N* = 10 IL-6 + DMSO vs *N* = 16 IL-6 + VU from at least three independent experiments. Vehicle: Two-Way ANOVA (main effect of treatment: *p* = 0.6555; main effect of age: *p* = 0.0311; treatment x age interaction: *p* = 0.9598) followed by between- group Sidak multiple comparison test, ns, *p* > 0.05. IL-6: Two-Way ANOVA (main effect of treatment: *p* = 0.2946; main effect of age: *p* = 0.1960; treatment x age interaction: *p* = 0.0207) followed by between-group Šidák’s multiple comparison test, *p < 0.05. Source data are provided as a Source Data file. **A**, **F**, **K**, **M**: Created in BioRender. Pozzi, D. (2026) https://BioRender.com/hoa1gtk.

We next evaluated the transcriptional profile of the two chloride transporters involved in the regulation of chloride homeostasis, NKCC1 and KCC2. Quantitative PCR analysis (qPCR) performed in hippocampi of P5 control and IL-6 mice (Fig. 3A) revealed a significant upregulation of KCC2 transcript *Slc12a5* in mice exposed to IL-6, whilst NKCC1 levels (*Slc12a2*) remained unchanged (Fig. 3B). Furthermore, the membrane localization of KCC2, assessed by immunofluorescence analysis, was higher in CA1 pyramidal neurons of IL-6 mice compared to controls (Fig. 3C–E). Similar results were also obtained in hippocampal neuronal cultures exposed to IL-6 (Fig. 3F), which displayed upregulation of KCC2, but not NKCC1, transcription (Fig. 3G) and enhanced membrane localization of KCC2 compared to control cultures (Fig. 3H–J). To note, the transcriptional KCC2 levels remained significantly elevated even at P10, indicating a stable upregulation of this chloride transporter over time (Fig. S3A).

These results indicate that prenatal IL-6 elevation leads to selective upregulation of KCC2 in hippocampal neurons, resulting in premature lowering of neuronal [Cl⁻]_i_. To assess whether the early KCC2 upregulation is directly linked to the altered hippocampal LFP event frequency recorded in IL-6 mice, KCC2 activity was pharmacologically inhibited through the specific compound VU0463271. Recording of electrical activity in hippocampal slices via HD-MEA in P5 mice (Fig. 3K), revealed that although the application of the KCC2 inhibitor increases the frequency of SNEs in both conditions (Fig. S3B), the MFPR was significantly increased only in acute slices from IL-6 but not control mice (Fig. 3L). These data confirm that KCC2 activity is prematurely increased in the hippocampus of mice prenatally exposed to IL-6 and that its blockade impacts LFP occurrence rate exclusively in IL-6 mice.

We then investigated whether the upregulation of KCC2 activity in response to prenatal IL-6 elevation correlated with observable behavioral impairments at this early postnatal stage. To this aim we assessed the function of the “attachment system” using the maternal odor preference (MOP) test (Fig. 3M)^52,53^. Control and IL-6 mice were video-recorded and the cumulative time spent on the MO (maternal odor)-paired, clean litter (CL)-paired side, or neutral central zone of a clean cage was automatically measured during five trials of 1 min each^54^. To determine whether the hippocampus is involved in MOP, we quantified c-Fos expression in CA1 following the behavioral test, comparing animals that performed the task with animals that did not (Fig. S3C,D). We observed a striking increase in c-Fos–positive cells in the CA1 region of animals exposed to MOP, demonstrating that the hippocampus is a key brain region recruited during the MOP task (Fig. S3E). Based on this evidence, MOP was assessed at two developmental time points, P5 and P10. As during this developmental time window the hippocampus of IL-6 mice was characterized by precocious KCC2 upregulation, we investigated whether the pharmacological inhibition of KCC2 might modify MOP. Control and IL-6 mice were injected with 100 mg/kg VU0463271 30 minutes before the MOP test at both time points. We found that in control animals treated with either DMSO or VU0463271, stimulus discrimination during MOP improved over time with animals showing reduced time spent in the neutral zone (Fig. S3F–G) and increased preference for the maternal side (Fig. 3N). In contrast, IL-6–exposed animals treated with DMSO did not show age-dependent stimulus discrimination (Fig. S3H), resulting in blunted maternal odor preference at P10 (Fig. 3O). Notably, VU0463271 treatment rescued exploratory dynamics (Fig. S3I) and restored maternal odor preference in IL-6 animals (Fig. 3O). To test whether other behavioral phenotypes were compromised in IL-6 mice, ultrasonic vocalizations (USVs) were also evaluated at the same developmental stages (Fig. S3J, L). While no differences were observed in either the number or duration of calls at P5 (Fig. S3K), at P10 IL-6-exposed mice exhibited a reduction in call number compared to controls, which was fully rescued by VU0463271 treatment (Fig. S3M, left), without affecting the duration of the calls (Fig. S3M, right). Altogether, these findings indicate that premature KCC2 upregulation triggered by prenatal IL-6 elevation disrupts developmental behavioral trajectories, and these impairments could be rescued by pharmacological inhibition of KCC2 in vivo.

### Prenatal IL-6 elevation enhances early GABAergic transmission by directly activating STAT3 signaling in GABAergic neurons

**Fig. 4:**
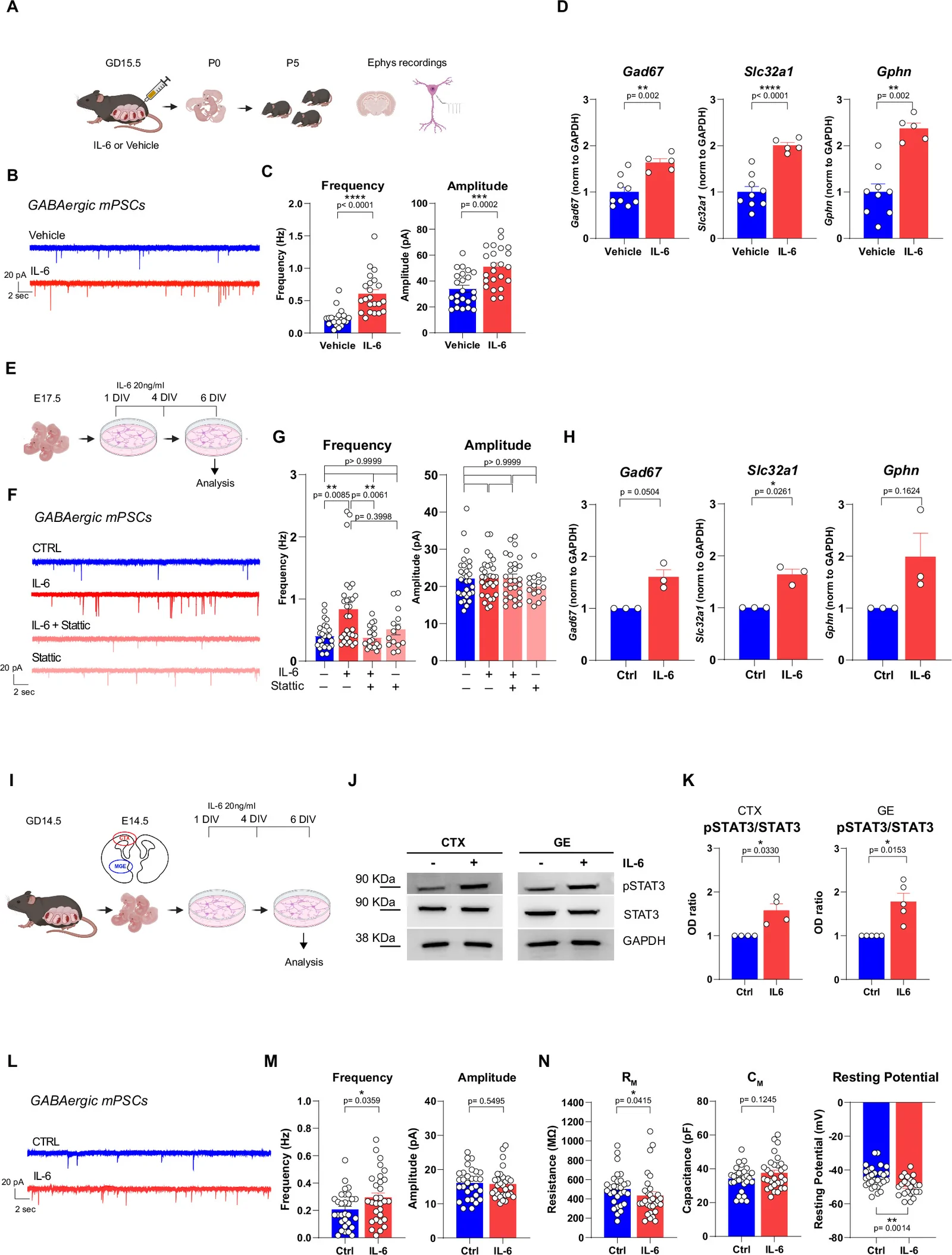
Prenatal IL-6 elevation enhanced the early GABAergic transmission by directly engaging STAT3 activity in GABAergic neurons. **A** Schematic cartoon showing the experimental design. **B** Representative traces of GABAergic mPSC recordings in the CA1 region of hippocampal slices from Vehicle or IL-6 exposed P5 mice. Scale bar 20 pA, 2 Sec. **C** Histograms showing GABAergic mPSC frequency (left) and Amplitude (right) of *n *= 23 vs *n* = 24 cells from *N* = 4 Vehicle and *N* = 4 IL-6 from four independent experiments. Data are presented as mean ± SEM. Two-tailed Unpaired *t* test, ***p < 0.001, ***p < 0.0001. **D** Quantification of relative mRNA expression of GABA-related genes, Glutamate decarboxylase *Gad67* (left), vesicular GABA transporter *Slc32a1* (middle) and gephyrin *Gphn* (right) in the hippocampi of Vehicle and IL-6 exposed mice. Dots represent *N* = 9 Vehicle vs *N* = 5 IL-6 exposed mice from at least three independent experiments. Data are presented as mean ± SEM. Two-tailed Unpaired *t* test, **p < 0.01; ***p < 0.001, ***p < 0.0001. **E** Schematic cartoon showing the experimental design. **F** Representative traces of GABAergic mPSC recordings from hippocampal neurons with different treatment conditions. Scale bar 20 pA, 2 Sec. **G** Histograms showing GABAergic mPSC frequency (left) and Amplitude (right) of *n* = 24 CTRL vs *n* = 30 IL-6 vs *n* = 19 IL-6+ Stattic vs *n* = 14 Stattic -treated cells from *N* = 3 independent experiments. Data are presented as mean ± SEM. One-way ANOVA, **p < 0.01. **H** Quantification of mRNA relative expression of GABA-related genes, Glutamate decarboxylase *Gad67* (left), vesicular GABA transporter *Slc32a1* (middle), and gephyrin *Gphn* (right) in CTRL and IL-6-treated hippocampal neurons. Data are presented as mean ± SEM of *N* = 3 independent experiments. Two-tailed unpaired *t*-test, *p < 0.05. **I** Schematic cartoon showing the experimental in vitro design. **J** Representative images of western blotting protein analysis in CTX and GE neurons with or without IL-6 administration. **K** Quantification of the pSTAT3/STAT3 protein ratio  in CTX (left) and GE (right). Data are presented as mean ± SEM of *N* = 4 CTX and *N* = 5 GE independent experiments. Two-tailed unpaired *t*-test, *p* < 0.05. **L** Representative traces of GABAergic mPSC recordings from GE neurons with different treatment conditions. Scale bar 20 pA, 2 Sec. **M** Histograms showing GABAergic mPSC frequency (left) and Amplitude (right) of *n* = 30 CTRL vs *n* = 30 IL-6 -treated cells from *N* = 3 independent experiments. Data are presented as mean ± SEM. Two-tailed unpaired *t*-test, *p < 0.05. **N** Quantification of electric passive properties in GE neurons with or without IL-6 administration; Membrane resistance (R_M_), Membrane capacitance (C_M_) and Resting potential. Data are presented as mean ± SEM of *n* = 30 vs *n* = 30 cells from *N* = 3 independent experiments. Two-tailed Unpaired *t* test, *p < 0.05, **p < 0.01. Source data are provided as a Source Data file. **A**, **E**, **I** Created in BioRender. Pozzi, D. (2026) https://BioRender.com/hoa1gtk.

KCC2 upregulation and the developmental GABA polarity switch are thought to be also driven by GABA signaling itself^55,56^, implying that this form of synaptic activity must emerge early during development. To test this hypothesis, we performed a time-course analysis of pharmacologically isolated Glutamatergic and GABAergic basal synaptic transmission in the neonatal hippocampus across postnatal development. Miniature postsynaptic currents (mPSCs) were recorded via patch-clamp in CA1 pyramidal hippocampal neurons in brain slices established from pups at P3, P6 and P9 (Fig. S4A). During this period, neurons underwent a progressive maturation of their passive properties, as indicated by a steady increase in membrane capacitance accompanied by a progressive reduction of membrane resistance and resting potential (Fig. S4B). At P3 GABAergic mPSCs represented the predominant form of synaptic activity, with 80% of recorded neurons exhibiting detectable GABAergic transmission (Fig. S4C, left, S4D), whereas glutamatergic transmission became detectable at P6 in 50% of the recorded neurons (Fig. S4C, middle, S4D). At later developmental stages (P9), GABAergic mPSCs frequency underwent a sharp increase, whereas glutamatergic transmission exhibited a slower and less pronounced increase (Fig. S4C, right, S4D). Consistent with the predominant contribution of GABAergic synaptic activity at early stages, spontaneous synaptic events recorded in pyramidal neurons were strongly inhibited by bath application of the GABA-A receptor blocker bicuculline at both P3 and P6 (Fig. S4E–F)﻿. By P9, a residual synaptic activity, attributable to functional glutamatergic synapses, became detectable (Fig. S4E–F). Together, these findings indicate that GABAergic activity is the first functional synaptic activity to emerge during hippocampal development, consistent with previous studies performed in similar experimental models^57,58^. We then asked whether prenatal IL-6 elevation interferes with this early synaptic transmission (Fig. 4A). We found that both frequency and amplitude of GABAergic mPSCs were significantly enhanced in CA1 hippocampal neurons at P5 in IL-6 mice compared to controls (Fig. 4B-C), indicating that IL-6 modulates early GABAergic synaptic activity. In addition, a significant increase in transcripts encoding the GABA-related genes, *Gad67*, *Slc32a1* and *Gphn* was detected in hippocampi at the same age, prenatally exposed to IL-6 (Fig. 4D). To further investigate this phenomenon, we utilized primary cultures of hippocampal neurons where GABAergic transmission also represents the major form of synaptic activity during early developmental stages (Fig. S4G-J). IL-6 was applied at 1 and 4 DIV, before the appearance of synaptic activity, and the GABAergic mPSCs were measured at 6 DIV (Fig. 4E). In line with the effects observed following prenatal IL-6 elevation in ex vivo preparation, IL-6 enhanced the basal GABAergic transmission in hippocampal cultured neurons (Fig. 4F-G), suggesting a direct role on neurons, and upregulated the two GABA-related genes *Gad67* and *Slc32a1* (Fig. 4H). Again, the IL-6-mediated effect on GABAergic transmission was prevented by the STAT3 inhibitor Stattic (Fig. 4F-G). Since primary hippocampal cultures are composed of a mixed population of glutamatergic and GABAergic neurons, to unequivocally prove a direct role of IL-6 on GABAergic cells, we leveraged neuronal cultures established from the ganglionic eminences (GE)^59,60^, an embryonic brain region involved in the generation of GABAergic neurons and devoid of excitatory neurons^61^. As a control condition we used cortical neurons established from the cortex at the same embryonic stage (Fig. 4I). GE-derived neuronal cultures established at embryonic day 14.5 (E14.5) were highly enriched for GABAergic neurons, as indicated by the predominant expression of GABA-related genes (*Gad67*, *Dlx*, and *Slc32a1*), the relatively low expression levels of glutamatergic-related genes (*Slc17a7*) (Fig. S4K) and the high prevalence of GAD67-positive neurons in GE cultures as compared to cortical cultures (Fig. S4L). Functionally, GE-derived cultures displayed a higher frequency of GABAergic than Glutamatergic mPSCs, reflecting a lower Glutamate-to-GABA (Glut/GABA) ratio (Fig. S4M). GE cultures treated with IL-6 displayed a strong increase of STAT3 phosphorylation levels (Fig. 4J, K), reflecting the activation of the transcription factor^19,62–65^ accompanied by a significant enhancement of GABAergic mPSCs (Fig. 4L-N). Overall, these data indicate that IL-6 can directly target GABAergic neurons enhancing their synaptic activity in a STAT3-dependent manner.

### IL-6-driven GABAergic transmission enhancement precedes and drives the accelerated GABA developmental switch

**Fig. 5:**
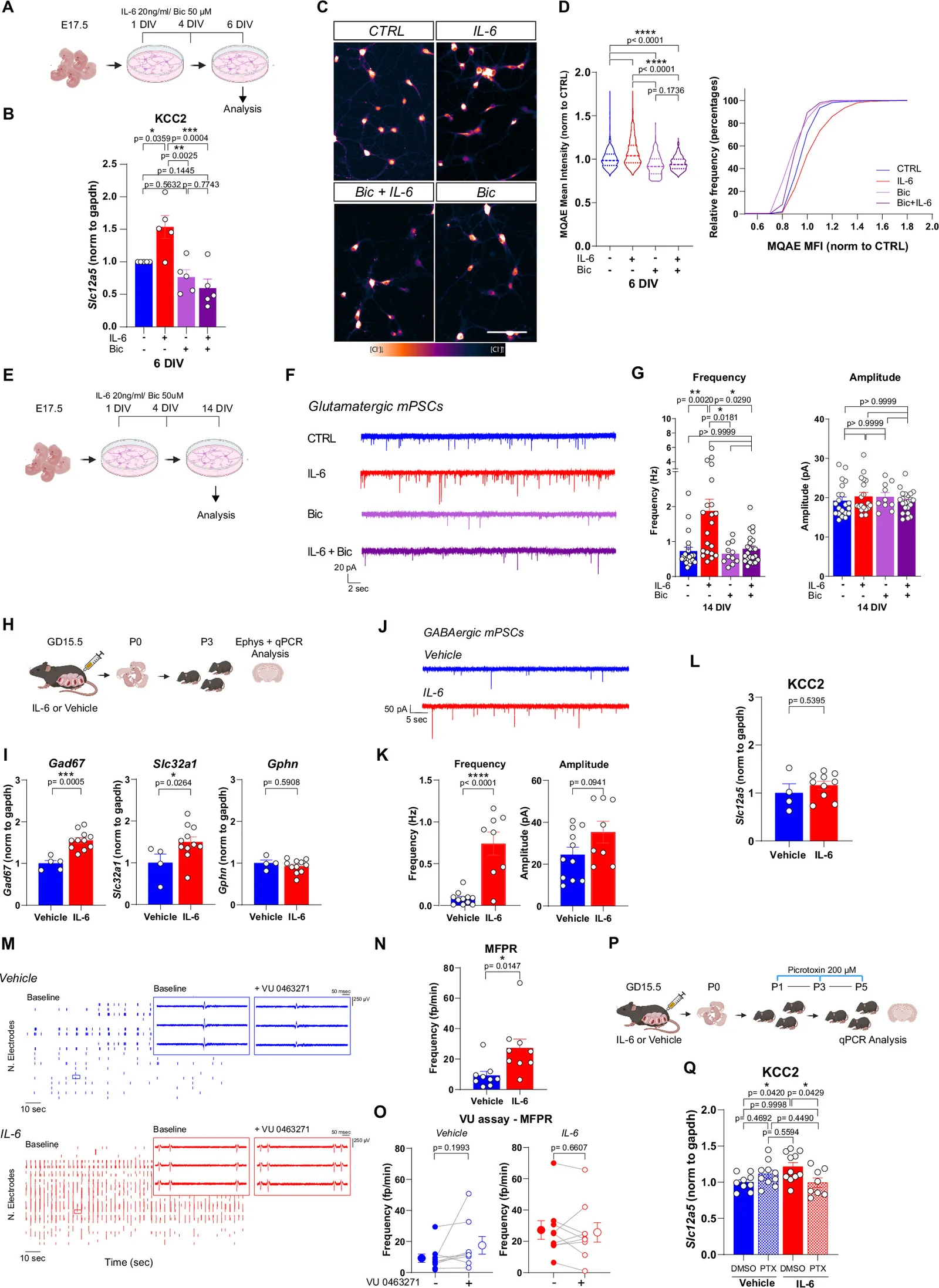
The IL-6-driven GABAergic transmission enhancement precedes and drives the accelerated GABA developmental switch. **A** Schematic cartoon showing the experimental in vitro design. **B** Quantification of relative mRNA expression of chloride transporter KCC2 *(Slc12a5)* in hippocampal neurons administered with IL-6 or GABA_A_ receptor antagonist Bicuculline (Bic). Data are presented as mean ± SEM of *N* = 5 independent experiments. One-way ANOVA, followed by Tukey correction, *p < 0.05, **p < 0.01, ***p < 0.001. **C** Representative images showing MQAE chloride imaging recordings in hippocampal neurons treated with IL-6 or GABA_A_ receptor antagonist Bicuculline (Bic). Magnification 20X. Scale bar 50 µm. **D** Violin plots  (left) and corresponding cumulative distribution plot (right) of relative MQAE fluorescence quantification in hippocampal neurons showing that Bicuculline treatment prevents the IL-6-dependent increase in MQAE fluorescence. Data are presented as mean ± SEM of *n* = 684 CTRL vs *n* = 542 IL-6 vs *n* = 261 Bic vs *n* = 406 Bic+IL-6 cells from *N* = 4 independent experiments. One-way ANOVA, followed by Tukey correction, ****p < 0.0001. **E** Schematic cartoon showing the experimental in vitro design. **F** Representative traces of glutamatergic mPSC recordings from hippocampal neurons with different treatment conditions. Scale bar 20 pA, 2 Sec. **G** Histograms showing glutamatergic mPSC frequency (left) and Amplitude (right) of *n* = 21 CTRL vs *n* = 21 IL-6 vs *n* = 11 Bic vs *n* = 23 Bic + IL-6 -treated cells from *N* = 3 independent experiments. Data are presented as mean ± SEM. One-way ANOVA, followed by Tukey correction, *p < 0.05, **p < 0.01. **H** Schematic cartoon showing the experimental design. **I** Quantification of relative mRNA expression of (from left to right), Glutamate decarboxylase *Gad67*, vesicular GABA transporter *Slc32a1* and gephyrin *Gphn* in the hippocampi of Vehicle and IL-6 exposed P3 mice. Data are presented as mean ± SEM of *N* = 4 Vehicle *N* = 11 IL-6-exposed mice from at least three independent experiments. Two-tailed Mann-Whitney unpaired t-test, *p < 0.05, ***p < 0.001. **J** Representative traces of GABAergic mPSCs recordings in the CA1 region of hippocampal slices from Vehicle or IL-6 exposed P3 mice. Scale bar 20 pA, 2 Sec. **K** Histograms showing GABAergic mPSC frequency (left) and Amplitude (right) of *n* = 11 vs *n* = 8 cells from *N* = 3 Vehicle and *N* = 3 IL-6-exposed P3 mice, from three independent experiments. Data are presented as mean ± SEM. Two-tailed unpaired t-test, ****p < 0.0001. **L** Quantification of relative mRNA expression of chloride transporter KCC2 *Slc12a5*, in the hippocampi of Vehicle and IL-6 exposed P3 mice. Data are presented as mean ± SEM of *N* = 4 Vehicle *N* = 10 IL-6-exposed mice from at least three independent experiments. Two-tailed unpaired *t*-test, ns *p* < 0.05. **M **Representative raster plots with SNEs and LPFs (shown in the insets) from P3 mice exposed to either Vehicle or IL-6  with the corresponding quantification of the MFPR **(N)**. **O **Quantification of relative MFPR before and after acute VU0463271 administration. Data are presented as pre-post graphs of *n* = 8 Vehicle vs *n* = 8 IL-6 slices from *N* = 3 Vehicle vs *N* = 3 IL-6 exposed mice from three independent experiments. Two-tailed paired *t*-test, *p < 0.05. **P** Schematic cartoon showing the experimental design. **Q** Quantification of relative mRNA expression of chloride transporter KCC2 *Slc12a5*, in the hippocampi of Vehicle and IL-6 exposed P5 mice with or without Picrotoxin (PTX) i.p. administration (200 μM, 10 μl). Data are presented as mean ± SEM of *N* = 9 Vehicle + DSMO, *N* = 10 Vehicle + Ptx, *N* = 11 IL-6 + DSMO, *N* = 8 IL-6 + Ptx-exposed mice from at least three independent experiments. One Way ANOVA, ns *p* < 0.05. Source data are provided as a Source Data file. **A**, **E**, **H**, **P**: Created in BioRender. Pozzi, D. (2026) https://BioRender.com/hoa1gtk.

We then reasoned that the early enhancement of GABAergic transmission in response to prenatal IL-6 elevation might be instrumental in producing the premature transcriptional upregulation of KCC2 and the consequent altered chloride homeostasis. We thus tested whether GABA signaling itself affects the GABA developmental switch. Hippocampal cultures treated at early stages with either GABA or bicuculline (Fig. S5A) exhibited decreased or increased neuronal [Cl⁻]ᵢ, respectively, as indicated by MQAE fluorescence measured at 6 DIV (Fig. S5B). This was paralleled by lower or higher percentages, respectively, of GABA-responding neurons as assessed by calcium imaging recordings (Fig. S5C). These results confirm that GABA signaling is a key regulator of the GABA polarity developmental switch^55,56^ and prompted us to investigate whether IL-6-mediated GABAergic potentiation drives this process. To test this hypothesis, hippocampal cultures were treated with IL-6 at early stages in either the presence or the absence of Bicuculline, and assessed for KCC2 expression an [Cl⁻]ᵢ levels (Fig. 5A). The transcriptional upregulation of KCC2 induced by IL-6 did not occur in the presence of bicuculline (Fig. 5B). Accordingly, blocking GABAergic transmission by bicuculline prevented the IL-6-induced MQAE fluorescence increase (Fig. 5C-D), providing a causal link between the synaptic GABAergic potentiation induced by IL-6 and the reduction of neuronal [Cl⁻]ᵢ. Notably, the early blockade of GABAergic transmission (Fig. 5E) also prevented the IL-6-dependent increase of the frequency of Glutamatergic synaptic transmission at mature stages (Fig. 5F-G)^19^, thus suggesting a causal relationship between the two phenomena. To exclude that IL-6-induced KCC2 upregulation might be responsible for the observed GABAergic potentiation^66–69^, hippocampal cultures were treated with IL-6 in the presence or absence of the KCC2 inhibitor VU0463271, and GABAergic transmission was recorded at 6 DIV (Fig. S5D). KCC2 blockade by VU0463271 did not prevent the IL-6-driven increase of GABAergic transmission (Fig. S5E), indicating that the increased GABAergic transmission induced by IL-6 precedes and drives the developmental GABA switch.

To support the occurrence of this mechanism in vivo, early potentiation of GABAergic transmission would be expected at a developmental stage preceding KCC2 upregulation. To test this, the expression levels of GABA-related genes and KCC2 transcripts (*Gad67*, *Slc32a1*, and *Slc12a5*) were quantified at an earlier developmental stage, in the hippocampus of P3 mice (Fig. 5H). We found that the two GABA-related genes, *Gad67* and *Slc32a1*, were significantly upregulated already at this stage (Fig. 5I). Accordingly, the synaptic GABAergic transmission at this stage was strongly enhanced as shown by the significant increase in the frequency of GABAergic postsynaptic currents (Fig. 5J, K). Notably, the KCC2 transcript *Slc12a5* levels were not changed at this stage (Fig. 5L). Since GABAergic transmission is still depolarizing in the hippocampus at P3^70^, an upregulation of GABA-mediated transmission should enhance electrical activity at this stage. In line with this hypothesis, we found that hippocampal network activity was strongly enhanced at P3 in IL-6 mice, with both MFPR and SNEs (Fig. 5M) frequency significantly increased compared to controls (Fig. 5N, Fig. S5F, G), consistent with enhanced excitatory GABAergic signaling. Furthermore, the pharmacological inhibition of KCC2 through bath application of VU0463271 did not induce any change in the electrical activity of hippocampal slices from P3 mice prenatally exposed to IL-6 (Fig. 5O, Fig. S5H), confirming that KCC2 expression changes do not precede or accompany, but rather follow the enhancement of GABAergic activity. Finally, to establish a causal link between the IL-6-induced enhancement of GABAergic transmission and the subsequent upregulation of KCC2, pups prenatally exposed to IL-6 and vehicle were treated with Picrotoxin between P1 and P5, and the KCC2 transcript *Slc12a5* expression was assessed at P5 (Fig. 5P). We found that the pharmacological inhibition of GABAergic signaling during this developmental window was sufficient to prevent the upregulation of KCC2 in IL-6 exposed mice (Fig. 5Q). Overall, these findings demonstrate that a transient IL-6 elevation at GD15.5 enhances early GABAergic transmission, promoting an initial surge in hippocampal network activity, which in turn accelerates the maturation of the GABA polarity developmental switch.

## Discussion

In this study, we demonstrate that a transient prenatal IL-6 elevation at GD15.5 alters early hippocampal electrical activity occurring in newborn mice by modulating the developing GABAergic system. The electrical activity that precedes sensory-driven information is a critical determinant of neuronal development across various brain regions^8,31,71^ and plays a pivotal role in shaping neuronal circuits^30^. In the hippocampus, the early pattern of electrical activity is characterized by synchronized firing across groups of neurons^72–75^ originating from distinct regions^6,39,76,77^. Using HD-MEA technology, which enables multineuronal recordings, we found that hippocampal neurons in acute brain slices from pups during the first postnatal week displayed synchronized LFPs engaging groups of neurons throughout the hippocampus. Prenatal IL-6 elevation at GD15.5 caused a biphasic modulation of this early hippocampal electrical activity: an initial potentiation of the entire network activity immediately after birth, at P3, followed by a reduction in LFP occurrence rate at P5, compared to control mice. Our results indicate that IL-6 induces this biphasic modulation by affecting two key processes: early GABAergic transmission and neuronal chloride homeostasis.

The initial increase of hippocampal network activity induced by IL-6 is ascribable to an early enhancement of synaptic GABA transmission, which represents the predominant synaptic component emerging in the developing hippocampus^39,58,78^. Specifically, leveraging neuronal cultures derived from the embryonic medial ganglionic eminence (MGE), that are enriched with GABAergic neurons, we highlighted the capability of this cytokine to directly target GABAergic neurons. Also, in line with the IL-6-driven intracellular signaling^19^, this modulation in vitro occurs in a STAT3-dependent fashion. It should be noticed that although our ex vivo and in vitro data strongly suggest a STAT3-dependent enhancement of GABAergic activity triggered by IL-6, direct in vivo evidence of this specific mechanism remains to be fully demonstrated. Further studies employing inducible, cell-type-specific conditional knockout models will be necessary to causally define the in vivo role of STAT3 in GABAergic neurons within this developmental window. Although several mechanisms could contribute to the enhancement of synaptic GABA transmission, we found that IL-6 leads to a robust transcriptional upregulation of two key GABA-related genes, *Gad67* and *Slc32a1*, encoding for two proteins involved in the synthesis of the GABA neurotransmitter (GAD67), and its loading into synaptic vesicles (the vesicular GABA transporter, VGAT), respectively. Consistent with the relevance of these proteins in GABA signaling, the genetic ablation of GAD67 significantly reduces GABAergic synaptic transmission^79^, whereas an upregulation of VGAT increases GABA release^80^. Considering the excitatory action of GABA signaling in the hippocampus at this specific developmental stage^70^, the upregulation of these two GABA-related genes mediated by IL-6 causes the initial potentiation of perinatal hippocampal neuronal activity.

Following this initial positive modulation, the hippocampal electrical activity of mice prenatally exposed to IL-6 undergoes a significant reduction in LFP event frequency within the next 48 hours. The high intracellular chloride concentration that characterizes the early postnatal stages of neuronal development sets the activity of hippocampal neurons and results in a depolarizing action of GABA signaling^3,4^. Indeed, we show that the hippocampal electrical activity is strongly reduced at early, but not later, stages when intracellular chloride was pharmacologically reduced by blocking NKCC1. This indicates that GABA-mediated excitatory activity strongly depends on elevated levels of intracellular chloride during this period. Here we found that immature neurons transiently exposed to IL-6, either in vivo or in vitro, undergo a premature and significant reduction of intracellular chloride levels caused by an early upregulation of the chloride extruder KCC2. Notably, following IL-6 treatment, while KCC2 expression remains unchanged immediately after birth (P3), both the transcript and membrane localization of the protein increase a few days later (P5), suggesting enhanced synthesis and chloride-exporting activity of the transporter.

The functional changes induced by prenatal IL-6 exposure in hippocampal slices at P5 reveal that, rather than altering episodes of synchronous network events (SNEs), IL-6 primarily affects the frequency of local field potentials (MFPR). While the occurrence of SNEs may reflect the intrinsic tendency of specific leader neurons to spontaneously fire^81–83^, the amplitude and frequency (MFPR) of LFPs may reflect the strength of synaptic excitatory signaling as previously reported^81,84,85^.

Since neuronal excitability is not altered by IL-6 pretreatment, the premature decrease in intracellular chloride levels, driven by early KCC2 expression, might reduce excitatory synaptic activity, consequently dampening LFP occurrence rate. Consistently, the blockade of the KCC2 transporter in IL-6 pretreated mice produced a selective increase in MFPR, indicating that KCC2 activity primarily affects the complexity, rather than generation, of SNEs. This effect is reminiscent of the pro-epileptic activity of KCC2 inhibitors observed in adulthood^86–88^, once the developmental GABA switch is complete. Importantly, the premature KCC2 upregulation disrupts the developmental acquisition of specific behavioral phenotypes in pups, including the capability to discriminate maternal odor and the number of USV calls, as pharmacological inhibition of the chloride transporter fully rescued these deficits. Notably, these behavioral impairments were detectable and reversible at P10 and not at P5 when KCC2 upregulation first occurs. This observation suggests that the early KCC2 upregulation represents the initial pathological event that subsequently alters the proper neurodevelopment trajectory leading to detectable behavioral impairments at later stages

The developmental GABA polarity switch constitutes a key biological process for the brain development^89^ and its alterations have been described in both genetic^90–93^ and environmental-related mouse models of neurodevelopmental disorders^94,95^. In the majority of these models, persistent depolarizing GABA signaling is present also at mature stages^90–95^. Here, instead, we report an acceleration of this process, leading to a premature lowering of intracellular chloride levels and an untimely shift of GABA signaling toward inhibition. An acceleration of the GABA polarity developmental switch has been reported in a genetic model of NDD based on a single mutation of Oligophrenin 1 (OPHN1), which displays an early overexpression of KCC2 with a precocious reduction of chloride load due to an impairment in the migrating neuroblasts^96^. More recently, early life stress in rats has been reported to accelerate the GABA switch in the prefrontal cortex of the offspring^97^. Altogether, our findings provide evidence supporting the idea that any deviation from the proper temporal sequence of neurodevelopmental events involving the GABAergic switch – whether delayed or accelerated – can critically disrupt network maturation and wiring in the offspring.

Finally, we show that the early potentiation of GABAergic transmission induced by IL-6 represents the first key event responsible for the subsequent precocious increase of KCC2 expression. In support of this view, we show that (i) the enhancement of the GABAergic transmission elicited by IL-6 precedes the GABA switch in acute brain slices and (ii) the blockade of the IL-6-dependent enhancement of the GABAergic transmission prevents the premature upregulation of KCC2 both in vivo and in vitro. Our evidence strongly supports the hypothesis that depolarizing GABAergic transmission, primarily involved in the early network activity^2^, constitutes one of the main molecular drivers for the developmental switch of GABA signaling from a depolarizing to hyperpolarizing action^55^. Besides promoting the GABA polarity switch, early GABA signaling exerts trophic action on several key developmental processes including the formation of glutamatergic synapses^7,98–100^. In line with this framework, blocking the IL-6-dependent enhancement of GABAergic transmission also prevents the subsequent increase in the glutamatergic transmission that was associated to brain hyperconnectivity and altered spatial learning in adult IL-6 mice^19^. Thus, prenatal IL-6 elevation can initially potentiate the early hippocampal network activity by enhancing GABAergic transmission which, in turn, promotes a premature GABA developmental switch and, at later stages, abnormal neuronal connectivity. Hence, our findings reveal that a maternal immune challenge precipitates the GABA developmental switch through an early potentiation of GABAergic transmission, derailing the precise temporal sequence critical for normal brain development. It will be of great interest to investigate whether other conditions previously associated with an accelerated GABA switch also share a common underlying mechanism involving immune dysregulation and increased inflammatory cytokines, such as IL-6. The immune-mediated disruption of the temporal orchestration of neuronal maturation may represent a shared substrate for the increased risk of neurodevelopmental and psychiatric disorders.

Prenatal and early postnatal life represent highly vulnerable periods characterized by a range of crucial biological processes that are essential for the proper development of neural circuits in adulthood. Given the complexity of these processes and their pivotal role in brain development, environmental perturbations – such as infections or physical or psychological stress – may result in profound and enduring detrimental impacts on mature neural circuits, potentially leading to behavioral abnormalities in adulthood. In this perspective, we have previously shown that prenatal IL-6 elevation leads to long-lasting detrimental effects in adult offspring, producing hippocampal hyperconnectivity and impaired hippocampal-dependent spatial memory, without affecting anxiety- or locomotion-related behaviors^19^. Given the ability of prenatal IL-6 to specifically target early hippocampal network activity through modulation of GABAergic signaling and chloride homeostasis, the present work adds a new piece of evidence to this complex phenomenon, suggesting that the long-lasting consequences of prenatal insults may originate from early disruptions of key developmental processes. However, while the present study demonstrates the functional and behavioral consequences of maternal IL-6 elevation in offspring up to P10, the specific and direct long-term behavioral alterations in fully mature offspring remain to be thoroughly evaluated with dedicated longitudinal studies.

IL-6 is a pleiotropic immune molecule that is produced and released by various systems in the body^101^ in response to a range of environmental stressors, including bacterial and viral infections^17,18,20,21^, as well as non-infectious stressful stimuli^102^. In this context, IL-6 can be viewed as a maternal molecular hub where several environmental stress factors intersect. Given the centrality of GABAergic signaling in brain development, the impact of IL-6 on this system may represent a common early event across various maternal insults, potentially leading to long-lasting effects on offspring development and behavior. Clarifying the molecular mechanisms that underlie this phenomenon could pave the way for the development of new druggable tools or alternative strategies for early intervention to reduce the risk of neurodevelopmental disorders in newborns exposed to maternal environmental insults.

## Methods

### Animals

All experiments were performed using mice C57BL/6 N (Charles River Laboratories) according to the guidelines established by the European Community Council (Directive 2010/63/EU of September 22nd,2010) and were approved by the Institutional Animal Care and Use Committee (IACUC, permission numbers 467 and 565) of the Humanitas Research Hospital and by the Italian Ministry of Health. Mice were housed in the animal facility under constant temperature (22 ± 1 °C) and humidity (50%) conditions with a 12 h light/dark cycle and were provided with food and water ad libitum. Pregnant females aged 3-6 months were used for experiments, and all embryos (E16-E18) and male offspring at specific ages were selected for the analysis.

### IL-6 injection in dams

At gestational day 15.5, pregnant mothers were intraperitoneally (IP) injected with 5 μg of IL-6 as previously described^19^. Saline solution was used as a control of injection. Only male offspring were selected for the in vivo and ex vivo analysis.

### Primary hippocampal cultures

Primary hippocampal neurons were established from E17.5 C57BL/6 N embryos as previously described^19^. Briefly, hippocampal regions were isolated from the brain in HBBS 1X (Hank’s Balanced Salt Solution) (Life Technologies), 1% Pen/Strep, 10 mM HEPES) and, after trypsinization, they were dissociated and plated onto 24 mm-diameter round glass coverslips, previously coated with 0.1% Poly-L-Lysine (Sigma-Aldrich) in Borate buffer (50 mM Boric Acid, 15 mM Borax) pH 8.5. 80000 cells were seeded onto each coverslip. Cultures were grown in Neurobasal medium supplemented with 2% B27 and 1% Glutamax (Life Technology) at 37 °C and 5% CO2. Neurons were treated at 1 DIV and 4 DIV before being tested.

### Primary Ganglionic and cortical cultures

Primary ganglionic eminences (GE) and cortical (CTX) from E14.5 C57BL/6 N embryos were established as previously reported^59,60^. Specifically, ganglionic eminences and cortical regions from the same embryo were dissected out in HBBS 1X (Hank’s Balanced Salt Solution) (Life technology), 1% Pen/Strep, 10 mM HEPES) and, after trypsinization, they were dissociated and plated onto 24 mm-diameter round glass coverslips, previously coated with 0.1% Poly-L-Lysine (Sigma-Aldrich) in Borate buffer (50 mM Boric Acid, 15 mM Borax) pH 8.5. 80000 cells were seeded onto each coverslip. Cultures were grown in Neurobasal medium supplemented with 2% B27 and 1% Glutamax, 1% Pen/Strep (Life Technology) at 37 °C and 5% CO2. Neurons were treated at 1 DIV and 4 DIV before being tested.

### Drugs and reagents

According to the type of experiment, the following reagents have been used: IL-6 (Peprotech), Galiellalactone (BioAustralis), Stattic, Bicuculline, 6-Cyano-7-nitroquinoxaline-2,3-dione (CNQX), Tetrodotoxin (TTX), D-2-amino-5-phosphonovalerate (APV), VU0463271, Picrotoxin (Tocris Bioscence).

### Calcium imaging

Calcium imaging recordings were made in cultured neurons from 4 DIV to 7 DIV to assess the GABAergic switch temporal window and at 6 DIV for all other experiments. Cells were loaded with Oregon Green™ 488 BAPTA-1, AM, cell-permeant (Life Tech) 5 µM in culture medium at 37 °C for 45 min, then washed with external solution [Krebs’–Ringer’s–HEPES (KRH)] containing in mM: 125 NaCl; 5 KCl; 1,2 MgSO4; 1,2 KH2PO4; 25 HEPES; 6 Glucose; 2 CaCl2; pH 7,4. The coverslip was then mounted onto a round chamber for the perfusion system. Recording chambers were placed on the stage of an IX-71 inverted microscope (Olympus, Hamburg, Germany) equipped with an EMCCD (electron-multiplying CCD) camera (Quantem 512×512, Photometrics). Illumination was obtained using a light-emitting diode LED (Cairn Research, Optoled Lite), with a 20X objective. Regions of interest (ROIs) of about 15-pixel area were drawn on the cell cytoplasm of virtually all the cells in the recorded field. About 20 cells for each Field Of View (FOV) and 3 FOV for each condition. Time-lapse recording of calcium dynamics was performed with an acquisition rate of 5 Hz for 600 s and analyzed offline with MetaFluor software (Molecular Devices). A puff of 100 µM GABA was then administered to assess GABA-dependent Calcium transients. Calcium responses were measured as ΔF (Fmax-F0) compared to the baseline (F0), where F0 represented the baseline signal, and Fmax the signal after the GABA stimulus. ΔF/F0 was considered as the final value describing the fluorescence increase after the GABA stimulus. Values ΔF/F0 < 0,10 were not considered as signals. The number of responding cells was then plotted.

### Chloride imaging recordings

Intracellular chloride measurements were performed using the N-(ethoxycarbonylmethyl)-6-methoxyquinolinium bromide (MQAE) (ThermoFisher) chloride sensor. Briefly, hippocampal neurons from selected time points and experimental conditions were loaded with 5 μM MQAE for 45 min at 37 °C in culture medium. Coverslips were then rinsed with external solution (Krebs’–Ringer’s–HEPES (KRH) containing in mM: 125 NaCl; 5 KCl; 1,2 MgSO4; 1,2 KH2PO4; 25 HEPES; 6 Glucose; 2 CaCl2; pH 7,4 and transferred to the recording chamber for acquisitions. Olympus IX81 inverted microscope, 20× dry objective (Olympus, UPLFLN NA 0.5) provided with an MT20 widefield source and control system with excitation at 340 nm and an emission filter centered at 500 nm, was used. EXcellence RT software (Olympus) was used to set up experiments and sample recordings. For each sample, a minimum of eight fields of interest (mean of cells observed per field, 20) were chosen to perform live imaging recordings. Offline analyses were then performed after defining ROIs centered to cell bodies and measuring mean MQAE intensity during the time recording window.

### Electrophysiological analysis

#### Ex vivo acute hippocampal slices

C57BL/6 N male mice at P6 were anesthetized with isofluorane at 4% by inhalation and rapidly decapitated. Brains were removed and placed in ice-cold solution containing the following (in mM): 87 NaCl, 21 NaHCO3, 1.25 NaH2PO4, 7 MgCl2, 0.5 CaCl2, 2.5 KCl, 25 D-glucose, and 7 sucrose, equilibrated with 95% O2 and 5% CO2 (pH 7.4). Coronal hippocampal slices (300 μm thick) were cut with a VT1000S vibratome (Leica Microsystems). Slices were incubated at room temperature for at least 1 h, in the same solution as above, before being transferred to the recording chamber. During experiments, slices were superfused at 1–2 mL/min with artificial cerebrospinal fluid (aCSF) containing the following (in mM): 129 NaCl, 21 NaHCO3, 1.25 NaH2PO4, 1.8 MgSO4, 1.6 CaCl2, 3 KCl, 10 D-glucose, equilibrated with 95% O2 and 5% CO2 (pH 7.4). Cells were examined with a BX51WI upright microscope (Olympus) equipped with a water immersion differential interference contrast (DIC) objective and an infrared (IR) camera (XM10r Olympus). Neurons were voltage- or current-clamped with a Multiclamp 700B patch-clamp amplifier (Molecular Devices, Union City, CA) and recorded at 28–30 °C temperature. Low-resistance micropipettes (4-6 MΩ) were pulled from borosilicate. The cell capacitance and series resistance were left uncompensated. Experiments in which series resistance changed by more than 20% and did not remain below 20 MΩ (an average of 10–15 MΩ) were discarded. Signals were low-pass filtered at 2 kHz, sampled at 20 kHz and analyzed with Digidata 1550B (Molecular Devices). Recordings were performed in visually identified CA1 pyramidal neurons. Synaptic basal transmission was recorded at −70 mV using the following internal solution: (in mM) 68 K-gluconate, 68 KCl, 2 MgSO4, 20 HEPES, 2 ATP, 0.5 GTP, pH 7.2. GABAergic transmission was pharmacologically isolated using 6-cyano-7-nitroquinoxaline-2,3-dione (CNQX; 20 μM), 2 R)-amino-5-phosphonovaleric acid (APV; 50 μM) and TTX (1 µM), whereas Glutamatergic transmission was isolated with bicuculline (20 μM) and TTX (1 µM). Spontaneous synaptic transmission was recorded under the same conditions but without any pharmacological blockade and then bath-perfused with specific synaptic blockers. Intrinsic excitability was recorded in the presence of CNQX 20 μM, APV 50 μM and bicuculline (20 μm) using the following internal solution (in mM): 135 K-gluconate, 5 KCl, 2 MgCl2, 10 HEPES, 1 EGTA, 2 ATP, 0.5 GTP, pH 7.4. Current-clamp recordings were performed at a holding potential of -70mV, and action potential firing was induced by injecting current steps of 5 pA lasting 500 ms.

#### In vitro primary hippocampal, GE and cortical neurons

Patch-clamp recordings were performed in hippocampal neurons at 6 DIV in an extracellular solution with the following composition (in mM): 130 NaCl, 5 KCl, 1.2 KH2PO4, 1.2 MgSO4, 2 CaCl2, 25 HEPES, and 6 Glucose, pH 7.4. GABAergic mPSCs were recorded in the presence of CNQX 25 μM, (2 R)-amino-5-phosphonovaleric acid (APV; 50 μM) and TTX (1 µM) using the following internal solution (in mM): 68 K-gluconate, 68 KCl, 2 MgSO4, 20 HEPES, 0.5 EGTA, 4 ATP, 0.2 GTP, pH 7.2. Glutamatergic mPSCs were recorded in the presence of Bicuculline (20 μM), APV (50 μM) and tetrodotoxin (TTX; 1 mM) using the following internal solution (in mM): 135 K-gluconate, 5 KCl, 2 MgCl2, 10 HEPES, 1 EGTA, 2 ATP, 0.5 GTP, pH 7.4. Glass pipettes (World Precision Instruments, #PG52151-4; 1.5 ×1.0 mm, 4”) of 4-6 MΩ were used as recording electrodes, pulled with a vertical puller (Narishige PC-10) by a 2-steps temperature protocol (step 1: 51.9 °C; step 2: 44.1 °C). Both Glutamatergic and GABAergic basal synaptic transmission were recorded at −70 mV as holding potential in the presence of specific synaptic blockers to pharmacologically isolate these two synaptic components. Electrical signals were amplified by a Multiclamp 200 B (Axon Instruments), filtered at 2 kHz, digitized at 20 kHz with a DIGIDATA 1440 and stored with pClamp 10 (Axon Instruments). The resting potential was calculated at I = 0 in current-clamp configuration, whereas membrane resistance, capacitance and access resistance were directly obtained in voltage-clamp configuration using a 10-mV membrane test pulse at a holding potential of −70 mV. Only cells with an access resistance <20 MΩ were considered for the analysis of synaptic basal transmission. Access resistance was continuously monitored during the experiment, and those cells in which access resistance changed of more than 10% were rejected. Cells with a leak current above 100 pA were excluded from the analysis. The analysis of both basal synaptic transmissions was performed with Clampfit (Molecular Device). The Glut/GABA ratio in GE and cortical cultures was quantified by simultaneously monitoring the frequency of Glutamatergic and GABAergic basal synaptic transmission in the same cell, using an internal solution containing (in mM): 130 Cs-gluconate, 1 EGTA, 20 HEPES, 2 MgCl₂, 4 NaCl, 2 Mg-ATP, and 0.5 Tris-GTP; pH 7.2. Recordings were performed in the presence of 1 μM TTX, holding the membrane potential at –70 mV for Glutamatergic mPSCs, then switching to +20 mV to record GABAergic mPSCs.

#### Events detection and electrophysiological analysis

The analysis of electrophysiological recordings was performed using Clampfit software (Molecular Devices). For synaptic transmission, traces were low-pass filtered at 1 kHz (Bessel filter), and baseline was corrected. Synaptic events were detected using a peak threshold of 10 pA, corresponding to approximately four times the baseline noise (SD ~ 2.5 pA). For intrinsic excitability, traces were low-pass filtered and the threshold for AP detection was set at 0 mV. The kinetic properties of AP were calculated on the first AP elicited with the minimum injected current.

#### Multi-Electrode-Arrays (MEA) on brain slices

C57BL/6 N male mice at different postnatal (P) stages (P5-P10-P15) from either Vehicle or IL-6-injected dams were anesthetized with isoflurane at 4% by inhalation and rapidly decapitated. Brains were quickly removed and placed in ice-cold solution with the following composition (in mM): 87 NaCl, 21 NaHCO3, 1.25 NaH2PO4, 7 MgCl2, 0.5 CaCl2, 2.5 KCl, 25 D-glucose, ascorbic acid, and 7 sucrose, equilibrated with 95% O2 and 5% CO2 (pH 7.4). Coronal hippocampal slices (300 µm) were cut using a VT1000S vibratome (Leica Biosystems Nussloch GmbH) and then incubated at room temperature for at least 1 h in artificial cerebrospinal fluid (aCSF) with the following composition (in mM): 129 NaCl, 21 NaHCO3, 1.25 NaH2PO4, 1.8 MgSO4, 1.6 CaCl2, 3 KCl, 10 D-glucose, equilibrated with 95% O2 and 5% CO2 (pH 7.4). Multisite electrophysiological recordings were performed using 2D high-density multi-electrode arrays (HD-MEA, 3Brain AG, Switzerland) integrating 4096 recording electrodes of 42 µm pitch distributed in a 2.6 × 2.6 mm^2^ total recording area (HD-Arena). Slices were positioned on the chip using a platinum anchor with 100 μm-width wires and recorded in a solution with the following composition (in mM): 129 NaCl, 21 NaHCO3, 1.25 NaH2PO4, 1.8 MgSO4, 1.6 CaCl2, 3 KCl, 0,1 4AP, 10 D-glucose, equilibrated with 95% O2 and 5% CO2 (pH 7.4). After 10 minutes of equilibration, hippocampal activity was then recorded for 5 minutes at a sampling rate of 18 kHz in standard aCSF solution, using a 10 Hz high-pass filter. During the recordings, slices were constantly superfused at 3.0 mL/min with aCSF solution. When indicated, following baseline acquisition of network activity, brain slices were bath-perfused with either VU0463271 (20 µM, Tocris 4719) or Bumetanide (20 µM, Tocris 3108) for 15 min to evaluate the acute effects of these drugs on electrical activity. At the end of each recording session, a high-definition (HD) image of the slice was taken using an HD digital microscope (Bysameyee) and subsequently superimposed onto the activity map, ensuring a precise electrical-anatomical correlation. The exact identification of the specific hippocampal subfields activated during the electrical activity was not feasible with this approach. Local Field Potential (LFP), Synchronous Network Events (SNEs) and synchronicity were the main electrophysiological features considered for the characterization of the hippocampal slices’ activity. Recordings and LFP detection were performed using BrainWave software from 3Brain and referred to as Mean Field Potential Rates (MFPR) values. LFPs were identified using the Hard Threshold algorithm, (High Threshold=200 µV, Low Treshold = −200 µV, Energy Window=40 ms, Max Wave Duration=80 ms, refractory period=5 ms). Only electrodes with an MFPR greater than 0.8 Field Potential (Fp)/minutes have been considered for the analysis. SNEs were detected using a custom Python script with a histogram-based approach^103^. Frequencies across all active electrodes were averaged within each recording. Trace timestamps were binned (200 bins) and synchronous events were identified as peaks in the histogram that exceeded the dynamic threshold of 40% of all active monitored electrodes. Boundaries of each event were extended in both directions until the histogram count fell to 0. Synchronization between pairs of electrodes was evaluated with the Spike Time Tiling Coefficient (STTC) using a custom Python script^103^. The STTC provides a measure of the temporal correlation between two spike (LFP) trains, accounting for both the proportion of coincident spikes and the temporal precision of spike timing. For each pair of spike trains, the STTC was calculated with a temporal window of 10 ms. The resulting STTC values for all electrode pairs were compiled into a matrix, from which the mean STTC was derived to quantify overall synchronization levels. LFP mean values, SNE mean values and STTC values were plotted using Prism 10.0 (GraphPad Software, Inc, https://www.graphpad.com). Data and graphs were presented as mean ± SEM. Data analysis was conducted using a non-parametric variant of the *T*-test (Mann–Whitney test) to evaluate both frequency and STTC. For each experimental session, recordings were performed by simultaneously comparing two conditions (i.e., two mice) on the same day, in order to standardize recording conditions. The custom Python scripts developed and used for these analyses^103^ have been deposited in a public repository; details regarding code accessibility are provided in the Code Availability section.

### Two-photon chloride imaging on brain slices

C57BL/6 N mice at P5 were anesthetized with isoflurane and decapitated. Brains were removed from skull in ice-cold cutting solution (117 mM NaCl, 2.5 mM KCl, 1.25 mM NaH_2_PO_4_, 3 mM MgSO_4_, 0.5 mM CaCl_2_, 25 mM NaHCO_3_, and 25 mM d-glucose, pH 7.4, oxygenated with 95% O_2_ and 5% CO_2_). Coronal slices (250 μm thick) were prepared in a vibratome (VT1000S Leica Microsystem) in the same cutting solution. Slices were then allowed to recover in artificial cerebrospinal fluid (ACSF: 117 mM NaCl, 2.5 mM KCl, 1.25 mM NaH_2_PO_4_, 1 mM MgSO_4_, 2 mM CaCl_2_, 25 mM NaHCO_3_, and 25 mM glucose, pH 7.4, oxygenated with 95% O2 and 5% CO2) first for 30 min at 35 °C and then for at least 30 additional minutes at room temperature. For imaging of neuronal intracellular Cl^−^ concentration, slices were loaded with 5 mM MQAE (Molecular Probes) in the dark for 30 min in oxygenated ACSF at 37 °C. Slices were then transferred to the recording chamber and perfused with oxygenated ACSF at 30 °C for 5 min before imaging. Images were taken with a SP5 upright scanning confocal microscope (Leica Microsystems) coupled to a Ti:sapphire laser (Chameleon Ultra, Coherent) and equipped with a water-immersion objective (25×, NA 0.95; Leica) and a 525/50 nm band-pass emission filter. MQAE was two-photon excited at 750 nm. Acquisitions were performed by simultaneously recording two samples (i.e., two mice) on the same experimental day, to ensure identical acquisition settings within each experimental set. This approach allows a reliable comparison of the relative intracellular chloride levels between the two samples.

All excitation and acquisition parameters were kept constant throughout the experiments. Image analysis was performed with LAS-AF software (Leica) by measuring the mean fluorescent intensity of regions of interest placed on the pyramidal layer of the hippocampal CA1 region. Fluorescence Lifetime Intensity Measurements (FLIM) were provided for P5 vs P15 comparison. Regions of Interest (ROIs) were centered on the CA1 pyramid neurons cell soma. Curve fitting was adapted to monoexponential components and Tau lifetime (ns) was calculated.

### RNA extraction and qRT-PCR

Frozen hippocampi from P5 offspring were gently thawed on ice and then subjected to mechanical disruption with homogenizing beads on an automatic Tissue Lyser, in 500 μl TRI Reagent® (Zymo Research). RNA was isolated with RNA Direct-Zol™ MiniPrep Isolation Kit (Zymo Research), according to the manufacturer’s guidelines. RNA was eventually eluted in 25 μl DNAse/RNAse-free water and quantified with a NanoDrop 2000c spectrophotometer (Thermo Fisher Scientific) for RNA concentration and 260/280 nm optical density ratios.

500 ng RNA for each condition underwent reverse transcription into cDNA with High-Capacity cDNA RT kit (Applied Biosystems). Quantitative Real-time polymerase chain reaction (qRT-PCR) was performed with TaqMan detection kit (TaqMan Fast Universal PCR Master Mix(2x), no AmpErase UNG, ThermoFisher) onto qRT-PCR Viia7 software system (Applied Biosystems) in a final volume of 10 μl. Each gene was subjected to at least duplicate measurements and data analyses were performed with the comparative ΔCT method. mRNA measurements for each target gene were normalized to the housekeeping gene *Gapdh*. The following FAM-MGB TaqMan assays were used (Applied Biosystems): mouse *Slc32a1* (vGAT), Mm00494138_m1; mouse *Gad67* Mm04207432_g1; mouse *Gphn* (gephyrin) Mm00556895_m1; mouse *Slc12a2* (NKCC1) Mm01265951_m1; mouse *Slc12a5* (KCC2) Mm00803929_m1; mouse *Dlx* Mm00438427_m1, mouse *Slc17a7* (vGLUT1) Mm00812886_m1; mouse *Il6* Mm00446190_m1; mouse *Tnf* Mm00443258_m1; mouse I*l1b* Mm00434228_m1; mouse *Ifn* Mm04207507_gH; mouse *Ccl11* Mm00441238_m1; mouse *Ccl4* Mm00443111_m1; mouse *Cxcl10* Mm00445235_m1; mouse *Foxp3* Mm00475162_m1; mouse *Nos* Mm00440502_m1; mouse *Ccl2* Mm00441242_m1; mouse *Il17ra* Mm00434214_m1; mouse GAPD(GAPDH) VIC-MGB Endogenous Control.

### Biochemistry

4 DIV GE and CTX neurons, cultured in standard incubation conditions (37 °C, 5% CO2), were treated with 20 ng/ml IL-6 for 20 minutes before lysis; the control group received no treatment. For total protein extraction, neurons were washed in ice-cold PBS and lysed by scraping in 65 μl/dish lysis buffer (10 mM Hepes-Na2 pH 7.4, 2 mM EDTA pH 8.0, 1% SDS) supplemented with protease and phosphatase inhibitors (Roche), keeping neurons on ice for the entire lysis procedure. Lysates were sonicated for 5 cycles (30 s on, 10 s off) and centrifuged for 10’ at 4 °C 15,000 × *g*. Protein concentration was estimated using Bicinchoninic Acid Assay (BCA) kit (ThermoFisher Scientific) and 30 μg of samples were loaded with Loading Sample Buffer (final concentration 25 mM Tris-HCl pH 6.8, 1% SDS, 10% glycerol, 1.25% β-mercaptoethanol); boiled for 5 minutes at 95 °C and run for SDS-PAGE. After electrophoresis, samples were transferred to a nitrocellulose membrane (Bio-Rad, mini size) using a transfer apparatus according to the manufacturer’s protocols (Bio-Rad). The membrane was rinsed in TBS-T (20 mM Tris-HCl pH 7.5, 150 mM NaCl, 0,1% Tween20) before blocking with 5% BSA (Sigma-Aldrich) in TBS-T for 1 h. Membranes were incubated overnight with primary antibodies diluted in 5% BSA in TBS-T. The following primary antibodies were used: rabbit anti-pSTAT3 Tyr705 (1:1000; Cell Signaling, #D3A7), rabbit anti-STAT3 (1:1000; Cell Signaling, #D3Z2G), rabbit anti-GAPDH (1:1000; Synaptic System, #247002). Membranes were washed with TBS-T before incubation with anti-rabbit HRP-conjugated secondary antibodies (1:5000; Thermo Fisher Scientific) for 2 hours. Immunodetection was performed with Clarity Western ECL Substrate (Bio-Rad) or SuperSignal West Femto (Protein Biology) and analyzed through Chemidoc apparatus (Bio-Rad); image analysis was performed via ImageJ software (Fiji). Uncropped and unprocessed scans of the western blot shown in Fig. 4J are provided in the Source Data file.

After the acquisition of phosphorylated-STAT3, membranes were stripped in a stripping solution (0,2 M Glycine, 0,1% SDS, 1% Tween20, pH 2.2) twice for 5 minutes, washed with TBS and incubated with blocking solution before staining for total STAT3.

### Immunofluorescence analysis

Hippocampal slices: Mice aged P5 were deeply anesthetized with xylazine/ketamine mixture (100 mg/kg and 10 mg/kg; intraperitoneal injection) and transcardially perfused with 4% PFA in 0.1 M phosphate-buffered saline (PBS), pH 7.4 and additionally post-fixed overnight. Coronal 50μm -thick slices were cut with a VT1000S vibratome (Leica Microsystems). Slices were washed in cold PBS1X; permeabilization and nonspecific binding were performed with 1% BSA, 0.3% Triton-X in 1X PBS for 1 h 30 min at room temperature. Primary antibodies against KCC2 (rabbit anti-KCC2, 1:500, Millipore, #07-432) and NeuN (guinea pig anti-NeuN, 1:500; Abcam, #ab134014) were incubated in 1% BSA, 0.1% Triton-X in 1X PBS, for two overnight at 4*C. Slices were then rinsed 3 times with 1X PBS and incubated with secondary antibodies conjugated to Alexa Fluor-488, 647 (1:500, Thermo Fisher) in 1% BSA, 0.1% Triton-X in 1X PBS, for 2 h at room temperature, in the dark. Slices were then washed, counterstained with Hoechst-33342 (1:1000, Thermo-Fisher) and mounted with Fluorsave mounting medium (Millipore). Confocal images were acquired using a Leica SP8II laser scanning confocal microscope equipped with an HC PL APO CS2 40x/1.30 oil objective. For KCC2 imaging at least three regions in the CA1 hippocampal subfield were analyzed for each section and three sections were used for each animal. Single-plane images were acquired using a 40x oil immersion lens on the NeuN focal plane. The parameters of acquisition (laser power, pinhole, gain, offset) were kept constant among groups. ROIs were placed on the borders of the neuronal somas within the CA1 fields. KCC2 mean fluorescence intensity (MFI) was measured at the endpoints (membrane) and at the center (cytoplasm, baseline) of the ROI diameter. The KCC2 fold was considered as the difference between plasma membrane versus cytoplasm intensity against the baseline intensity. The analyzed regions of one animal were averaged and considered as a single data point. For c-Fos staining, sixty minutes after the behavioral task, animals were deeply anesthetized with ketamine and xylazine (100 mg/kg and 10 mg/kg; intraperitoneal injection) and transcardially perfused with 4% PFA in 0.1 M phosphate-buffered saline (PBS), pH 7.4. Brains were removed and post-fixed overnight in 4% PFA at 4 °C. Coronal sections (40 μm thick) were cut using a sliding vibratome (Leica Microsystems) and processed for immunofluorescence. Free-floating sections were first incubated in a blocking solution containing 1% bovine serum albumin (BSA) in PBS with 0.3% Triton X-100 for 90 minutes at room temperature (RT). Sections were then incubated with a rabbit anti-c-Fos primary antibody (1:1000, Synaptic System, #226003) diluted in blocking buffer (PBS Triton 0.1%). Incubation was carried out for 72 h at 4 °C under constant shaking. After washes with PBS Triton 0.1%, sections were incubated for 2 h at RT with the appropriate secondary antibodies diluted in PBS with 0.1% Triton X-100 (e.g., Alexa Fluor 647-conjugated goat anti-rabbit IgG, X). Slices were then washed, counterstained with Hoechst-33342 (1:1000, Thermo-Fisher) and mounted with Fluorsave mounting medium (Millipore). Confocal images were acquired using a Leica SP8II laser scanning confocal microscope equipped with an HC PL APO CS2 20x/0.75 dry objective. Analyses were focused on the hippocampal CA1 region. c-Fos positive cells in the CA1 region of the hippocampus were manually quantified in at least two images per hemisphere for each animal using the Cell Counter plugin in Fiji (ImageJ). Regions of interest (ROIs) were manually delineated around the pyramidal cell layer of the CA1 subfield, and c-Fos positive cells within each ROI were counted. Data are expressed as cells per mm² and values obtained from individual sections were averaged to yield one representative value per mouse.

Primary hippocampal, ganglionic eminences (GE) and cortical neurons: 5 DIV hippocampal, GE and cortical neurons from Ctrl or IL-6-treatment condition were fixed with 4% PFA in PBS1X and sucrose solution for 20 min and permeabilized with 0.3% Triton-x 100 in PBS1X for 10 min. Nonspecific binding sites were blocked by incubation for 30 min with 5% NGS and 0.1% Triton-X in PBS1X before a 2-h incubation at room temperature with primary antibodies. Neurons were washed 3 times with PBS1X and incubated with secondary antibodies conjugated to Alexa Fluor (488, 568, or 633) for 45 min at room temperature. Coverslips were washed 3 times in PBS1X, counterstained with Hoechst to reveal nuclei and mounted onto slides with Fluorsave. The following primary antibodies were used: mouse anti-B3 tubulin (1:500, Thermo Fisher, #MA1-118); guinea pig anti-Map2 (1:1000, Synaptic Systems, #188004) and rabbit anti-GABA (1:1000, Thermo Fisher, # PA5-32241).

### Ex vivo FACS measurements

Spleen and brain were collected from male P5-6 mice born to IL-6 and Vehicle- injected dams and mechanically digested on 70 μm cell strainers with PBS 1X to obtain a single-cell suspension. The single cell suspension was stained with Aqua Fixable Viability dye (Invitrogen) and the following cell surface marker antibodies: CCR2-BV421 (clone SA203G11, Biolegend, cat#150605), F4/80-AlexaFlour488 (clone T45- 2342, BD, cat#567201), Ly6C APC-Cy7(clone AL-21, BD, cat#560596), CD49b-BV711 (clone HMa2, BD, cat#740704), Ly6G-BUV563 (clone 1A8, BD, cat#612921), MHCII-BUV737 (clone2G9, BD, cat#748708), CD3-PE-Cy7 (clone 145-2C11, Invitrogen, cat#25-0031-82), CD19-BUV661 (clone 1D3, BD, cat#612971), CD45-BV605 (clone 30-F11, Biolegend, cat#103155), CD11b-BUV395 (M1/70, BD, cat#563553), CD11c-BV786 (clone HL3, BD, cat#563735). Cells were fixed and permeabilized with the FoxP3 transcription factor buffer kit (eBioscience). Stained samples were acquired on BD FACSymphony A5. Flow cytometric compensation and gating were performed using FlowJo version 10.

### Maternal odor preference test

Maternal Odor Preference (MOP) task was conducted on P5-P6 and P9-P10 C57BL/6 N male mice exposed to vehicle or IL-6 at E15.5. On the day of the behavioral experiments, we I.P. injected pups with either VU0240551 100ug/kg or its vehicle (DMSO) and kept them with their dams for at least twenty minutes before starting the test. The task consisted of five /trials with an inter-trial interval of 120 s. Each trial began with placing one pup in the center of a clean home cage-like arena (clear Plexiglas cage, 15 × 30 × 11 cm) containing two Petri dishes (diameter: 6 cm, height: 1.5 cm) with either clean or home cage bedding on opposite sides. Home cage bedding remained unchanged for at least one week before the experiments. The pup was allowed to freely explore the arena for 80 s, then placed back in the home cage but separated it from the dam for 120 s until the subsequent trial began. Animals were video-recorded, and locomotion was automatically analyzed using Ethovision. To avoid artifacts, only the last 60 s of each video recording were tracked. The arena was divided into virtual maternal- and clean-odor zones separated by a central neutral zone of about 3 cm. The time spent in the maternal- and the clean-odor zones was automatically scored when the center of the pups’ body was inside the zone. The data are expressed as cumulative time spent in either the mother- or clean-odor zone during the five trials. Maternal preference was quantified as time spent in the “mother” zone minus the combined time spent in the “clean” and “neutral” zones. 

### Ultrasonic vocalization test

The ultrasonic vocalization test was conducted on male offspring aged P5-6 and P9-P10 according to the experimental design. On the day of testing, breeding cages containing dams with litters were transferred to the testing room and habituated for 30 min. Before measurement of an individual litter, a beaker was cleaned once with 70% EtOH and dried using paper towels. After a 5 min evaporation period, a clean paper towel was placed in the beaker. One pup at a time was individually separated from the nest and placed into the beaker on top of the clean paper towel within a light-proof Styrofoam box. Vocalization profiles within the 0–125 kHz spectrum were recorded for 3 min using an Avisoft Bioacoustic UltraSoundGate 116Hb recorder with a condenser microphone or a Metris Sonotrack control unit with a microphone. After measurement, the pup was returned to its nest. After each pup of a litter, the paper towel was removed, and a fresh paper towel was placed inside the jar.

### Statistical analysis

The numerical data shown in the figures are presented as means ± SEM. The normal distribution of experimental data was assessed using Kolmogorov-Smirnov and Shapiro–Wilk normality tests. If not specifically indicated, to compare two normally distributed sample groups, the Student’s unpaired two-tailed *t-test* was used. To compare two sample groups that were not normally distributed, we used Mann–Whitney’s non-parametric test. To compare more than two normally distributed sample groups, we used one-way ANOVA, followed by Tukey’s multiple comparisons test. To compare more than two groups that were not normally distributed, we used one-way ANOVA on ranks followed by Dunn’s multiple comparison test. One sample *t* test or Wilcoxon Signed Rank Test were used in some experiments to analyze normally distributed or non-normally distributed datasets, respectively.

Behavioral tests were analyzed using Two-Way ANOVA followed by between- group Ŝidàk’s multiple comparisons. Statistical analysis was performed using GraphPad (Prism), Python 3.11, and Origin 8 (OriginLab) software.

### Reporting summary

Further information on research design is available in the Nature Portfolio Reporting Summary linked to this article.

## Supplementary information

Supplementary Information

Reporting Summary

Transparent Peer Review file

## Source data

Source Data

## Data Availability

All data generated in this study are provided in the Supplementary Information and in the Source Data file. Source data are provided in this paper. Source data are provided with this paper.

## Code availability

The custom Python code developed and used for SNE detection and STTC analysis in this study is publicly available through Zenodo at https://doi.org/10.5281/zenodo.21909384.
