# Supplementary material for: Elevated prenatal IL-6 levels disrupt early patterns of hippocampal electrical activity and chloride homeostasis by targeting GABAergic signaling at perinatal stage
Source: Nat Commun. 2026 Oct 15;17:11627. doi: 10.1038/s41467-026-78479-9 (PMC13755421; doi:10.1038/s41467-026-78479-9)
Supplement: Supplementary file 1 [file 41467_2026_78479_MOESM1_ESM.pdf]

Elevated prenatal IL-6 levels disrupt early patterns of hippocampal electrical activity and chloride homeostasis by targeting GABAergic signaling at perinatal stage

Desiato et al,

Supplementary information

Figure S1

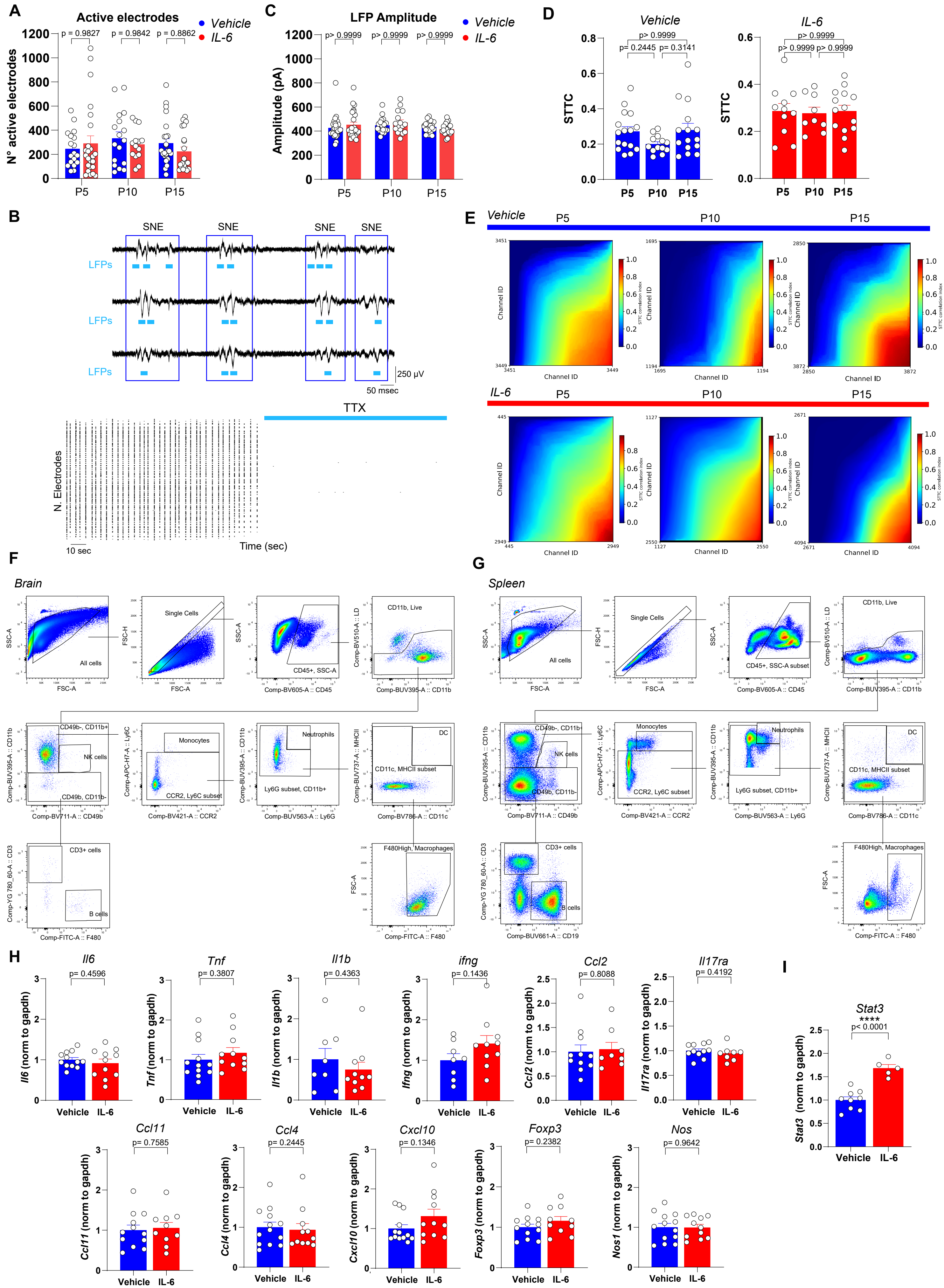
A
Active electrodes
Vehicle
IL-6
p = 0.9827
p = 0.9842
p = 0.8862
N° active electrodes
P5
P10
P15
C
LFP Amplitude
p> 0.9999
Amplitude (pA)
D
Vehicle
IL-6
p> 0.9999
p= 0.2445
p= 0.3141
STTC
B
SNE
LFPs
250 µV
50 msec
TTX
N. Electrodes
10 sec
Time (sec)
E
Channel ID
STTC correlation index
F
Brain
G
Spleen
All cells
Single Cells
CD45+, SSC-A
CD45+, SSC-A subset
CD11b, Live
CD49b-, CD11b+
NK cells
CD49b, CD11b-
Monocytes
CCR2, Ly6C subset
Neutrophils
Ly6G subset, CD11b+
DC
CD11c, MHCII subset
CD3+ cells
B cells
F480High, Macrophages
H
Il6
p= 0.4596
Tnf
p= 0.3807
Il1b
p= 0.4363
ifng
p= 0.1436
Ccl2
p= 0.8088
Il17ra
p= 0.4192
Ccl11
p= 0.7585
Ccl4
p= 0.2445
Cxcl10
p= 0.1346
Foxp3
p= 0.2382
Nos
p= 0.9642
I
Stat3
****
p< 0.0001
Vehicle
IL-6

Figure S1. MEA recordings set up and characterization of immune cells in IL-6-exposed P5 mice (Related to Figure 1).

A. Histograms showing the number of active electrodes across the experimental conditions (Vehicle vs IL-6) at different postnatal stages (P5, P10, P15). Dots represent n= 18, 17, 24 vs n= 24, 14, 20 slices from N=5, 4, 4 Vehicle and N=5, 4, 4 IL-6 exposed mice. Data are presented as mean ± SEM. One way ANOVA, ns > 0.05.

B. Representative raster plot from MEA recordings depicting LPFs and SNEs events (top) and the occlusion of network activity after TTX acute administration (bottom).

C. Histograms showing the amplitude of LPFs across the experimental conditions (Vehicle vs IL-6) at different postnatal stages (P5, P10, P15). Dots represent n= 30, 18, 22 vs n= 25, 16, 20 slices from N=5, 4, 4 Vehicle and N=5, 4, 4 IL-6 exposed mice. Data are presented as mean ± SEM. Kruskal Wallis test, followed by Dunn's multiple comparison, ns > 0.05.

D-E. Histograms (**C**) and relative representative plots (**D**) showing the Spike Time Tiling Coefficient (STTC) of Vehicle (**blue**) and IL-6 (**red**) exposed mice at different developmental stages. Dots represent n = 16, 13, 15 vs n= 11, 9, 14 slices from N= 5, 3, 3 Vehicle and N= 5, 3, 3, IL-6 exposed mice. Data are presented as mean ± SEM. Kruskal Wallis test, followed by Dunn's multiple comparison, ns > 0.05.

F-G. Representative fluorescent activated cell sorting (FACS) complete gating strategy of F4/80+ cells representing macrophages, CD3+ T cells together with B cells isolated from the brain (**E**) or the spleen (**F**) of P5 mice born to IL-6 or Vehicle injected mothers.

H. Quantification of mRNA relative expression of cytokines and chemokines, from top left to right bottom: intelelukin-6 il6, tumor necrosis factor Tnf, intelelukin-1beta Il1b, interferon-gamma Ifng, C-C motif chemokine 2 Ccl2, interleukin 17 receptor a Il17ra, C-C motif chemokine 11 Ccl11, C-C motif chemokine 4 Ccl4, C-X-C Motif Chemokine Ligand 10 Cxcl10, forkhead box P3 Foxp3, Nitric Oxide Synthase 1 Nos, in the hippocampi of Vehicle and IL-6 exposed mice. Data are presented as mean ± SEM of N=14 Vehicle vs N=11 IL-6 mice from at least three independent experiments. Two-tailed Unpaired *t* test, ns > p 0.05.

I. Quantification of *Stat3* mRNA relative expression in the hippocampi of Vehicle and IL-6 exposed P5 mice. Data are presented as mean ± SEM of N=9 Vehicle vs N=5 IL-6 mice from at least three independent experiments. Two-tailed Unpaired *t* test, *** < p 0.001.

Source data are provided as a Source Data file.

Figure S2

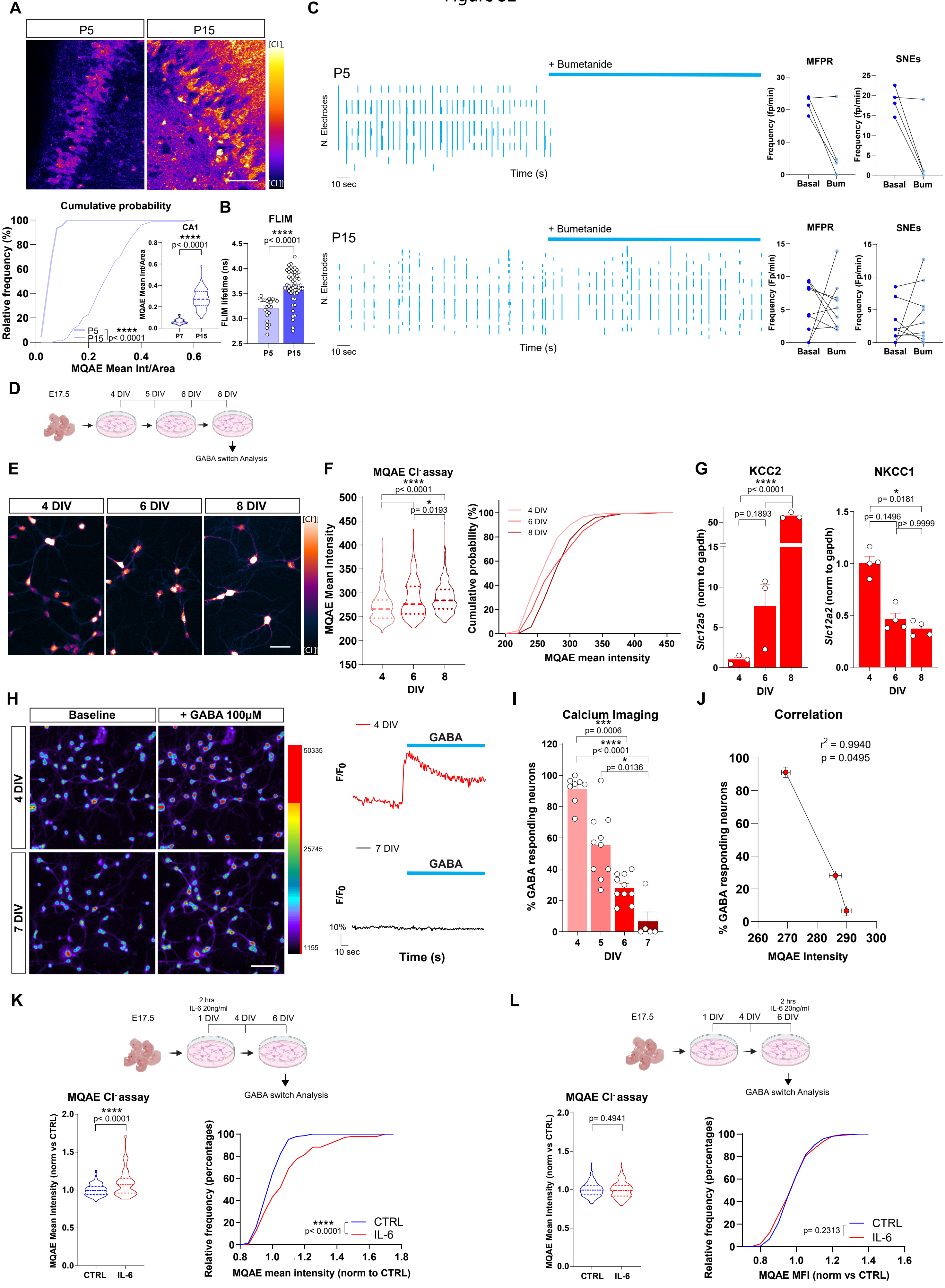

A
P5
P15
[Cl-]i
Cumulative probability
Relative frequency (%)
MQAE Mean Int/Area
CA1
****
p< 0.0001
P5
P15
P7
B
FLIM
FLIM lifetime (ns)
C
+ Bumetanide
N. Electrodes
Time (s)
10 sec
MFPR
SNEs
Frequency (fp/min)
Basal
Bum
D
E17.5
4 DIV
5 DIV
6 DIV
8 DIV
GABA switch Analysis
E
F
MQAE Cl- assay
MQAE Mean Intensity
p= 0.0193
DIV
Cumulative probability (%)
MQAE mean intensity
G
KCC2
NKCC1
p= 0.1893
p= 0.1496
p= 0.0181
p> 0.9999
Slc12a5 (norm to gapdh)
Slc12a2 (norm to gapdh)
H
Baseline
+ GABA 100μM
4 DIV
7 DIV
50335
25745
1155
GABA
F/F0
10%
Time (s)
I
Calcium Imaging
% GABA responding neurons
***
p= 0.0006
p= 0.0136
J
Correlation
r² = 0.9940
p = 0.0495
MQAE Intensity
K
2 hrs
IL-6 20ng/ml
1 DIV
MQAE Mean Intensity (norm vs CTRL)
CTRL
IL-6
Relative frequency (percentages)
MQAE mean intensity (norm to CTRL)
L
p= 0.4941
p= 0.2313
MQAE MFI (norm vs CTRL)

Figure S2. Characterization of GABAergic switch in hippocampal slices and in vitro hippocampal cultures (Related to Figure 2).

A. Representative images (top) and relative quantification (bottom) of MQAE-loaded hippocampal slices showing the different chloride load between the two developmental stages P5 vs P15.

B. Fluorescence Lifetime Intensity Microscopy (FLIM) measurements between P5 and P15 experimental conditions. Data are presented as mean ± SEM of n= 25, 54 analyzed hippocampal neurons. Two- tailed, Unpaired *t* test, $p < 0.0001$.

C. Representative raster plots (left) and relative quantification of MFPR and SNEs (right) of the hippocampal network activity before and after bath application of Bumetanide showing the different response between the two developmental stages P5 (**top**) vs P15 (**bottom**).

D. Schematic cartoon showing the *in vitro* experimental design.

E. Representative images showing MQAE chloride imaging recordings on hippocampal neurons at different developmental stages. Magnification 20X. Scale bar 50 μm.

F. Violin plots (left) and related cumulative distribution plot (right) of MQAE relative fluorescence quantification on hippocampal neurons at different developmental stages. Data are presented as mean ± SEM of n=376, 327, 373 cells from N=3 independent experiments. Kruskal Wallis One Way ANOVA, * $p < 0.05$, **** $p< 0.0001$.

G. Quantification of mRNA relative expression of chloride transporters KCC2, *Slc12a5* (left) and NKCC1, *Slc12a2* (right) in hippocampal neurons across developmental stages. Data are presented as mean ± SEM of three independent experiments. One Way ANOVA, * $p < 0.05$, *** $p< 0.001$.

H. Representative images (left) and traces (right) showing GABA-induced calcium transients, reflecting the GABAergic switch on hippocampal neurons at different developmental stages 4 DIV and 7 DIV. Magnification 20X. Scale bar 50 μm.

I. Histograms from calcium imaging recordings showing the percentages of GABA- responding neurons reflecting the GABAergic switch along development *in vitro*. Data are presented as mean ± SEM of three independent experiments. Kruskal Wallis One Way ANOVA, * $p < 0.05$, *** $p< 0.001$, **** $p< 0.0001$.

J. Graph showing a significant negative correlation between the percentages of GABA- responding neurons and MQAE intensity indicating how small differences in chloride changes determine prominent changes of the calcium transient response elicited by GABA.

K. Schematic cartoon of the experimental *in vitro* paradigm with IL-6 administration at 1 DIV (top), relative MQAE quantification histograms and cumulative distribution analysis at 6 DIV (bottom).

Histograms data are presented as mean ± SEM of n=146 CTRL vs n=103 IL-6-treated neurons from N=2 independent experiments. Unpaired *t* test, **** $p < 0.0001$.

L. Schematic cartoon of the experimental *in vitro* paradigm with IL-6 administration at 6 DIV (top), relative MQAE quantification histograms and cumulative distribution analysis at 6 DIV (bottom). Histograms data are presented as mean ± SEM of n=362 CTRL vs n=362 IL-6-treated neurons from N=3 independent experiments. Unpaired *t* test, ns $p > 0.05$.

Source data are provided as a Source Data file. Panels D, K, L: Created in BioRender. Pozzi, D. (2026) https://BioRender.com/hoa1gtk.

Figure S3

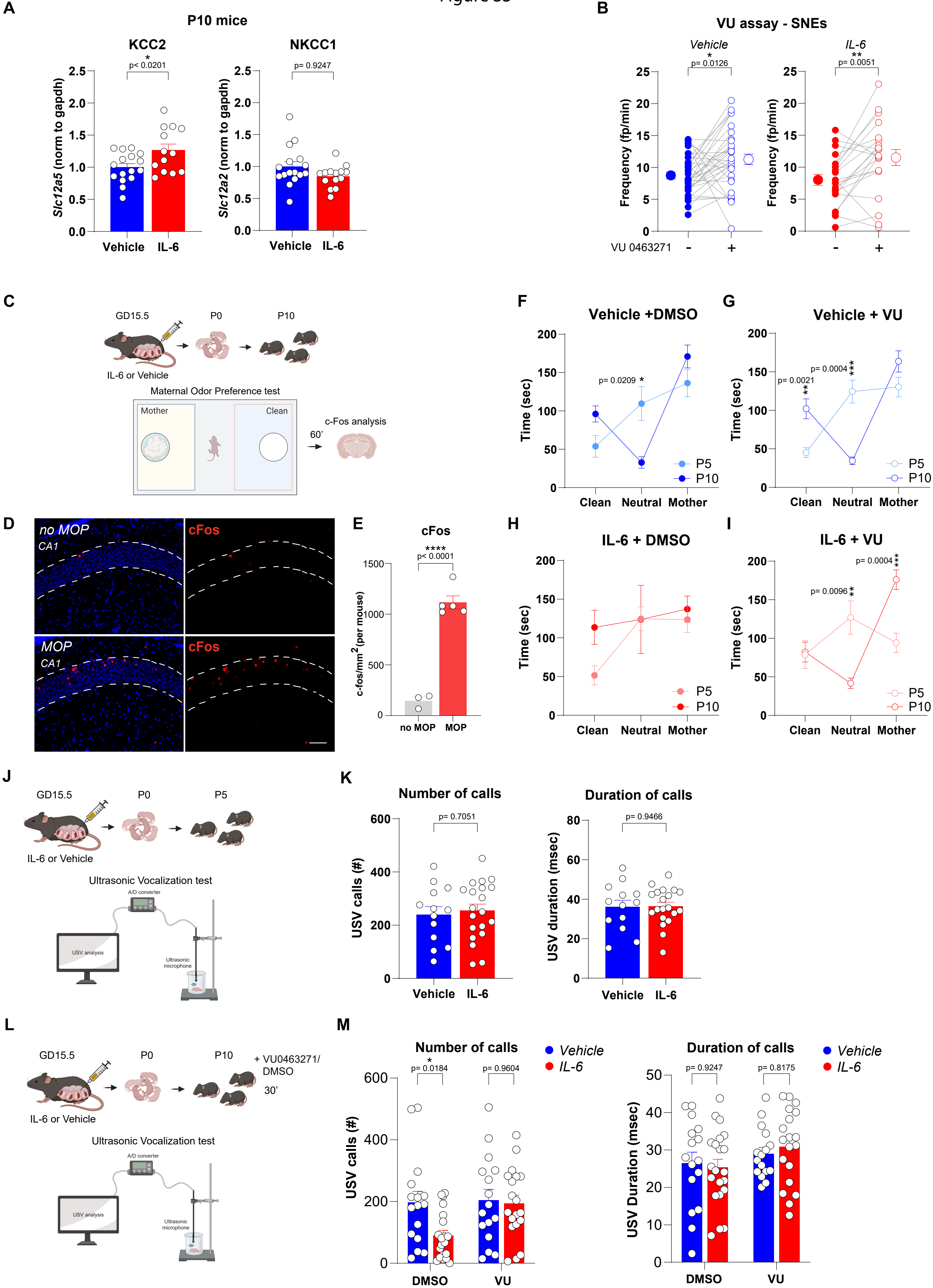
A
P10 mice
KCC2
*
p< 0.0201
Slc12a5 (norm to gapdh)
NKCC1
p= 0.9247
Slc12a2 (norm to gapdh)
Vehicle
IL-6
B
VU assay - SNEs
Vehicle
*
p= 0.0126
IL-6
**
p= 0.0051
Frequency (fp/min)
VU 0463271
C
GD15.5
P0
P10
IL-6 or Vehicle
Maternal Odor Preference test
Mother
Clean
60'
c-Fos analysis
D
no MOP
CA1
cFos
MOP
CA1
cFos
E
cFos
****
p< 0.0001
c-fos/mm2 (per mouse)
no MOP
MOP
F
Vehicle +DMSO
p= 0.0209 *
Time (sec)
P5
P10
Clean
Neutral
Mother
G
Vehicle + VU
p= 0.0004 ***
p= 0.0021 **
H
IL-6 + DMSO
I
IL-6 + VU
p= 0.0004 ***
p= 0.0096 **
J
GD15.5
P0
P5
IL-6 or Vehicle
Ultrasonic Vocalization test
A/D converter
USV analysis
Ultrasonic microphone
K
Number of calls
p= 0.7051
USV calls (#)
Duration of calls
p= 0.9466
USV duration (msec)
Vehicle
IL-6
L
GD15.5
P0
P10
+ VU0463271/ DMSO
30'
IL-6 or Vehicle
Ultrasonic Vocalization test
A/D converter
USV analysis
Ultrasonic microphone
M
Number of calls
*
p= 0.0184
p= 0.9604
USV calls (#)
Vehicle
IL-6
Duration of calls
p= 0.9247
p= 0.8175
USV Duration (msec)
DMSO
VU

Figure S3. Behavioral assessments of Vehicle and IL-6-exposed P5 offspring (Related to Figure 3).

A. Quantification of KCC2 transcript *Slc12a5* (left) and NKCC1 transcript *Slc12a2* (right) mRNA relative expression in the hippocampi of Vehicle and IL-6 exposed P10 mice. Data are presented as mean ± SEM of N=16 Vehicle vs N=13 IL-6 mice from al least three independent experiments. Unpaired *t* test, * < p 0.05.

B. SNEs quantification of Vehicle or IL-6 exposed P5 mice before and after VU0463271 administration. Data are presented as pre- post graph of n=34 Vehicle vs n=24 IL-6 slices from N=5 Vehicle and N=6 IL-6 exposed mice from at least three independent experiments. Paired *t* test, * p < 0.05, ** p < 0.01.

C. Schematic cartoon showing the MOP behavioral test design.

D-E. Representative confocal images (D) and relative quantification (E) of CA1 hippocampal pyramidal neurons from P10 mice exposed or not to MOP, immunostained against neuronal activity marker c-Fos (red). Nuclei counterstained with Hoechst (blue). Magnification 40X. Scale bar 50 µm. Dots represent N=3 noMOP vs N=5 MOP exposed P10 mice. Unpaired *t* test, ****, p < 0.001.

F-I. Time in zone quantification of Maternal Odor Preference test from P5 and P10 mice across the experimental conditions. Vehicle and IL-6 mice administered with either DMSO and VU 30' minutes before the test. Dots represent, for P5 mice, N=10 Vehicle + DMSO vs N=10 Vehicle + VU, N=9 IL-6 + DMSO vs N=10 IL-6 + VU; for P10 mice, N=13 Vehicle + DMSO vs N=16 Vehicle + VU, N=10 IL-6 + DMSO vs N=16 IL-6 + VU; from at least three independent experiments. *Vehicle + DMSO*: Two-Way RM ANOVA (main effect of zone: p < 0.0001;main effect of age: p <0.3354; main effect of subject: p > 0.9999; interaction: p = 0.0024) followed by between-group Ŝidàk's multiple comparison test, *, p < 0.05. *Vehicle + VU*: Two-Way RM ANOVA (main effect of zone: p < 0.0001;main effect of age: p <0.1581; main effect of subject: p > 0.9999; interaction: p < 0.0001) followed by between-group Ŝidàk's multiple comparison test, **, p < 0.01; ***, p < 0.001. *IL-6 + DMSO*: Two-Way RM ANOVA (main effect of zone: p = 0.1828; main effect of age: p = 0.1848; main effect of subject: p = 0.8124; interaction: p = 0.4813) followed by between-group Ŝidàk's multiple comparison test, ns, p > 0.05. *IL-6 + VU*: Two-Way RM ANOVA (main effect of zone: p = 0.0042; main effect of age: p = 0.7755; main effect of subject: p > 0.9999; interaction: p < 0.0001) followed by between-group Ŝidàk's multiple comparison test, **, p < 0.01; ***, p < 0.001.

J. Schematic cartoon summarizing the USV behavioral test design at P5.

K. Ultrasonic vocalization (USV) test. Graphs showing quantification of the total number (left) and duration (right) of the calls vocalized by Vehicle or IL-6 exposed P5 mice. Data are presented as

mean ± SEM of N= 13 Vehicle and N= 20 IL-6-exposed mice from at least three independent experiments. Unpaired *t* test, ns $p > 0.05$.

L. Schematic cartoon summarizing the USV behavioral test design at P10.

M. Ultrasonic vocalization (USV) test. Graphs showing quantification of the total number (left) and duration (right) of the calls vocalized by Vehicle or IL-6 exposed P10 mice with or without VU administration 30 minutes before the test. Data are presented as mean ± SEM of N= 16 Vehicle + DMSO, N=16 Vehicle + VU, N= 20 IL-6 + DMSO, N= 19 IL-6 + VU exposed mice from at least three independent experiments. Number of calls: Two-Way ANOVA (main effect of IL-6: $p = 0.0429$; main effect of drug: $p = 0.0549$; IL-6 x drug interaction: $p = 0.0929$) followed by between- group Ŝidàk's multiple comparison test, *, $p < 0.05$. Duration of calls: Two-Way ANOVA (main effect of IL-6: $p = 0.8743$; main effect of drug: $p = 0.0883$; IL-6 x drug interaction: $p = 0.5169$) followed by between-group Ŝidàk's multiple comparison test, ns, $p > 0.05$.

Source data are provided as a Source Data file. Panels C, J, L: Created in BioRender. Pozzi, D. (2026) https://BioRender.com/hoa1gtk.

Figure S4

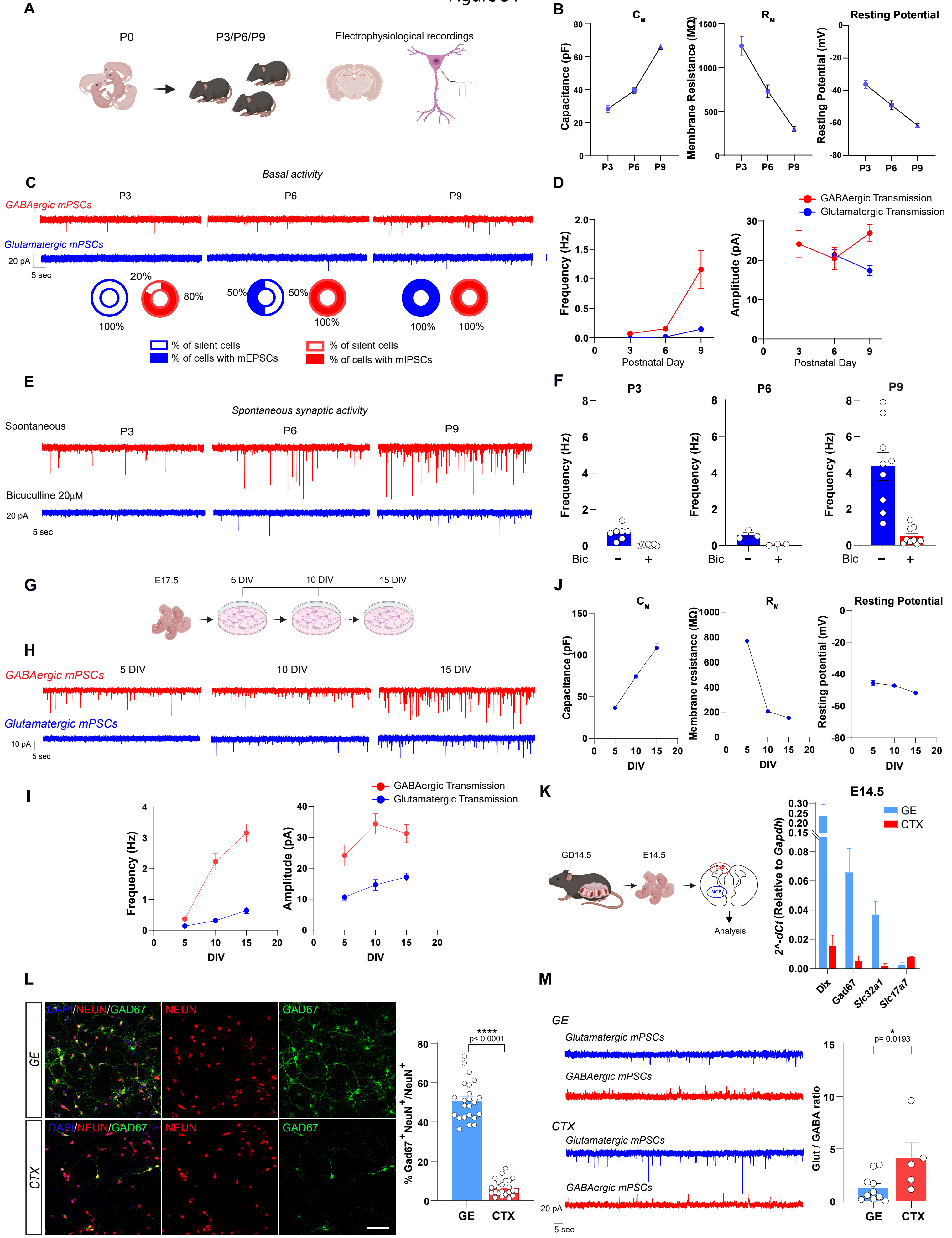

A
P0
P3/P6/P9
Electrophysiological recordings
B
Capacitance (pF)
Membrane Resistance (MΩ)
Resting Potential
Resting Potential (mV)
C
Basal activity
GABAergic mPSCs
Glutamatergic mPSCs
% of silent cells
% of cells with mEPSCs
% of cells with mIPSCs
D
GABAergic Transmission
Glutamatergic Transmission
Frequency (Hz)
Amplitude (pA)
Postnatal Day
E
Spontaneous synaptic activity
Spontaneous
Bicuculline 20μM
F
Frequency (Hz)
Bic
G
E17.5
5 DIV
10 DIV
15 DIV
H
GABAergic mPSCs
Glutamatergic mPSCs
I
Frequency (Hz)
Amplitude (pA)
DIV
J
Capacitance (pF)
Membrane resistance (MΩ)
Resting Potential
Resting potential (mV)
K
E14.5
GD14.5
Analysis
GE
CTX
2^-dCt (Relative to Gapdh)
Dlx
Gad67
Slc32a1
Slc17a7
L
DAPI/NEUN/GAD67
NEUN
GAD67
% Gad67+NeuN+/NeuN+
p< 0.0001
M
GE
CTX
Glutamatergic mPSCs
GABAergic mPSCs
Glut / GABA ratio
p= 0.0193

Figure S4. Prenatal IL-6 elevation enhanced the early GABAergic transmission by directly targeting GABAergic neurons (Related to Figure 4).

A. Schematic cartoon showing the experimental design.

B. Electric passive properties quantifications of CA1 hippocampal neurons recorded at different developmental stages; Capacitance (C_M), Membrane resistance (R_M), and Resting potential. Data are presented as mean ± SEM. P3 n=29, P6 n=42, P9 n=52 recorded neurons from N=3 mice, obtained from three independent experiments.

C. Electrophysiological basal activity recordings showing the arise of GABAergic (red) and Glutamatergic (blue) transmission from P3, P6, P9 mice. Representative traces of GABAergic and Glutamatergic mPSCs recorded in CA1 pyramidal hippocampal neurons in acute brain slices at P3, P6 and P9 mice (top) and relative quantification of the percentages of silent cells and cells displaying either GABAergic and Glutamatergic mPSCs (bottom). 20 pA, 5 sec.

D. Analysis of frequency (left) and amplitude (right) of basal recordings on miniature GABAergic (red) and Glutamatergic (blue) mPSCs at P3, P6, P9 showing that at P3 and P6 the main synaptic inputs consistently detected were the GABAergic basal transmission. Data are presented as mean ± SEM. P3 n=8, P6 n= 13, P9 n= 13 (GABAergic mPSCs) and P3 n=0, P6 n= 6, P9 n= 13 (Glutamatergic mPSCs) recorded neurons from N=3 mice, obtained from three independent experiments.

E. Electrophysiological spontaneous synaptic activity recordings showing the arise of spontaneous activity (red) and its occlusion through Bicuculline administration (blue) on P3, P6, P9 mice. Representative traces of electrical activity recorded in CA1 pyramidal hippocampal neurons in acute brain slices at P3, P6 and P9. Scale bar 20 pA, 5 sec.

F. Histograms showing quantification of frequency of spontaneous electrical activity with of without Bicuculline administration at P3, P6, P9. Data are presented as mean ± SEM. P3 n=7, P6 n=3, P9 n=9 neurons from N=3 mice, obtained from three independent experiments.

G. Schematic cartoon of the experimental *in vitro* design.

H. Electrophysiological basal activity recordings showing the arise of GABAergic (red) and Glutamatergic (blue) transmission at 5, 10, 15 DIV. Representative traces of GABAergic and Glutamatergic mPSCs recorded in hippocampal neurons. 10 pA, 5 sec.

I. Analysis of frequency (left) and amplitude (right) of GABAergic (red) and Glutamatergic (blue) basal synaptic transmission at 5, 10, 15 DIV showing that already at 5 DIV the main synaptic inputs consistently detected were GABAergic mPSCs. Data are presented as mean ± SEM. (Glut, GABA) n= 15,14 at 5 DIV, n= 13, 20 at 10 DIV, n = 16,15 at 15 DIV neurons from N=4 independent experiments.

J. Electric passive properties quantifications of hippocampal neurons recorded at different developmental stages *in vitro*; Capacitance (C_M), Membrane resistance (R_M), and Resting potential. Data are presented as mean ± SEM of n=29, 33, 31 neurons from N=4 independent experiments.

K. Schematic cartoon of GE cultures generation and validation of their identity through qPCR analysis. Assessment of mRNA transcripts of *Dlx*, *Gad67*, *Slc32a1*, *Slc17a7* in GE- or CTX-derived cultures. Two Way ANOVA, followed by Sidak Multiple comparison test.

L. Representative confocal images of GE- and CTX- derived neurons cultured in vitro (left) and relative quantification (right) of the percentage of Gad67+ neurons found in two conditions confirming identity of GE cultures as a pool of GABAergic neurons. Histograms showing the mean ± SEM of n= 23 GE vs n= 23 CTX fields from three independent experiments. Two-tailed Unpaired *t* test, *** p<0.001.

M. Electrophysiological recordings showing the Glutamatergic (blue) and the GABAergic (red) component in 6 DIV GE- of CTX derived neurons. Representative traces (left) and relative Glut/GABA ratio (right) of Glutamatergic and GABAergic mPSCs recorded in GE- or CTX- derived neurons. Histograms data are presented as mean ± SEM of n= 10 GE- and n=5 CTX neuronal cells from independent experiments. Two tailed Mann Whitney Unpaired *t* test, * p < 0.05.

Source data are provided as a Source Data file. Panels A, G, K: Created in BioRender. Pozzi, D. (2026) https://BioRender.com/hoa1gtk.

Figure S5

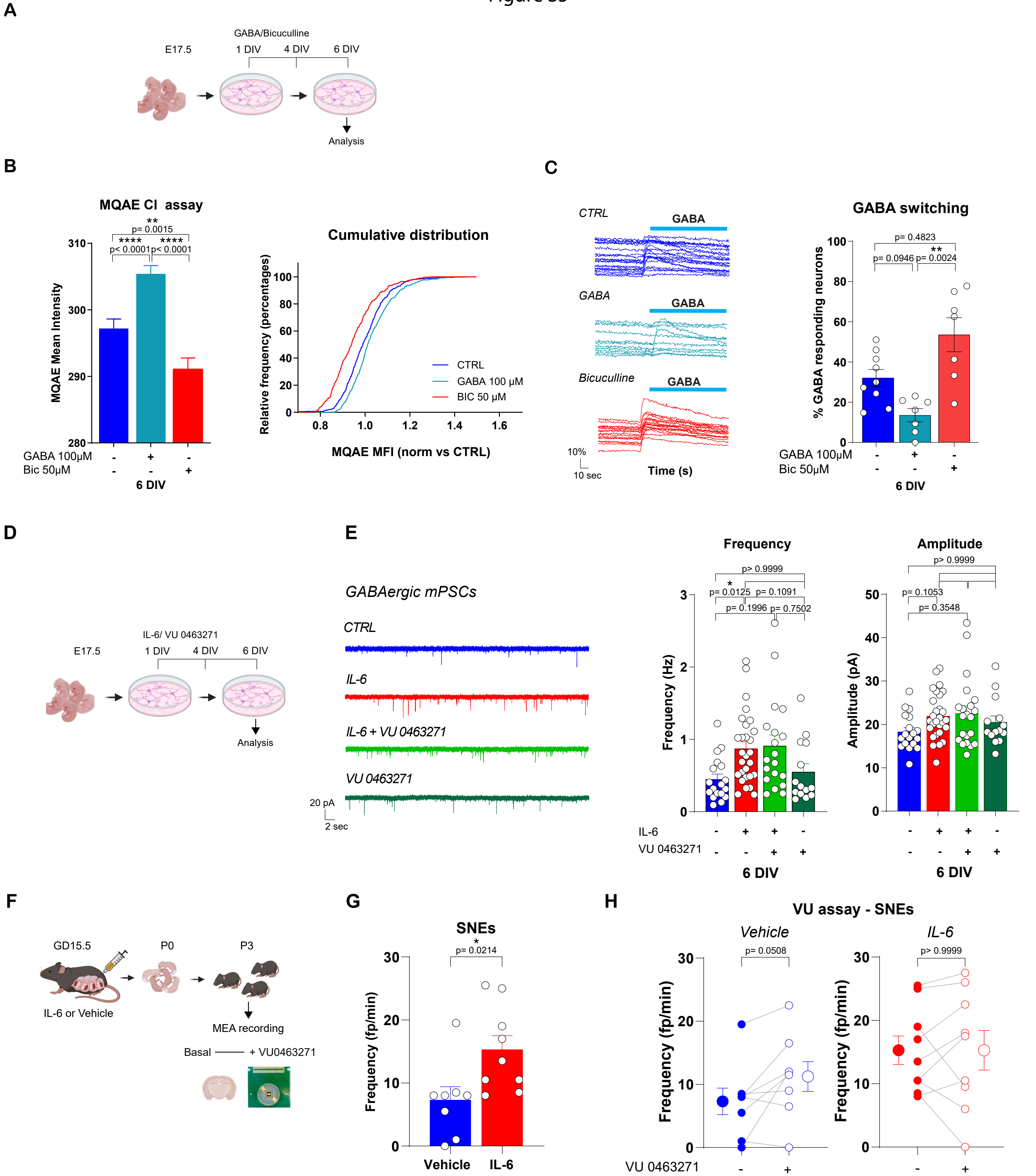

A
GABA/Bicuculline
E17.5
1 DIV
4 DIV
6 DIV
Analysis
B
MQAE Cl assay
**
p= 0.0015
****
****
p< 0.0001
p< 0.0001
MQAE Mean Intensity
310
300
290
280
GABA 100μM
Bic 50μM
6 DIV
Cumulative distribution
Relative frequency (percentages)
100
80
60
40
20
0
0.8
1.0
1.2
1.4
1.6
MQAE MFI (norm vs CTRL)
CTRL
GABA 100 μM
BIC 50 μM
C
CTRL
GABA
Bicuculline
10%
10 sec
Time (s)
GABA switching
% GABA responding neurons
p= 0.4823
**
p= 0.0946
p= 0.0024
100
80
60
40
20
0
GABA 100μM
Bic 50μM
6 DIV
D
IL-6/ VU 0463271
E17.5
1 DIV
4 DIV
6 DIV
Analysis
E
GABAergic mPSCs
CTRL
IL-6
IL-6 + VU 0463271
VU 0463271
20 pA
2 sec
Frequency
p> 0.9999
*
p= 0.0125
p= 0.1091
p= 0.1996
p= 0.7502
Frequency (Hz)
3
2
1
0
IL-6
VU 0463271
6 DIV
Amplitude
p> 0.9999
p= 0.1053
p= 0.3548
Amplitude (pA)
50
40
30
20
10
0
6 DIV
F
GD15.5
P0
P3
IL-6 or Vehicle
MEA recording
Basal
+ VU0463271
G
SNEs
*
p= 0.0214
Frequency (fp/min)
30
20
10
0
Vehicle
IL-6
H
VU assay - SNEs
Vehicle
p= 0.0508
Frequency (fp/min)
IL-6
p> 0.9999
VU 0463271

Figure S5. The IL-6-driven GABAergic transmission enhancement precedes and drives the accelerated GABA developmental switch (Related to Figure 5).

A. Schematic cartoon showing the *in vitro* experimental design.

B. Histograms of MQAE fluorescence quantification (left) and related cumulative distribution analysis (right) on 6 DIV hippocampal neurons showing the MQAE increase or decrease, reflecting the intracellular chloride variation after GABA/ Bicuculline administration. Data are presented as mean ± SEM of n=436 CTRL vs n=481 GABA- vs n=412 Bic-treated neurons from N=2 independent experiments. Kruskal Wallis One Way ANOVA, ** $p < 0.01$, **** $p < 0.0001$.

C. Representative traces of GABA-induced calcium transients (left) and relative quantification of percentages of GABA- responding neurons (right) reflecting the GABAergic switch occurrence after GABA/Bicuculline administration *in vitro*. Data are presented as mean ± SEM of three independent experiments. Kruskal Wallis One Way ANOVA, ** $p< 0.01$.

D. Schematic cartoon showing the *in vitro* experimental design.

E. Representative traces (left) and relative quantification (right) of GABAergic mPSCs recorded in hippocampal neurons across different conditions. Scale bar 20 pA, 2 Sec. Histograms showing frequency and amplitude of n=18 CTRL vs n= 28 IL-6 vs n= 19 VU + IL-6 vs n=14 VU cells from three independent experiments. Data are presented as mean ± SEM. One Way ANOVA, *, $p < 0.05$.

F. Schematic cartoon showing the experimental design.

G. SNEs baseline quantification of either Vehicle or IL-6 exposed P3 mice. Data are presented as mean ± SEM of n=8 Vehicle vs n=9 IL-6 slices from N=3 Vehicle vs N=3 IL-6 exposed mice from three independent experiments. Unpaired *t* test, * $p < 0.05$.

H. SNEs quantification before and after VU0463271 acute administration on either Vehicle or IL-6 exposed P3 mice. Data are presented as pre- post graph of n=8 Vehicle vs n=8 IL-6 slices from N=3 Vehicle vs N=3 IL-6 exposed mice from three independent experiments. Two tailed Paired *t* test, ns $p > 0.05$.

Source data are provided as a Source Data file. Panels A, D, F: Created in BioRender. Pozzi, D. (2026) https://BioRender.com/hoa1gtk.
