# Supplementary material for: Elevated prenatal IL-6 levels disrupt early patterns of hippocampal electrical activity and chloride homeostasis by targeting GABAergic signaling at perinatal stage
Source: Nat Commun. 2026 Oct 15;17:11627. doi: 10.1038/s41467-026-78479-9 (PMC13755421; doi:10.1038/s41467-026-78479-9)
Supplement: Supplementary file 2 [file 41467_2026_78479_MOESM2_ESM.pdf]

nature portfolio

Corresponding author(s): Davide Pozzi

Last updated by author(s): Aug 17, 2026

Reporting Summary

Nature Portfolio wishes to improve the reproducibility of the work that we publish. This form provides structure for consistency and transparency in reporting. For further information on Nature Portfolio policies, see our Editorial Policies and the Editorial Policy Checklist.

Statistics

For all statistical analyses, confirm that the following items are present in the figure legend, table legend, main text, or Methods section.

n/a	Confirmed	
☐	☒	The exact sample size (*n*) for each experimental group/condition, given as a discrete number and unit of measurement
☐	☒	A statement on whether measurements were taken from distinct samples or whether the same sample was measured repeatedly
☐	☒	The statistical test(s) used AND whether they are one- or two-sided<br>*Only common tests should be described solely by name; describe more complex techniques in the Methods section.*
☒	☐	A description of all covariates tested
☐	☒	A description of any assumptions or corrections, such as tests of normality and adjustment for multiple comparisons
☐	☒	A full description of the statistical parameters including central tendency (e.g. means) or other basic estimates (e.g. regression coefficient) AND variation (e.g. standard deviation) or associated estimates of uncertainty (e.g. confidence intervals)
☐	☒	For null hypothesis testing, the test statistic (e.g. *F*, *t*, *r*) with confidence intervals, effect sizes, degrees of freedom and *P* value noted<br>*Give P values as exact values whenever suitable.*
☒	☐	For Bayesian analysis, information on the choice of priors and Markov chain Monte Carlo settings
☒	☐	For hierarchical and complex designs, identification of the appropriate level for tests and full reporting of outcomes
☒	☐	Estimates of effect sizes (e.g. Cohen's *d*, Pearson's *r*), indicating how they were calculated

Our web collection on statistics for biologists contains articles on many of the points above.

Software and code

Policy information about availability of computer code

Data collection	pClamp Software (Axon Instruments); Metaphluor (Molecular Device); LAS X SP8II, Stellaris8 microcope software (Leica); BrainWave 4 (3Brain); ImageJ (Fiji); EXcellence RT software (Olympus); Applied Biosystems® ViiA™ 7 Real-Time PCR System (Applied Biosystems).
Data analysis	Clampfit software (Molecular Devices); BrainWave 5 (3Brain); GraphPad PRISM Version 11.0 (GraphPad); Phython 3.11; Origin 8 (OriginLab)

For manuscripts utilizing custom algorithms or software that are central to the research but not yet described in published literature, software must be made available to editors and reviewers. We strongly encourage code deposition in a community repository (e.g. GitHub). See the Nature Portfolio guidelines for submitting code & software for further information.

Data

Policy information about availability of data

All manuscripts must include a data availability statement. This statement should provide the following information, where applicable:

- Accession codes, unique identifiers, or web links for publicly available datasets
- A description of any restrictions on data availability
- For clinical datasets or third party data, please ensure that the statement adheres to our policy

The manuscript includes a Data Availability statement providing information on the availability of the data generated in this study. All data supporting the findings of this study are provided in the Source Data file accompanying this paper. The manuscript also includes a Code Availability statement providing information on the availability and accessibility of the custom code used in this study.

Research involving human participants, their data, or biological material

Policy information about studies with human participants or human data. See also policy information about sex, gender (identity/presentation), and sexual orientation and race, ethnicity and racism.

Reporting on sex and gender	N/A
Reporting on race, ethnicity, or other socially relevant groupings	N/A
Population characteristics	N/A
Recruitment	N/A
Ethics oversight	N/A

Note that full information on the approval of the study protocol must also be provided in the manuscript.

Field-specific reporting

Please select the one below that is the best fit for your research. If you are not sure, read the appropriate sections before making your selection.

☒ Life sciences ☐ Behavioural & social sciences ☐ Ecological, evolutionary & environmental sciences

For a reference copy of the document with all sections, see nature.com/documents/nr-reporting-summary-flat.pdf

Life sciences study design

All studies must disclose on these points even when the disclosure is negative.

Sample size	The sample size was not formally predetermined using a statistical power calculation, but was instead based on prior experience with similar experimental settings and on previously published data. The chosen sample size was considered sufficient to ensure reproducibility and to detect meaningful effects.
Data exclusions	Potential outliers were identified using GraphPad Software with the ROUT method. A threshold of Q = 1% was applied for electrophysiological, molecular and biochemical assays, whereas a threshold of Q = 10% was selected for behavioral assays to account for the inherently higher variability characteristic of behavioral data.
Replication	Experiments were performed in independent replicates to assess reproducibility. In each replicate, results were carefully evaluated to ensure consistency across replicates. All experiments presented in this study yielded consistent outcomes, confirming the reliability of the findings and supporting the robustness of the data
Randomization	Randomization was not applicable to this study, as animals were assigned to experimental conditions based on treatment (vehicle vs IL-6) and all experiments were conducted under standardized and controlled conditions to minimize potential sources of bias.
Blinding	Behavioural experiments were performed in a blinded manner, with experimenters unaware of the treatment conditions during data collection and analysis.

Reporting for specific materials, systems and methods

We require information from authors about some types of materials, experimental systems and methods used in many studies. Here, indicate whether each material, system or method listed is relevant to your study. If you are not sure if a list item applies to your research, read the appropriate section before selecting a response.

Materials & experimental systems

n/a	Involved in the study	
☐	☒	Antibodies
☒	☐	Eukaryotic cell lines
☒	☐	Palaeontology and archaeology
☐	☒	Animals and other organisms
☒	☐	Clinical data
☒	☐	Dual use research of concern
☒	☐	Plants

Methods

n/a	Involved in the study	
☒	☐	ChIP-seq
☐	☒	Flow cytometry
☒	☐	MRI-based neuroimaging

Antibodies

Antibodies used	The following primary antibodies were used in this study:<br>For Western Blotting: rabbit anti-pSTAT3 Tyr705 (Cell Signaling Technology, #D3A7), rabbit anti-STAT3 (Cell Signaling Technology, #D3Z2G), rabbit anti-GAPDH (Synaptic Systems, #247002).<br>For IF: rabbit anti-KCC2 (Millipore, 1:500, #07-432), guinea pig anti-NeuN (Abcam, 1:500, #ab134014), mouse anti-β3-tubulin (Thermo Fisher Scientific, 1:500, #MA1-118), guinea pig anti-MAP2 (Synaptic Systems, 1:1000, #188 004), rabbit anti-GABA (Thermo Fisher Scientific, 1:1000, # PA5-32241), and rabbit anti-c-fos (Synaptic Systems, 1:1000, #226003). Appropriate species-specific secondary antibodies conjugated to Alexa Fluor dyes (Thermo Fisher Scientific) or HRP-conjugated secondary antibodies (Thermo Fisher Scientific) were used according to the manufacturer's instructions.<br>For FACS analysis: CCR2-BV421 (clone SA203G11, Biolegend, cat#150605), F4/80-AlexaFlour488 (clone T45- 2342, BD, cat#567201), Ly6C APC-Cy7(clone AL-21, BD, cat#560596), CD49b-BV711 (clone HMa2, BD, cat#740704), Ly6G-BUV563 (clone 1A8, BD, cat#612921), MHCII-BUV737 (clone2G9, BD, cat#748708), CD3-PE-Cy7 (clone 145-2C11, Invitrogen, cat#25-0031-82), CD19-BUV661 (clone 1D3, BD, cat#612971), CD45-BV605 (clone 30-F11, Biolegend, cat#103155), CD11b-BUV395 (M1/70, BD, cat#563553), CD11c-BV786 (clone HL3, BD, cat#563735).
Validation	All primary antibodies used in this study were validated by the manufacturers for the relevant applications and species, as indicated in the respective datasheets. Antibody specificity was further supported by the consistency of the observed staining patterns with known cellular and subcellular localization, as well as by agreement with previously published studies (For instance see Mirabella et al 2021)

Animals and other research organisms

Policy information about studies involving animals; ARRIVE guidelines recommended for reporting animal research, and Sex and Gender in Research

Laboratory animals	All experiments were performed using C57BL/6N mice (Charles River Laboratories). Pregnant females aged 3–6 months were used, and embryos (E15-E18) and offspring at defined postnatal stages were selected for in vivo and ex vivo analyses. Animals were housed under controlled conditions with food and water available ad libitum
Wild animals	This study did not involve wild animals
Reporting on sex	Only male offspring were used for the in vivo experiments, whereas embryonic sex was not determined for the in vitro experiments; therefore, primary neuronal cultures were derived from mixed-sex embryos. Sex was therefore not included as a biological variable in the experimental design.
Field-collected samples	This study did not involve samples collected from the field.
Ethics oversight	All animal procedures were conducted in accordance with the guidelines of the European Community Council (Directive 2010/63/EU) and were approved by the Institutional Animal Care and Use Committee (IACUC) of the Humanitas Research Hospital and by the Italian Ministry of Health (permission numbers 467 and 565).

Note that full information on the approval of the study protocol must also be provided in the manuscript.

Plants

Seed stocks	N/A
Novel plant genotypes	N/A
Authentication	N/A

Flow Cytometry

Plots

Confirm that:

☒ The axis labels state the marker and fluorochrome used (e.g. CD4-FITC).

☒ The axis scales are clearly visible. Include numbers along axes only for bottom left plot of group (a 'group' is an analysis of identical markers).

☒ All plots are contour plots with outliers or pseudocolor plots.

☒ A numerical value for number of cells or percentage (with statistics) is provided.

Methodology

Sample preparation	Single-cell suspensions were obtained from brain and spleen tissues of P5–P6 mice. Tissues were mechanically dissociated through 70 µm cell strainers in PBS to obtain a homogeneous cell suspension. Cells were then stained with a fixable viability dye prior to antibody labeling.
Instrument	Flow cytometry data were acquired using a standard flow cytometer. Instrument settings were optimized for each experiment according to the manufacturer's recommendations.
Software	Flow cytometry data were analyzed using standard flow cytometry analysis software. Gating and quantification were performed consistently across all samples.
Cell population abundance	The abundance of immune cell populations was determined as the percentage of total live cells within each sample. Quantification of innate and adaptive immune cell subsets was performed using established markers, and comparisons between experimental groups were carried out under identical gating conditions.
Gating strategy	Cells were first gated based on forward and side scatter (FSC/SSC) parameters to exclude debris, followed by doublet discrimination and selection of live cells using a viability dye. Immune cell populations were then identified based on specific marker expression (including F4/80, CD3, and CD19). Gating thresholds for positive and negative populations were defined based on appropriate controls and applied consistently across all samples.

☒ Tick this box to confirm that a figure exemplifying the gating strategy is provided in the Supplementary Information.
