# Supplementary material for: Elevated prenatal IL-6 levels disrupt early patterns of hippocampal electrical activity and chloride homeostasis by targeting GABAergic signaling at perinatal stage
Source: Nat Commun. 2026 Oct 15;17:11627. doi: 10.1038/s41467-026-78479-9 (PMC13755421; doi:10.1038/s41467-026-78479-9)
Supplement: Supplementary file 3 [file 41467_2026_78479_MOESM3_ESM.pdf]

nature portfolio

# Peer Review File

# Elevated prenatal IL-6 levels disrupt early patterns of hippocampal electrical activity and chloride homeostasis by targeting GABAergic signaling at perinatal stage

Corresponding Author: Dr Davide Pozzi

**This file contains all reviewer reports in order by version, followed by all author rebuttals in order by version.**

Version 0:

Reviewer comments:

Reviewer #1

(Remarks to the Author)
The manuscript by Desiato et al. investigates the effects of maternal immune activation on hippocampal circuit development . Their data suggest that maternal elevation of IL-6 disrupts chloride homeostasis and causes a premature shift in the GABA-A reversal potential, via STAT-3 signaling and precocious expression of KCC2. While the results are interesting, the functional data aiming to correlate the IL-6 dependent changes in expression of KCC2 and GABAergic markers to the network activity are not convincing.

1. The authors claim that prenatal IL-6 treatment disrupts early hippocampal network activity, yet the frequency of the synchronous network events (SNEs) is not different between the control vs IL-6 groups at any developmental stage studied in Figure 1. The small decrease in the frequency of LFPs is not sufficient to support that network activity is altered, as these events could reflect activity of individual neurons that are positioned on top or close to the MEA electrodes.

2. It is not clear why the authors decided to study the excitability of principal neurons in area CA1 rather than in the CA3, where the network activity in the hippocampus is typically generated.

3. When comparing between age groups, the MQAE fluorescence should be calibrated at the end of the experiments, or the results should be confirmed with recordings of GABA-A reversal potential.

4. Why the basal frequency of LFPs in Figure 3L is not different between the groups (control vs Il-6) and is two times higher as compared to corresponding data in Figure 1B ? Also, please indicate if the raster plots represent activity before or after drug application.

5. It is not clear why in figure 4I-K, STAT3 phosphorylation levels are assessed instead of expression levels. Is STAT3 phosphorylation increased in IL6 treated mice?

6. Figure S4 the image quality (example traces) is not sufficient. For the data in figure S4M, please show expanded traces of the individual events, to illustrate the detection criteria for the low-amplitude events vs noise. Please also include the details used for event detection in the methods.

7. In figure 5, the authors show that treatment of hippocampal cultures with bicuculline prevents the IL-6 -induced increase in KCC2 expression. However, bicuculline treatment results in homeostatic plasticity, involving a variety of compensatory changes that could indirectly influence [Cl-]i and KCC2 levels. To argue for causal relationship between early GABAergic activity and upregulation of KCC2 expression (Fig 5), the authors should use treatments that do not fully block GABAergic activity, but reduce it to control levels

8. Discussion (e.g. paragraph starting from row 339) contains several statements that are not supported by the manuscript data and without references to previous publications.

9. Discussion line 372 : The manuscript does not contain data to show that the enhancement of the GABAergic transmission

elicited by IL-6 precedes the GABA switch in acute brain slices. The mIPSC data in acute slices are from P5.

10. Minor: in all figures containing MEA recordings, please indicate which hippocampal subfield the data in the raster plots represents

Reviewer #2

(Remarks to the Author)
The study “Elevated prenatal IL-6 levels disrupt early patterns of hippocampal electrical activity 2 by targeting GABAergic signaling at perinatal stage” by Desiato et al. was evaluated. The work demonstrates that a transient prenatal elevation of interleukin-6 (IL-6) at gestational day 15.5 significantly alters the normal maturation of hippocampal network activity in newborn mice by accelerating the developmental switch of GABAergic signaling. Using high-density multi-electrode array recordings, the authors found that at postnatal day 5 (P5), mice prenatally exposed to IL-6 exhibited significantly reduced hippocampal spiking activity compared to controls, despite unchanged network synchrony and intrinsic neuronal excitability. This disruption was not due to early inflammation, as immune profiling and cytokine gene expression analyses showed no signs of local or systemic immune activation.
Instead, IL-6 induced a premature reduction in intracellular chloride concentration in CA1 pyramidal neurons by upregulating the potassium-chloride cotransporter KCC2 via STAT3-dependent transcriptional mechanisms. This led to a premature shift in GABA signaling from excitatory to inhibitory. Critically, IL-6 was shown to directly enhance early GABAergic synaptic activity by acting on GABAergic neurons, again through STAT3 signaling. This enhancement occurred before KCC2 upregulation and was necessary for the developmental switch in GABA polarity. Blocking GABAergic activity prevented IL-6-induced KCC2 expression and chloride reduction, establishing a causal link. Finally, this altered signaling cascade disrupted early behavioral responses in IL-6-exposed mice when KCC2 was pharmacologically inhibited, further confirming its functional relevance.

Novelty/originality
The work describes new results, the data obtained are significant and highly original. The paper uniquely demonstrates that transient prenatal IL-6 elevation directly alters early hippocampal network development by enhancing GABAergic transmission and accelerating the GABA polarity switch via STAT3–KCC2 signaling, without inflammation. This mechanistic clarity and early developmental timing clearly distinguish it from prior MIA studies (e.g., Smith et al., 2007; Shin Yim et al., 2017).

The work supports its conclusions with well-controlled experiments across mainly in ex vivo models, using some in vivo experiments. It uses multiple techniques (HD-MEA, patch-clamp, imaging, gene expression) and causal interventions (IL-6, STAT3 inhibitors, KCC2 blockade) to establish mechanistic links. While longitudinal behavioral data would strengthen clinical relevance, the current evidence supports the main mechanisms, although the main causal points should be tested in vivo as well.

Methodology is generally sound and employs well-established techniques to assess neural network activity and chloride homeostasis. There is sufficient detail for reproducibility, including experimental time points (e.g., P3, P5), IL-6 dosing, cell culture timelines, and the use of specific inhibitors (e.g., Stattic, VU0463271).

In general the study is strong, but heavily relies on ex vivo models (slices and cultures). It would make the material much stronger, if a few more in vivo experiments would be added.

There are a few specific points to address:

1. It would be good to confirm the mechanisms with more robust in vivo experiments. (Stattic or Gal interventions in vivo for example, or an elegant 4-group experiment – ctrl+vehicle, ctrl+KCC2 inhibitor, IL-6 + vehicle, IL-6 + KCC2 inhibitor)
2. The behavioral assessments are limited to early postnatal stages; long-term consequences of the early IL-6-induced changes are inferred but not directly tested. It would be good to see a longer-term effect.
3. The maternal odor preference test is modest in scope—more diverse behavioral assays would strengthen conclusions about functional impact.
4. Neuronal networks (connectivity, synapse density, neuronal morphology, etc..) should be assessed in vivo at later postnatal timepoints.
5. While GABAergic cultures are used, in vivo confirmation of IL-6's selective action on GABAergic neurons (e.g., via conditional STAT3 knockouts, central pharmacological inhibition) would improve mechanistic precision.
6. It would be good to see the developmental window of IL-6 sensitivity. For example, injecting IL-6 directly into pups at P1–P3 and assessing hippocampal network activity and KCC2 expression at P5 would confirm whether the mechanism is time-sensitive.
7. It would also be good to strengthen the temporal relationship between IL-6, GABA-related gene expression, and KCC2. By collecting hippocampal tissue from control and IL-6-exposed pups at P3, P5 and P10, and measuring expression of Gad67, Slc32a1, Kcc2, and Stat3 over time, the Authors could confirm sequential expression pattern.

Reviewer #3

(Remarks to the Author)

Desiato, Pozzi, and colleagues describe, in the manuscript titled “Elevated prenatal IL-6 levels disrupt early patterns of hippocampal electrical activity by targeting GABAergic signaling at perinatal stage”, the mechanisms by which cytokine interleukin-6 (IL-6) influences early brain development. Using a variety of method including IL-6 injection to pregnant dams at gestation day 15.5 and IL-6 application to neuronal culture with those derived from postnatal days of embryos coupled with electrophysiology and hitochemical analyses, the authors show that IL-6 influences GABAergic transmission, leading to the acceleration of the GABA developmental switch and reduction in neural activity, which culminates in abnormal maturation of offspring’s hippocampal circuitry. This work added to a growing number of works highlighting the modulatory roles of maternally derived immune molecules in developing neuronal circuits. The strength of this manuscript lies in the elucidation at the molecular level of the effects of IL-6 on the premature developmental switch in GABAergic signaling. Through extensive analyses, the authors, for example, showed that IL-6 treatments lead to premature lowering of chloride ion in neurons and changed expression of various GABA-related genes.

Overall, this is an important work providing novel ideas to the neuroimmunology field in understanding how cytokines influence brain development. I am in support of the publication of this work in essence, but wanted to make the following comments.
While the authors showed that STAT3 gene expression is increased in neuronal culture following IL-6 treatments, it is not clear whether the STAT3 level is changed in the hippocampal neurons of the affected offspring. In other words, while the authors alluded that IL-6 directly affects GABA-signaling in the brain, they haven’t proved this hypothesis. For example, earlier reports have shown that IL-17 in the mother and CCL2 in the offspring may play roles downstream of IL-6. Is it possible that IL-6 mediates the effects through the modulation of other effector molecules, such as IL-17 and CCL2? While it is desirable to test the effects of these immune molecules in influencing GABA-related circuits, the authors at least need to clarify these points in the discussion.

Also, what are the long-lasting effects of IL-6 treatments? Did the authors detect changes in the neural activity or GABA-related gene expression in the brains of MIA adult mice?
While the authors stated that USV phenotypes are not different in P5 control and IL-6 mice, many earlier studies by other groups have reported that maternal immune activation produced significant changes in USV calls. The authors need to clarify why they have seen different results. Is it because they have measured USV calls at earlier time points (P5), while many others did that at later P7-P9?

Version 1:

Reviewer comments:

Reviewer #1

(Remarks to the Author)
While the authors have now addressed most of the concerns raised, several issues related to MEA recordings still need clarification

1. The methods lack details on how the reported MFPR values are obtained (how many electrodes/area were analysed per recording, were the frequency counts from all electrodes averaged etc).

2. The authors indicate in the methods that ‘At the end of each recording session, a high-definition (HD) image of the slice was taken using HD digital microscope (Bysameyee) and subsequently superimposed to the activity map ensuring a precise electrical-anatomical correlation’. Thus, it should be possible to find in which hippocampal subfield the electrodes were located. This is important, because CA3 typically is more active compared to CA1, and the localization of the electrodes selected for analysis can make a difference.

3. The manuscript remains confusing in terms of terminology related to network activity. The authors conclude in the results ‘…IL-6 exposure reduces hippocampal spiking activity at P5, without affecting the synchrony of the network activity.’ However, throughout the text they refer to ‘altered hippocampal network activity’, or in some cases ‘spiking activity’ ; the terminology should be consistent throughout.

4. In the revised text, the authors now refer to the intensity and duration of the LFP discharge as a measure of the strength of synaptic signaling, but there is no data on LFP amplitude or duration in the manuscript. Was this parameter affected by the treatments?

5. Minor : Original comment #4 required the condition (baseline vs drug) to be indicated in the raster plots of the figure 3, the labeling of the insets was ok already in the original version.

6. Minor: The quality of the traces in the figure S4 should be checked (this could be an issue with the PDF conversion)

Reviewer #2

(Remarks to the Author)

The authors have substantially strengthened the manuscript and addressed most of my major concerns through meaningful new experiments and clearer mechanistic framing. Most importantly, they now provide a much-improved developmental sequence showing that enhanced GABAergic transmission is already present at P3, preceding KCC2 upregulation at P5 and later behavioral abnormalities at P10. This temporal refinement significantly improves the central mechanistic argument. The addition of new in vivo experiments, particularly the P10 behavoiral analyses (MOP and USV) together with pharmacological rescue by KCC2 inhibition, represents a substantial improvement over the original submission and provides stronger functional relevance. The new picrotoxin intervention further supports the proposed causal relationship between early GABAergic activity and later KCC2 dysregulation in vivo. These additions move the work beyond its previous heavy dependence on ex vivo systems.

That said, some limitations remain. My original request for more definitive in vivo mechanistic precision – particularly direct cell-type-specific validation of STAT3/GABAergic neuron involvement – has only been partially addressed. While the authors provide reasonable technical explanations for why conditional or selective in vivo approaches were not feasible, this remains an important unresolved limitation. Similarly, long-term circuit and behavioral consequences are better contextualized, but much of this still relies on prior published work rather than direct incorporation into the current manuscript.

Overall, however, the revision is substantially improved, more mechanistically coherent, and considerably stronger than the initial submission. Although not every point has been fully resolved at the highest mechanistic level, the authors have addressed the core concerns sufficiently for publication, provided that remaining limitations are clearly acknowledged in the discussion.

I therefore recommend acceptance after minor revision. Specifically, the authors should explicitly temper their claims regarding direct in vivo cellular specificity and clearly acknowledge that STAT3/GABAergic neuron-selective causality remains to be demonstrated. They should also ensure that the dicsussion more clearly distinguishes between findings directly established in the current manuscript and conclusions inferred from prior published work, particularly regarding long-term outcomes. Finally, a brief clarification of these remaining mechanistic limitations would improve interpretive balance without requiring substantial additional experimentation.

Reviewer 1’s technical concerns regarding electrophysiology, chloride measurements, and developmental timing appear to have been addressed thoroughly with substantial new controls and experiments. Reviewer 3’s broader questions regarding downstream immune mediators and behavioral interpretation were also addressed reasonably well, though – as with my comments – some mechanistic specificity remains inferential rather than definitive.

Reviewer #3

(Remarks to the Author)
The authors addressed all the critiques I raised. I no longer have any comments.

Version 2:

Reviewer comments:

Reviewer #1

(Remarks to the Author)
The revision has improved the accuracy and clarity of the manuscript. I have no further comments.

Reviewer #2

(Remarks to the Author)
I have no further comments, the Authors have addressed all my points.

**Open Access** This Peer Review File is licensed under a Creative Commons Attribution 4.0 International License, which permits use, sharing, adaptation, distribution and reproduction in any medium or format, as long as you give appropriate credit to the original author(s) and the source, provide a link to the Creative Commons license, and indicate if changes were made.
In cases where reviewers are anonymous, credit should be given to 'Anonymous Referee' and the source.
The images or other third party material in this Peer Review File are included in the article's Creative Commons license, unless indicated otherwise in a credit line to the material. If material is not included in the article's Creative Commons license and your intended use is not permitted by statutory regulation or exceeds the permitted use, you will need to obtain permission directly from the copyright holder.
To view a copy of this license, visit https://creativecommons.org/licenses/by/4.0/

## Point to point rebuttal letter

Reviewers' comments:

**Reviewer #1 (Remarks to the Author):**

The manuscript by Desiato et al. investigates the effects of maternal immune activation on hippocampal circuit development. Their data suggest that maternal elevation of IL-6 disrupts chloride homeostasis and causes a premature shift in the GABA-A reversal potential, via STAT-3 signaling and precocious expression of KCC2. While the results are interesting, the functional data aiming to correlate the IL-6 dependent changes in expression of KCC2 and GABAergic markers to the network activity are not convincing.

1. The authors claim that prenatal IL-6 treatment disrupts early hippocampal network activity, yet the frequency of the synchronous network events (SNEs) is not different between the control vs IL-6 groups at any developmental stage studied in Figure 1. The small decrease in the frequency of LFPs is not sufficient to support that network activity is altered, as these events could reflect activity of individual neurons that are positioned on top or close to the MEA electrodes.

**We thank the reviewer for raising this point, which allows us to better elucidate this aspect. As described in the discussion, LFPs and SNEs measure different aspects of network activity: while the occurrence of SNEs reflects the spontaneous tendency of neurons to fire action potentials, LFPs instead reflect the strength of synaptic excitatory signaling, in terms of intensity and duration of the discharge. As a consequence, from a mechanistic point of view, SNEs could be due to some form of peacemaker activity of the system, whilst LFPs depend on the strength of synaptic activity engaged. Hence, any change in synaptic activity across the network might be detectable as a modulation of LFPs even without a significant change in the SNEs [1-3]. Since LFPs are influenced by both excitatory and inhibitory postsynaptic potentials [4], even a slight shift in their balance (as shown in the present work), can alter the LFP's amplitude, shape, or frequency without significantly affecting SNEs.**

**With regard to the statement, "as these events could reflect activity of individual neurons that are positioned on top or close to the MEA electrodes", we respectfully believe that this interpretation is unlikely for the reasons outlined below:**

1) **The data shown in Figure 1b (corresponding to P5) derive from the analysis of multiple biological replicates (n = 11 Vehicle and n = 14 IL-6 slices, from N = 5 Vehicle and N = 5 IL-6 mice). Given this sample size and the number of independent animals, it is highly unlikely that, across all experiments and replicate, only neurons in IL-6 slices positioned directly on or in close proximity to the MEA electrodes would systematically exhibit a reduced firing rate.**
2) **The coupling between electrodes and slices was systematically assessed and quantified in both Vehicle and IL-6 groups across all developmental stages, in order to monitor recording quality and reliability. As shown in the Figure R1.1, the number of active electrodes was robust and remained stable across all experimental conditions, with no detectable differences between ages or treatment groups. These observations indicate that variations in electrode–slice coupling are unlikely to account for the observed effects. In addition, any electrode that did not meet the 200 µV signal threshold were automatically excluded from the analysis. This exclusion procedure did not alter the mean firing rate per electrode (MFPR), ensuring that calculated values reflect activity recorded from reliably coupled electrodes only. These considerations prompted us to include the Figure R1 in the revised version of the manuscript together with a clarifying statement noting that the results were not influenced by differences in electrode–slice coupling.**

**Taken together, these considerations strongly support the conclusion that the observed reduction in LFP activity in IL-6 slices is not attributable to electrode positioning or electrode-tissue coupling, but rather reflects a robust and biologically meaningful phenomenon. While we appreciate the reviewer's concern,**

**we believe that our technical approach supports the robustness of our recordings and the scientific validity of the reported findings.**

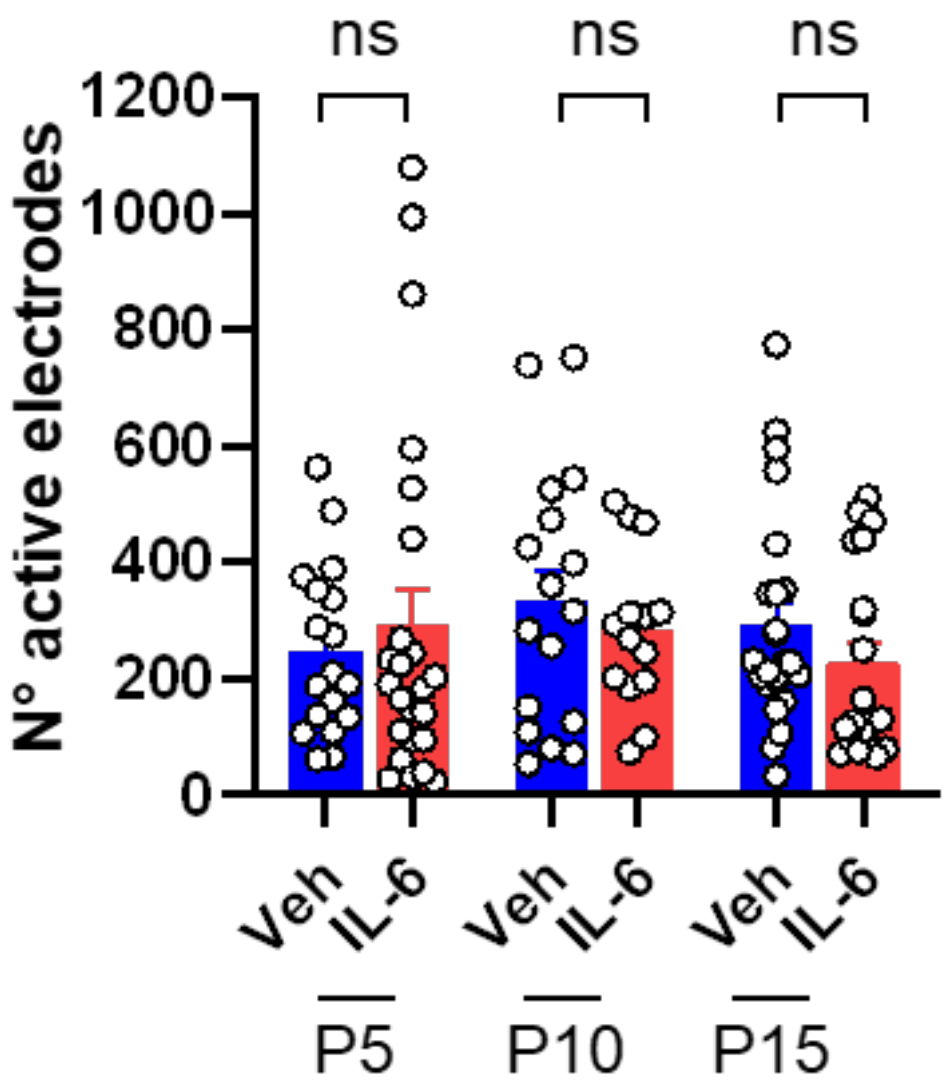


**Figure R1.1.** *Histograms showing the number of active electrodes across the experimental conditions (Vehicle vs IL-6) at different postnatal stages (P5, P10, P15). Dots represent n= 18, 17, 24 vs n= 24, 14, 20 slices from N=5, 4, 4 Vehicle and N=5, 4, 4 IL-6 exposed mice. Data are presented as mean ± SEM. One-way ANOVA, ns > 0.05.*

2. It is not clear why the authors decided to study the excitability of principal neurons in area CA1 rather than in the CA3, where the network activity in the hippocampus is typically generated

**Although CA3 was originally recognized as the main hippocampal region where network activity is generated [5,6], other studies have highlighted that the CA1 region (i) is a primary source of recurrent excitation [7] and (ii) can generate GDPs even when disconnected from CA3 [2,8]. Moreover, CA1 has been implicated in social memory [9] and has been reported to be altered in several NDD models [10,11], including MIA models [12,13]. In addition, we previously showed that prenatal IL-6 elevation leads to hippocampal hyperconnectivity associated with synaptic abnormalities in the CA1 region [14]. While these considerations support our focus on CA1, as suggested by the reviewer we also examined excitability in the CA3 region, and found no changes in the two conditions Figure R1.2, consistent with the results obtained in CA1. This new data has been included into the revised version of the manuscript (See figure 1 revised).**

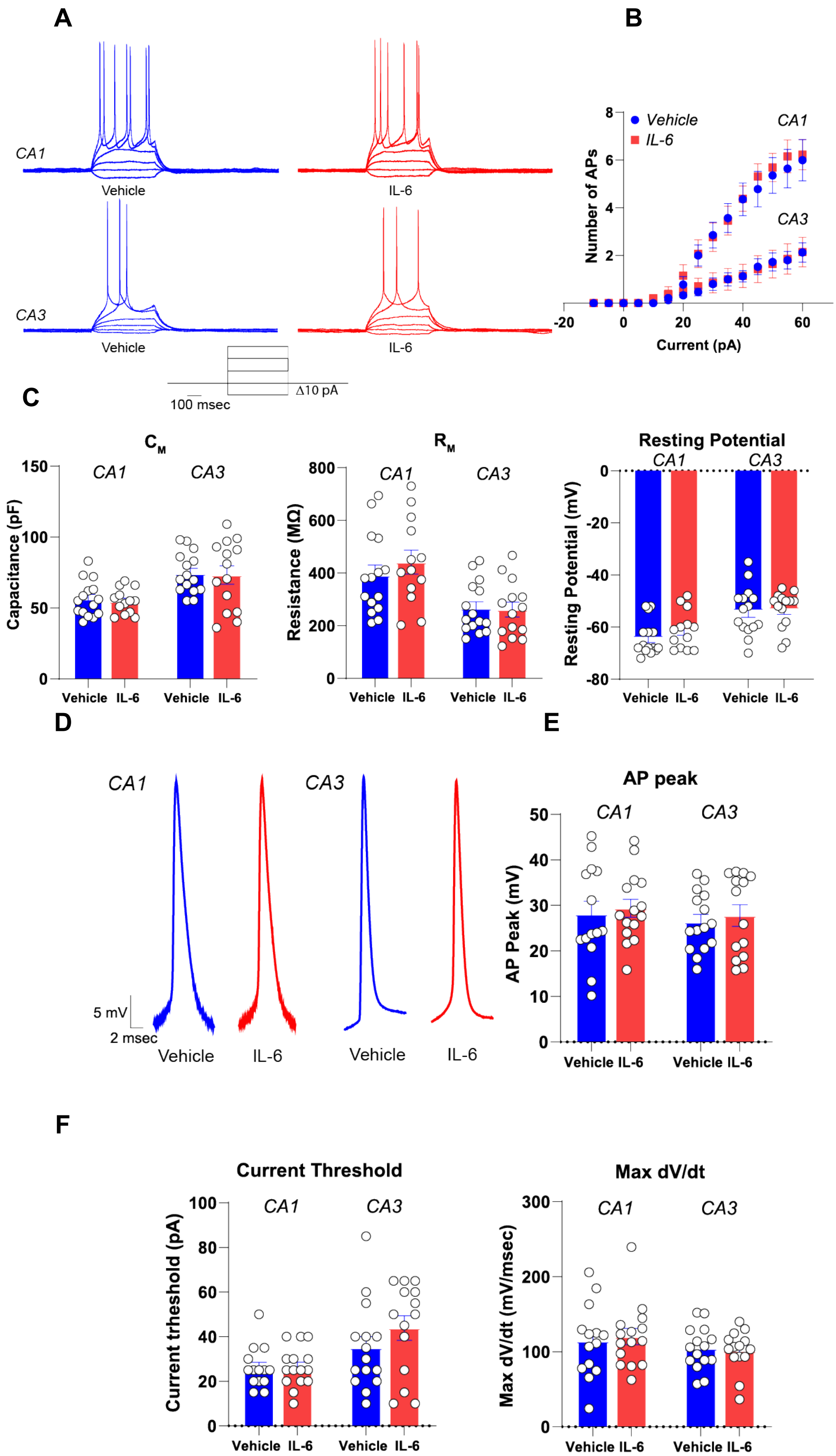


***Figure R1.2**. **A**. Representative traces of intrinsic neuronal excitability recorded in whole-cell current-clamp configuration from CA1 and CA3 pyramidal neurons in acute brain slices of P5 mice treated with Vehicle (blue) or IL-6 (red); **B.** Quantitative analysis of the number of action potential in response to depolarizing current steps in CA1 and CA3 pyramidal neurons under both conditions. **C.** Quantification of passive*

*membrane properties, including Capacitance (CM), Membrane resistance (RM), and Resting potential. Data are presented as mean ± SEM of n=15 vs n=13 cells for CA1, n= 15 vs n= 14 for CA3, from N=2 Vehicle and N= 2 IL-6 exposed mice, from two independent experiments*. Two Way ANOVA, ns, p> 0.05; **D.** *Representative action potential waveforms recorded from CA1 and CA3 hippocampal pyramidal neurons in acute brain slices from P5 mice treated with Vehicle (blue) or IL-6 (red);* ***E-F.*** *Quantification of* Action potential (AP) peak, Current threshold, and maximum rate of rise of the membrane potential (maximal dv/dt). Data are presented as mean ± *SEM of n=15 vs n=13 cells for CA1, n= 15 vs n= 14 for CA3, from N=2 Vehicle and N= 2 IL-6 exposed mice, from two independent experiments. Two Way ANOVA, ns, p> 0.05.*

3. When comparing between age groups, the MQAE fluorescence should be calibrated at the end of the experiments, or the results should be confirmed with recordings of GABA-A reversal potential.

**We thank the reviewer for the opportunity to clarify this point. The MQAE fluorescence between the two age groups, at P5 and P15, was evaluated by simultaneously recording two mice at different ages within the same experimental day. This approach ensured that measurements of MQAE relative fluorescence were obtained under strictly identical experimental conditions (in terms of slicing procedure, incubation, dye loading, external solutions and multiphoton recording). The same procedure was also used when comparing mice of the same age (Vehicle vs IL-6). We apologize if this detail was not clearly described in the Materials and Methods section. In the revised manuscript, we have included this technical information. In addition to mean fluorescence measurements at the two developmental time point, we now also provide FLIM decay time measurements, a more stable value that is independent of dye loading and other potential environmental issues arising during the experimental day (see Figure R1.3 A). Calibration of FLIM parameters has already been performed for this dye in our previous work [15], as shown in the Stern-Volmer plot below (see Figure R1.3 B) revealing a Stern–Volmer constant (Ks) of 31 $M^{-1}$. By applying this calibration parameter to the Stern–Volmer relationship for lifetime measurement ($\tau 0/t=1+Ks[Cl^{-}]$, with the control fluorescence lifetime of 5.4 ns) we estimated intracellular chloride concentrations as follow P5: 22,4 mM; P15: 15.2 mM. Considering the extracellular chloride concentration in our conditions (135.2 mM), the estimated intracellular chloride concentrations derived from FLIM measurements correspond to chloride equilibrium potentials ($E_{Cl}$ of approximately −48 mV at P5 and −58.4 mV at P15. These data indicate a 10 mV hyperpolarizing shift from P5 to P15, fully consistent with developmental chloride extrusion. In addition, considering the membrane potential measured at P5 (see Figure 1 manuscript), the chloride driving force ($V_m - E_{Cl}$) is approximately −8 mV, reflecting, as expected, a depolarizing action of GABA at this developmental stage. The FLIM data has been included in the Fig. S2A of the revised manuscript.**

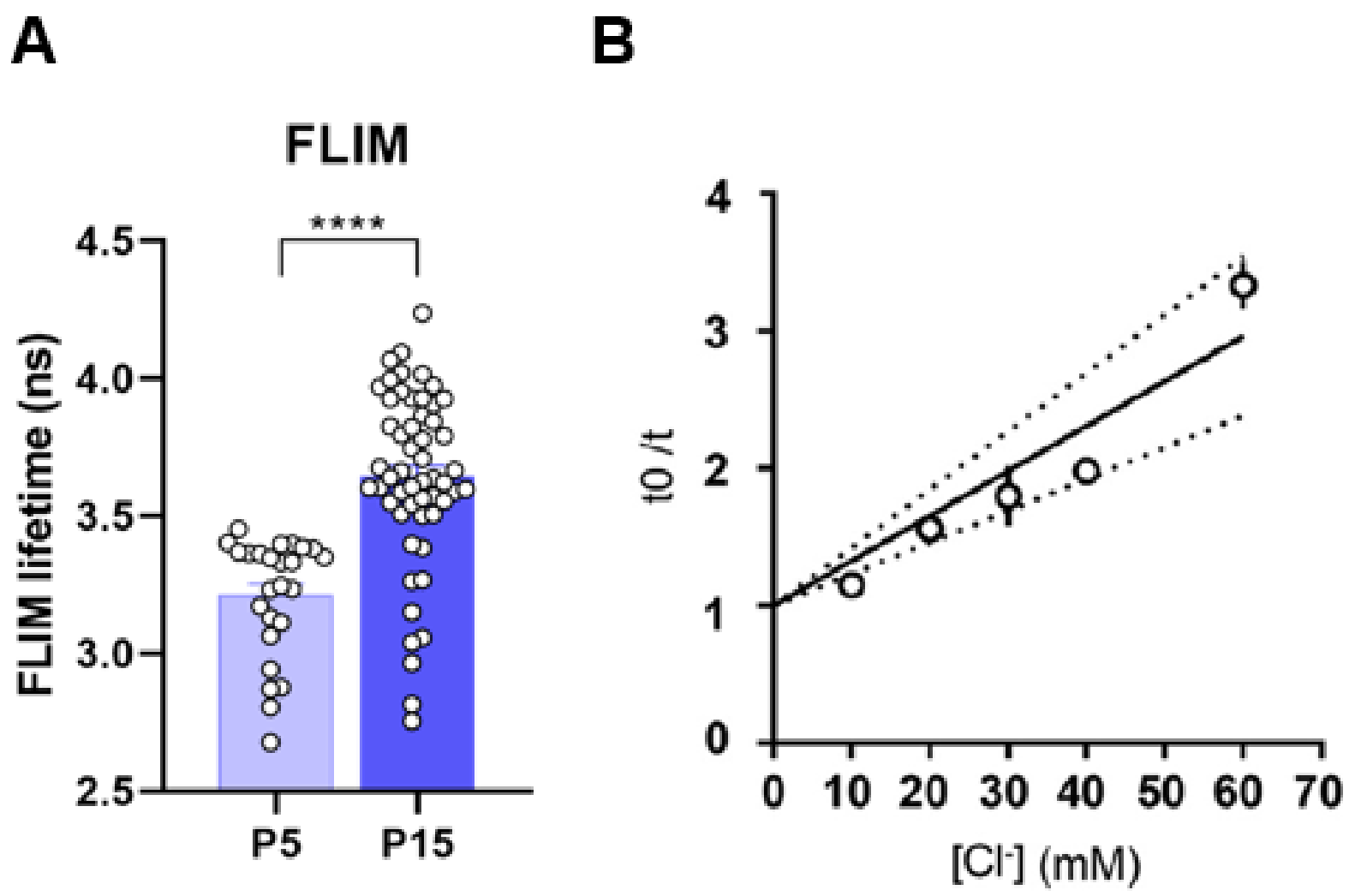

***Figure R1.3**. **A.** Fluorescence Lifetime Intensity Microscopy (FLIM) measurements between P5 and P15 experimental conditions (dots represent the number of analyzed cells). Unpaired t test, $p < 0.0001$. **B.** From previous work [15]: Stern-Volmer plot of MQAE quenching at a given Cl– concentration, allowing determination of intracellular chloride concentration. The plot shows the ratio of fluorescence lifetimes without and with the quencher ($t_0/t$) against the quencher's concentration ($[Cl^-]$). A linear relationship indicates dynamic (collisional) quenching*

4. Why the basal frequency of LFPs in Figure 3L is not different between the groups (control vs Il-6) and is two times higher as compared to corresponding data in Figure 1B? Also, please indicate if the raster plots represent activity before or after drug application.

**Concerning the basal frequency of LFPs shown in Figure 3L, these data were obtained from different experimental groups of animals, which might explain the differences in the basal frequency. Moreover, they were derived from unmatched vehicle and IL-6 animals, recorded on different experimental days rather than simultaneously. In this experiment, our aim was to evaluate how network activity in the two conditions (Veh and IL-6) changed upon VU application. A simultaneous comparison of the four experimental conditions (Veh ± VU and IL-6 ± VU) within the same experimental day would have been too technically challenging due to the duration of each recording session. However, it should also be noted that, although the statistical significance between the Veh and IL-6 groups was not as strong as in Figure 1 (owing to the use of unmatched vehicle and IL-6 animals), the observed trend remained consistent.**
**Regarding the raster plots, the "baseline" conditions represents the activity before drug application, whereas the condition "+ VU 0463271" the activity after drug application. This information was reported in the figure legend of the original manuscript (Representative raster plots with SNEs and LFPs in-set from either Vehicle or IL-6 exposed P5 mice before and after VU0463271 administration (left). However, to improve the self-readability of the figure, this information will be better specified with a more detailed description of the experimental setting in the material and methods section.**

5. It is not clear why in figure 4I-K, STAT3 phosphorylation levels are assessed instead of expression levels. Is STAT3 phosphorylation increased in IL6 treated mice?

**As previously demonstrated in our work, [14] IL-6 induces the transcriptional upregulation of STAT3 and its phosphorylation levels both in vivo and in vitro. Moreover, the functional activity of STAT3 is tightly regulated by its phosphorylation state, as widely reported [16] [17-19] Hence, in our new work we used then the phosphorylation levels assessment as a proxy of the proper IL-6 pathway activation. This concept has been better clarified in the results.**

6. Figure S4 the image quality (example traces) is not sufficient. For the data in figure S4M, please show expanded traces of the individual events, to illustrate the detection criteria for the low-amplitude events vs noise. Please also include the details used for event detection in the methods.

**In response to the reviewer's request, we now provide an expanded trace from panel S4M (Figure R1.4), allowing individual events to be clearly visualized and the detection criteria for low-amplitude events to be better appreciated. However, at this expanded time scale the entire sweep recording cannot be fully displayed and appreciated. For that reason, to provide an overall view of the recording quality across the entire trace, and for consistency with other panels, we would prefer maintain the previous time scale (60 sec of recordings) in order to show the entire recording sweep. For better clarity and readability of the image, we will also implement the methods paragraph by adding specific details on event detection and analysis.**

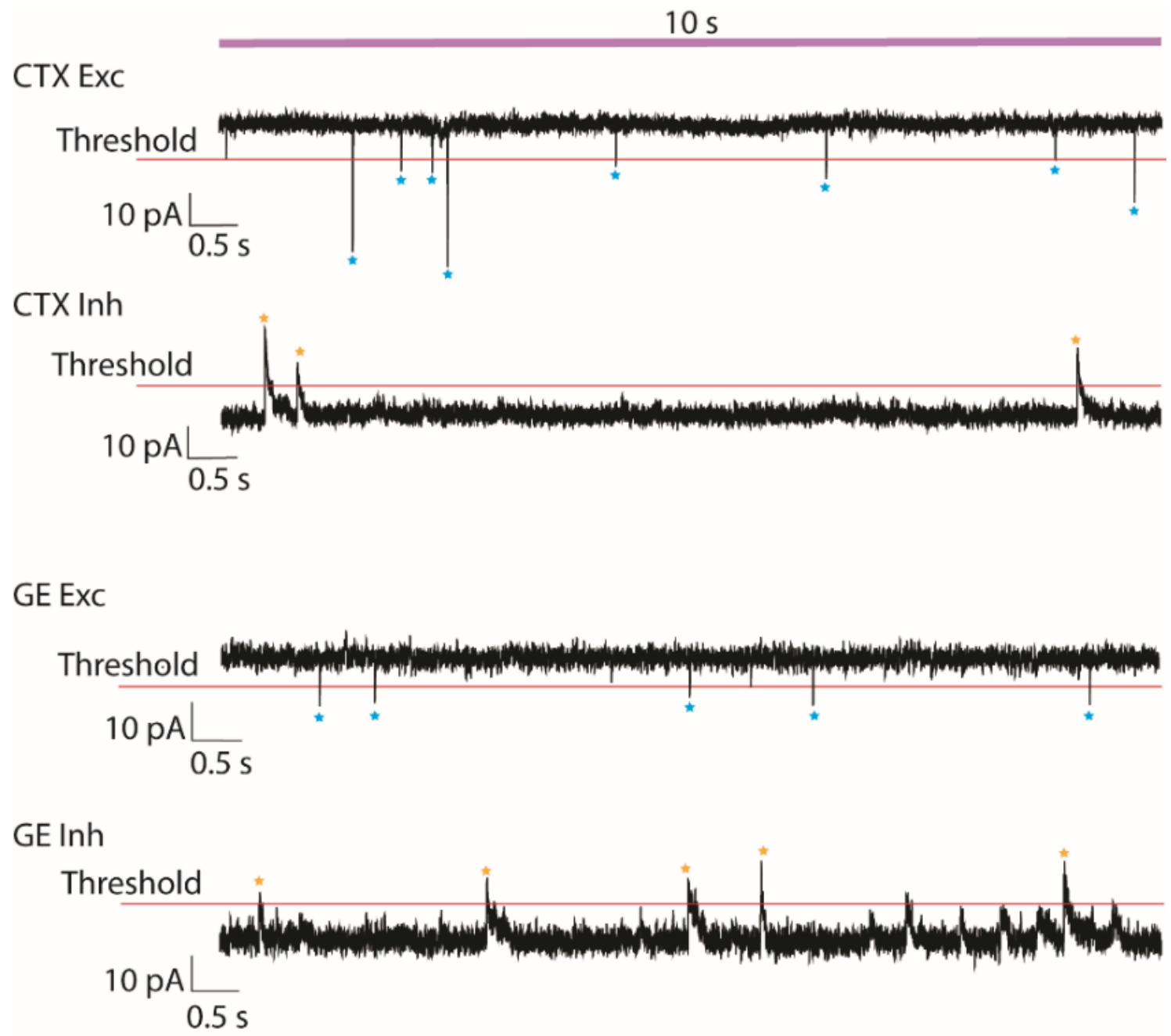


**Figure. R1.4.** *Expanded traces referring to figure S4M showing the event detection, threshold setting and peak analysis of mIPSCs and mEPSCs on GE and CTX cultures. Scale bar 10 pA, 0.5 secs.*

*7.* In figure 5, the authors show that treatment of hippocampal cultures with bicuculline prevents the IL-6 -induced increase in KCC2 expression. However, bicuculline treatment results in homeostatic plasticity, involving a variety of compensatory changes that could indirectly influence [Cl-]i and KCC2 levels. To argue for causal relationship between early GABAergic activity and upregulation of KCC2 expression (Fig 5), the authors should use treatments that do not fully block GABAergic activity, but reduce it to control levels

**The choice to use Bicuculline to reduce GABAA receptor activity and test the effect on KCC2 expression in IL-6 treated neurons follows previously published work based on similar approach (e.g., [20,21], where authors have shown that GABA signaling is key for the GABA developmental switch. This finding was fully confirmed by our study (see Supplementary Figure 5B), where cultured neurons chronically treated (from 1 -4 DIV) with either Bicuculline or GABA display, respectively, a higher and lower intracellular chloride concentration if compared to control neurons (Supplementary Figure 5b). These evidences indicate that the blockade of GABA signaling do not elicit a compensatory mechanism but rather delay the GABA switch as indicated by a blockade of KCC2 upregulation. In addition to that, the KCC2 expression level did not change between cultured neurons treated with Bicuculline alone and in combination with IL-6 (Figure 5B), indicating that the GABAA-receptor inhibition totally occludes the IL-6 effect. Based on these considerations, a potential compensatory mechanism induced by Bicuculline is highly unlikely. However, to directly address the reviewer's concern, GABAergic transmission of IL-6–treated neurons was recorded in the presence of increasing concentrations of Bicuculline (0, 0,1 1, 2,5 µM) to determine the minimal dose required to restore synaptic basal frequency to control levels (Fig. R1.4). The dose–response relationship showed a progressive reduction in event frequency with increasing Bicuculline concentrations, consistent with a monoexponential decay profile. By extrapolating the fitted curve to its intersection with control levels, we estimated that ~2 µM Bicuculline is the minimal concentration required to restore IL-6–enhanced GABAergic transmission to baseline (Fig R1.5 A). We then assessed KCC2 transcriptional expression in IL-6 treated neurons in the presence of 2 µM Bicuculline and we found no differences compared to neither the control condition and 50 µM Bicuculline. Furthermore, 2 µM Bicuculline does not alter KCC2 expression in untreated neurons, similar to 50 µM Bicuculline (Fig R1.5 B). This experiment indicates that premature KCC2 upregulation can be occluded even with a Bicuculline concentration sufficient to restore physiological GABAergic**

**transmission in IL-6 treated neurons, and therefore rules out any potential compensatory mechanisms hypothesized by the reviewer. However, as these data do not substantially extended the conclusions of the study, we would prefer to provide them in response to the reviewer's request without including them in the revised manuscript.**

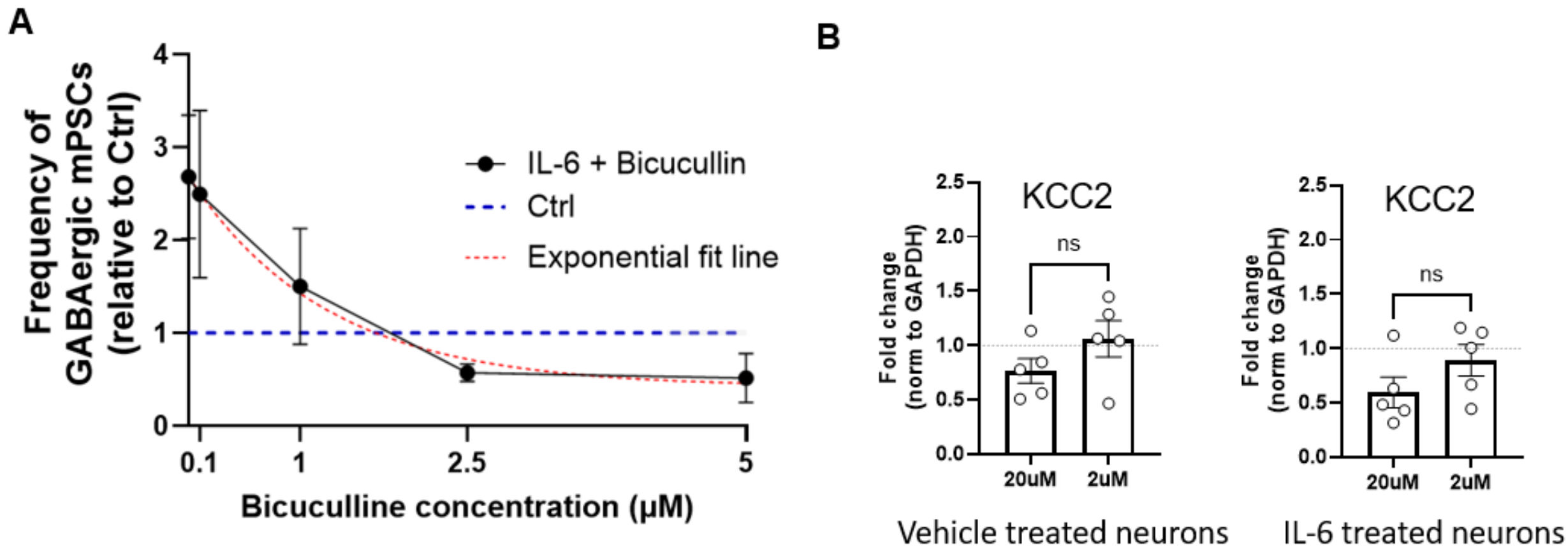


**Fig. R1.5. A**. *Graph showing the frequency of GABAergic postsynaptic currents in IL-6 treated hippocampal neurons as a function of increasing concentrations of* $GABA_A$ *receptor antagonist Bicuculline (Bic). Data are normalized against Ctrl condition (dotted blue line) and presented as mean ± SEM. Ctrl n= 6; IL-6 n= 9; IL-6 +Bic 0.1 µM n = 7; IL-6 + Bic 1 µM n= 7; IL-6 + Bic 2.5 µM n= 5; IL-6 + Bic 5 µM n= 7 cells, from N= 2 independent experiments. Intercept = 2 µM.* ***B****. Quantification of KCC2 mRNA expression (relative to Gapdh) in hippocampal neurons in ctrl condition and upon IL-6 treatment. Data are presented as mean ± SEM of N=5 independent experiments.*

8. Discussion (e.g. paragraph starting from row 339) contains several statements that are not supported by the manuscript data and without references to previous publications.

**We thank the reviewers for highlighting this point. To support the following statement "*While the occurrence of SNEs reflects the intrinsic tendency of neurons to spontaneously fire, the intensity and duration of the associated discharge (the MFPR) reflect the strength of synaptic excitatory signaling*", we have now included the following references in the revised manuscript [22-24]. In these independent papers, the authors showed that intrinsic currents from depolarizing sodium channels play a decisive role in mechanisms leading to burst onset [22]. Moreover, the generation of burst is also tightly dependent on the activity of a limited number of neurons that trigger network events [23,24]. Finally, regarding the intensity and duration of the associated discharge, it has been reported that modifying excitatory connectivity or synaptic interactions significantly alters burst properties, including duration and intensity [22,25,26]. Hence, based on these evidences, we would like to rephrase our statement as follow "*While the occurrence of SNEs reflects the intrinsic tendency of specific leader neurons to spontaneously fire* [22-24]*, the intensity and duration of the associated discharge (the MFPR) reflect the strength of synaptic excitatory signaling as previously reported* [22 25,26]"**

**Regarding the statement "*The functional changes induced by prenatal IL-6 exposure in hippocampal slices at P5 reveal that, rather than altering episodes of synchronous network events (SNEs), IL-6 primarily affects the frequency of local field potentials (MFPR)*" this sentence simply reflects a description of our data. Indeed, IL-6 primarily affects MFPR frequency without significantly altering SNEs frequency.**

9. Discussion line 372 : The manuscript does not contain data to show that the enhancement of the GABAergic transmission elicited by IL-6 precedes the GABA switch in acute brain slices. The mIPSC data in acute slices are from P5.

**According to the reviewer's request, the GABAergic postsynaptic currents were evaluated at P3. Consistent with the upregulation of GABA-related genes** (Gad67, ***Slc32a1*****, and** ***Gphn*****) (Figure 5I), but not the KCC2-encoding gene** ***Slc12a5*** **(Figure 5L), the basal GABAergic transmission was found to be significantly enhanced even at this developmental stage (Fig. R1.6). This new finding further confirm our hypothesis and the result was included in the revised manuscript and related figure 5J-K.**

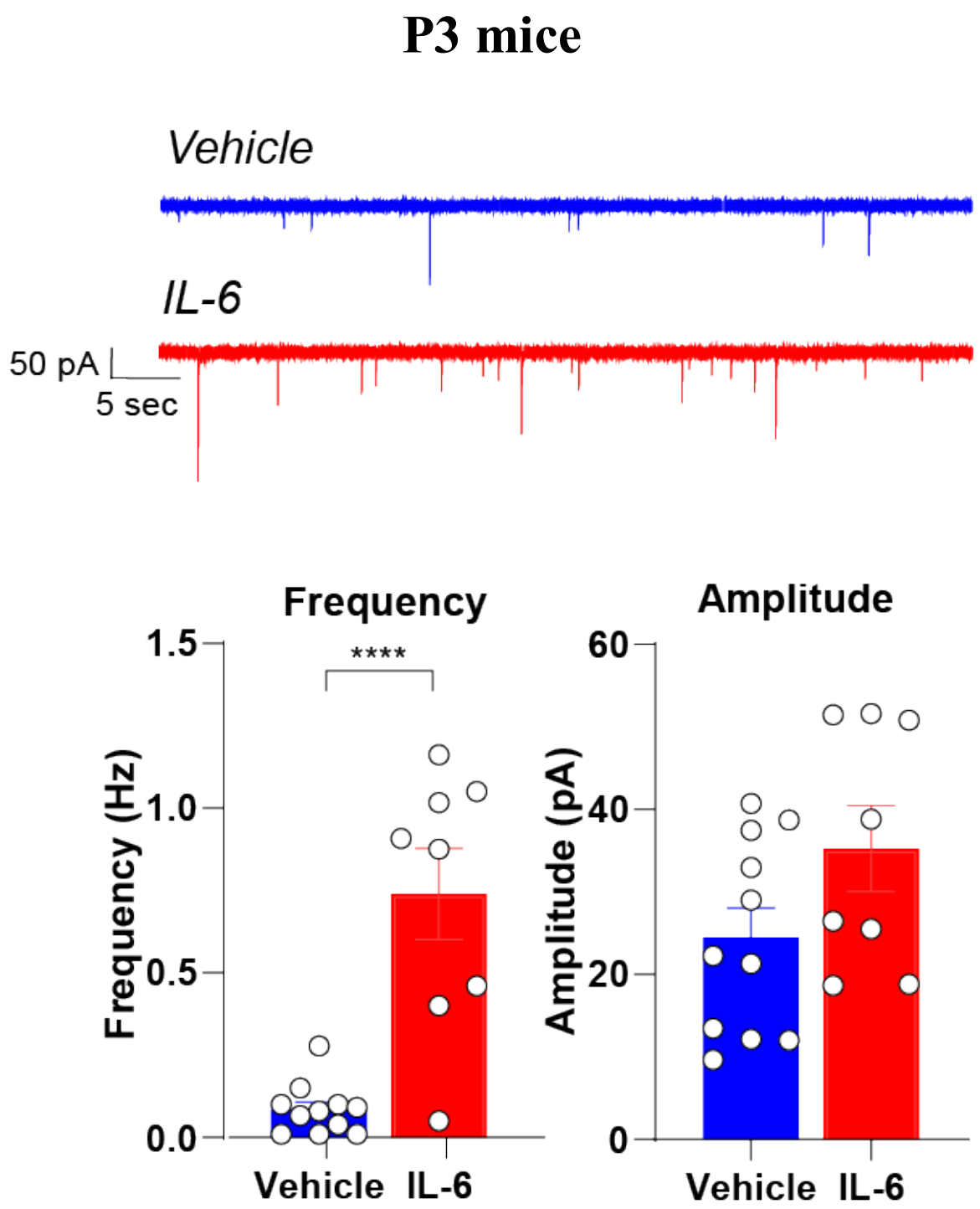


**Figure R1.6. Top:** *Representative traces of GABAergic mPSC recorded in the CA1 pyramidal neurons of acute slices established from P3 mice prenatally exposed to Vehicle or IL-6.* **Bottom:** *Histograms showing mPSCs Frequency (left) and Amplitude (right) of n=11 vs n=8 cells from N=3 Vehicle and N=3 IL-6-exposed P3 mice, from three independent experiments. Data are presented as mean ± SEM. Unpaired t test, ****, $p < 0.0001$.*

10. Minor: in all figures containing MEA recordings, please indicate which hippocampal subfield the data in the raster plots represents

**Our experimental design enables the recording of overall hippocampal network activity without specifically assessing activity within individual subfields (e.g., CA1, CA3, dentate gyrus). Therefore, we are unable to provide subfield-specific data for the current raster plots.**

**Reviewer #2 (Remarks to the Author):**

The study "Elevated prenatal IL-6 levels disrupt early patterns of hippocampal electrical activity 2 by targeting GABAergic signaling at perinatal stage" by Desiato et al. was evaluated. The work demonstrates that a transient prenatal elevation of interleukin-6 (IL-6) at gestational day 15.5 significantly alters the normal maturation of hippocampal network activity in newborn mice by accelerating the developmental switch of GABAergic signaling. Using high-density multi-electrode array recordings, the authors found that at postnatal day 5 (P5), mice prenatally exposed to IL-6 exhibited significantly reduced hippocampal spiking activity

compared to controls, despite unchanged network synchrony and intrinsic neuronal excitability. This disruption was not due to early inflammation, as immune profiling and cytokine gene expression analyses showed no signs of local or systemic immune activation.

Instead, IL-6 induced a premature reduction in intracellular chloride concentration in CA1 pyramidal neurons by upregulating the potassium-chloride cotransporter KCC2 via STAT3-dependent transcriptional mechanisms. This led to a premature shift in GABA signaling from excitatory to inhibitory. Critically, IL-6 was shown to directly enhance early GABAergic synaptic activity by acting on GABAergic neurons, again through STAT3 signaling. This enhancement occurred before KCC2 upregulation and was necessary for the developmental switch in GABA polarity. Blocking GABAergic activity prevented IL-6-induced KCC2 expression and chloride reduction, establishing a causal link. Finally, this altered signaling cascade disrupted early behavioral responses in IL-6-exposed mice when KCC2 was pharmacologically inhibited, further confirming its functional relevance.

Novelty/originality

The work describes new results, the data obtained are significant and highly original. The paper uniquely demonstrates that transient prenatal IL-6 elevation directly alters early hippocampal network development by enhancing GABAergic transmission and accelerating the GABA polarity switch via STAT3–KCC2 signaling, without inflammation. This mechanistic clarity and early developmental timing clearly distinguish it from prior MIA studies (e.g., Smith et al., 2007; Shin Yim et al., 2017).

The work supports its conclusions with well-controlled experiments across mainly in ex vivo models, using some in vivo experiments. It uses multiple techniques (HD-MEA, patch-clamp, imaging, gene expression) and causal interventions (IL-6, STAT3 inhibitors, KCC2 blockade) to establish mechanistic links. While longitudinal behavioral data would strengthen clinical relevance, the current evidence supports the main mechanisms, although the main causal points should be tested in vivo as well.

Methodology is generally sound and employs well-established techniques to assess neural network activity and chloride homeostasis. There is sufficient detail for reproducibility, including experimental time points (e.g., P3, P5), IL-6 dosing, cell culture timelines, and the use of specific inhibitors (e.g., Stattic, VU0463271).

In general the study is strong, but heavily relies on ex vivo models (slices and cultures). It would make the material much stronger, if a few more in vivo experiments would be added.

There are a few specific points to address:

**We sincerely thank the reviewer for the very positive comments to our work.**

1. It would be good to confirm the mechanisms with more robust in vivo experiments. (Stattic or Gal interventions in vivo for example, or an elegant 4-group experiment – ctrl+vehicle, ctrl+KCC2 inhibitor, IL-6 + vehicle, IL-6 + KCC2 inhibitor)

**We thank the reviewer for this comment, with which we fully agree, and we recognize the value of validating our proposed mechanisms in vivo. Regarding the reviewer's suggestion to use STAT3 inhibitors, it should be noted that in vivo administration of Stattic or Galiellalactone would likely have severe systemic consequences, given their broad inhibitory effects on STAT3 signaling in multiple tissues, especially during development [27]. Such interventions could induce confounding physiological alterations unrelated to the neural mechanisms under investigation, potentially compromising animal welfare and making the interpretation of results difficult. For these reasons, the use of Stattic or Galiellalactone in vivo would be technically challenging and potentially unsuitable within the current experimental framework.**

With respect to the proposed ***"elegant 4-group experiment – ctrl + vehicle, ctrl + KCC2 inhibitor, IL-6 + vehicle, IL-6 + KCC2 inhibitor"***, **this paradigm has already been implemented in vivo in P5 pups (Figure 3M), where the readout was the maternal odor preference (MOP). In the revised version of the manuscript we have included an immunofluorescence analysis of the immediate-early gene, cFos, in the hippocampus following the MOP test, confirming the activation of the hippocampus during this behavioral task (see supplementary Figure 3C-D of revised manuscript). Moreover, the MOP test was also repeated at a later developmental stage, P10, at which KCC2 upregulation persisted. Interestingly, at this time point mice prenatally exposed to IL-6 exhibited impaired odor discrimination and the pharmacological inhibition of KCC2 with VU0463271 reversed this deficit (Fig. R2.1 A). In light of these findings at P10, USV recording was also performed at this stage. Consistent with the MOP results, the number of USVs was found to be altered in IL-6 exposed mice at P10 and treatment with the KCC2 inhibitor rescued this abnormality (Fig. R2.1 B). We believe that these new results add strength to our study (also addressing other issues raised by the reviewers, for instance see point 2 reviewer#2 and see point 3 raised by reviewer#3), indicating that behavioral alterations in pups prenatally exposed to IL-6 emerge at later stages than P5 and, once detectable, could be pharmacologically targeted with KCC2 inhibitor.**

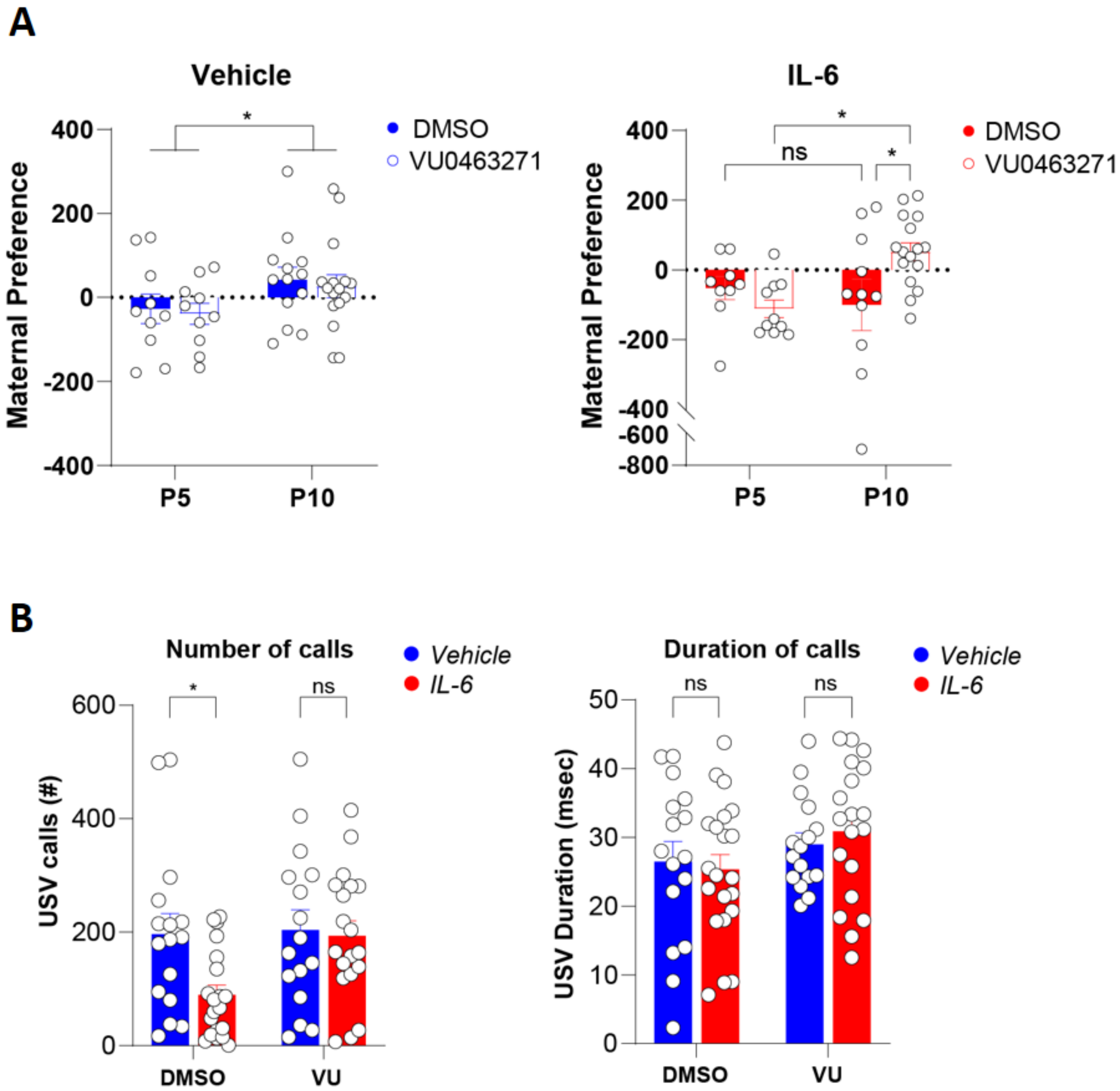


**Figure R2.1. A.** *Maternal Odor Preference Test. Graphs showing maternal preference quantification of the Time- in- zone spent in the "mother" or "clean" shaving in Vehicle or IL-6 exposed P5 vs P10 mice treated with VU0463271 30 minutes before the test. Dots represent for P5 mice, N=10 Vehicle + DMSO vs N=10 Vehicle + VU, vs N=79 IL-6 + DMSO, N=10 Vehicle + VU, vs N=109 IL-6 + VU; for P10 mice, N=13 Vehicle*

*+ DMSO, vs N=16 Vehicle + VU, N=10 IL-6 + DMSO vs N=16 IL-6 + VU from at least three independent experiments. Vehicle: Two-Way ANOVA (main effect of treatment: p = 0.6555; main effect of age: p = 0.0311; treatment x age interaction: p = 0.9598) followed by between- group Sidak multiple comparison test, ns, p > 0.05. IL-6: Two-Way ANOVA (main effect of treatment: p = 0.2946; main effect of age: p = 0.1960; treatment x age interaction: p = 0.0207) followed by between- group Ŝidàk's multiple comparison test, *, p < 0.05.* **B.** *Ultrasonic vocalization (USV) test. Graphs showing quantification of the total number (left) and duration (right) of the calls vocalized by Vehicle or IL-6 exposed P10 mice with or without VU administration 30 minutes before the test. Data are presented as mean ± SEM of N= 16 Vehicle + DMSO, N=16 Vehicle + VU, N= 20 IL-6 + DMSO, N= 19 IL-6 + VU exposed mice from at least three independent experiments. Number of calls: Two-Way ANOVA (main effect of IL-6: p = 0.0429; main effect of drug: p = 0.0549; IL-6 x drug interaction: p = 0.0929) followed by between- group Ŝidàk's multiple comparison test, *, p < 0.05. Duration of calls: Two-Way ANOVA (main effect of IL-6: p = 0.8743; main effect of drug: p = 0.0883; IL-6 x drug interaction: p = 0.5169) followed by between- group Ŝidàk's multiple comparison test, ns, p > 0.05.*

2. The behavioral assessments are limited to early postnatal stages; long-term consequences of the early IL-6-induced changes are inferred but not directly tested. It would be good to see a longer-term effect.

**We thank the reviewer for this comment. The present study specifically focuses on early postnatal stages to investigate the initial effects of prenatal IL-6 on hippocampal network development and early behavior. Importantly, the long term behavioral consequences in adult offspring have already been extensively evaluated and discussed in our previous work (Mirabella *et al.*, 2021), which demonstrated specific impairment in hippocampal-dependent spatial memory, with no alterations in the domains related to anxiety and locomotion. Therefore, the current study complements these findings by providing a detailed characterization of the early postnatal window. Nevertheless, in addition to the results shown in Mirabella *et al.*, 2021 and to further characterize the long-term behavioral consequences of prenatal IL-6 exposure in adult offspring, we recently performed sociability test in a new cohort of vehicle and IL-6-exposed mice at during early adulthood stages (P60) using three chambers test. Interestingly, both vehicle- and IL-6–treated mice showed a clear preference for the social stimulus over the inanimate object, indicating preserved social preference (Fig. R2.2). However, IL-6–treated mice spent significantly less time in direct object exploration, automatically quantified as time in object proximity (Fig. R2.2). This reduction may reflect alterations in sensory processing [28], increased avoidance of inanimate objects [29], or reduced object-directed exploration secondary to an enhanced motivational salience of the social stimulus as observed in mouse models of ASD [30]. While we believe this additional observation in adult offspring is highly relevant, this finding is not directly related to the main focus of the present manuscript. Given this consideration, and in the absence of a defined underlying mechanism, we believe that including these data in the current manuscript would be premature and beyond the scope of the current study. Instead, we plan to further characterize this behavior in a separate work.**

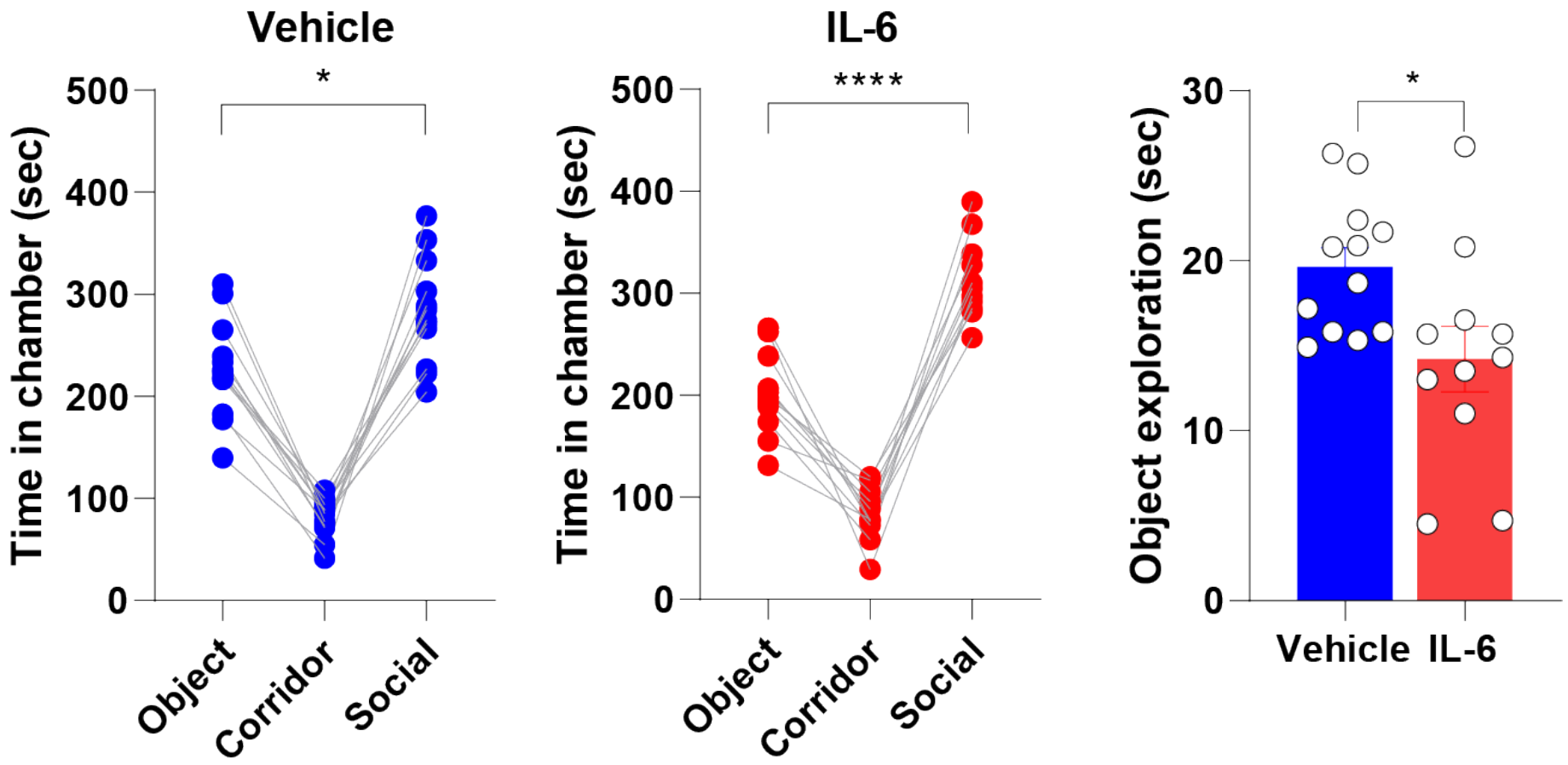


**Fig. R2.2.** *Three chamber test. From left to right, graphs showing the time spent in zone (object, corridor, social) of Vehicle (Left) and IL-6 (middle) exposed mice at P60. Right: histograms showing time spent for object exploration in Vehicle and IL-6 exposed mice. Dots represent N= 12 Vehicle vs N=11 IL-6 exposed P60 mice. Time in chamber: Repeated Measures One Way ANOVA, p < 0,0001 followed by Sidak's post hoc Object vs Social * p<0.05; **** p< 0.0001; Object Exploration: Unpaired t test, * p < 0.05.*

3. The maternal odor preference test is modest in scope—more diverse behavioral assays would strengthen conclusions about functional impact.

**As already described in the previous points, in our previous publication [14] we performed multiple behavioral tests at later stages. In addition to these published results, the MOP and USV tests were repeated at P10, (a stage at which KCC2 upregulation was maintained) and sociability was assessed in adulthood (see points 1 and 2 above)**

4. Neuronal networks (connectivity, synapse density, neuronal morphology, etc..) should be assessed in vivo at later postnatal timepoints.

**We thank the reviewer for this comment. The long-term consequences of the IL-6 model on connectivity, synaptic density, and neuronal morphology have already been evaluated and discussed in our previous work (Mirabella et al., 2021). In that study, we reported that adult offspring prenatally exposed to IL-6 display hippocampal hyperconnectivity associated with an imbalance in excitatory/inhibitory synaptic inputs, as well as hippocampal-dependent behavioral deficits, while no gross morphological alterations were detected. To better emphasize these previously published findings, we now explicitly reference our earlier study in the "Discussion" paragraph and include an additional sentence integrating those results with the current findings.**

5. While GABAergic cultures are used, in vivo confirmation of IL-6's selective action on GABAergic neurons (e.g., via conditional STAT3 knockouts, central pharmacological inhibition) would improve mechanistic precision.

**We thank the reviewer for this comment. We fully recognize the importance of corroborating our in vitro evidence with in vivo evidence for a direct role of IL-6 on GABAergic neurons. In fact, we have already attempted this, although with limited success due to technical constraints. Specifically, we tested a chemogenetic approach to selectively inhibit GABAergic neurons in pups, by stereotaxically injecting an AAV expressing a Gi-DREADD under the control of the hDlx enhancer element into the hippocampus at P0. However, the virus required at least 10 days for sufficient expression, preventing us from performing the experiment at P5. An alternative strategy would be to use transgenic animals.**

**While conditional knockout models (e.g., selective STAT3 deletion in GABAergic neurons) could in principle address this question, their generation is technically demanding and time-consuming, and was therefore not pursued in the present study. Instead, we performed a novel in vivo experiment that provides new mechanistic insight into the study. To determine whether the enhancement of GABAergic signaling induced by IL-6 was causally linked to the premature upregulation of KCC2, pups prenatally exposed to IL-6 or vehicle were treated with picrotoxin (a GABAA receptor antagonist that crosses the blood-brain barrier) from P1 to P4, and KCC2 transcriptional expression was assessed in the hippocampus at P5. We found that the abnormal upregulation of KCC2 observed in IL-6 exposed mice was prevented by picrotoxin treatment, indicating that the early increase in GABAergic transmission induced by IL-6 was required for the subsequent upregulation of KCC2 (Fig. R2.3). This new finding corroborated our data and indicate the precise sequela of events linking early GABAergic transmission, network activity and KCC2 disruption.**

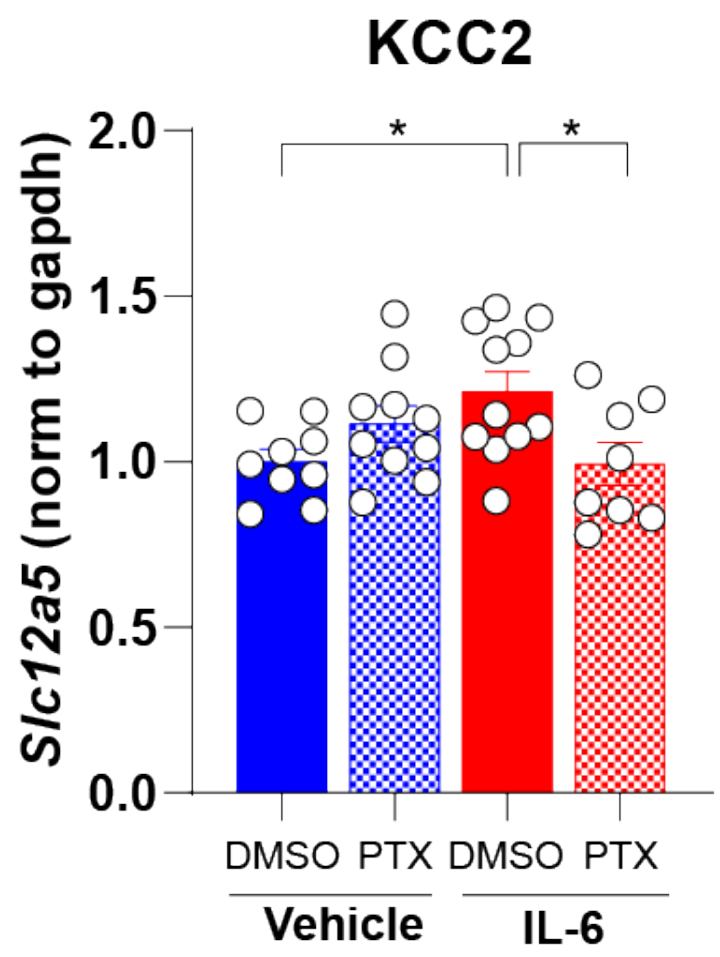


**Figure R2.3.** *Quantification of mRNA relative expression of chloride transporter Kcc2, in the hippocampi of Vehicle and IL-6 exposed P5 mice with or without Picrotoxin (PTX) i.p. administration. Data are presented as mean ± SEM of N=9 Vehicle + DMSO, N=10 Vehicle + Ptx, N=11 IL-6 + DMSO, N=8 IL-6 + Ptx,-exposed mice from at least three independent experiments. One Way ANOVA with Tukey's multiple comparison test, ns $p < 0.05$.*

6. It would be good to see the developmental window of IL-6 sensitivity. For example, injecting IL-6 directly into pups at P1–P3 and assessing hippocampal network activity and KCC2 expression at P5 would confirm whether the mechanism is time-sensitive.

**In accordance with the reviewer's suggestion, IL-6 was administered at postnatal days 1–3, and the expression of chloride transporters, KCC2 (Slc12a5) and NKCC1 (Slc12a2), as well as GABA-related genes, were evaluated at P5 in the hippocampus. Our results show that postnatal IL-6 exposure does not significantly affect the transcriptional expression of most of the target genes altered in our model, with the exception of NKCC1 transcript *Slc12a2*, which was significantly upregulated (Fig. R2.4). These results suggest that IL-6 exposure at postnatal stages induces a delay rather than an acceleration of the developmental GABA switch, a result that stands in contrast to our prenatal findings. These findings indicate that the timing of IL6-mediated immune activation represents a critical determinant of offspring phenotype, with the prenatal period constituting the primary window of vulnerability for the embryonic hippocampus leading to enhanced GABAergic signaling and premature KCC2 expression. We thank the reviewer for the insightful suggestion, which has helped clarify the roles of IL-6 at different developmental stages. However, since this model does not directly align with our prenatal model of IL-**

**6 elevation, we prefer not to include these date in the revised manuscript. However, should the reviewer consider it necessary, we are ready to include these data in a revised version.**

**P5 mice treated postnatally with IL-6 - 200 ng**

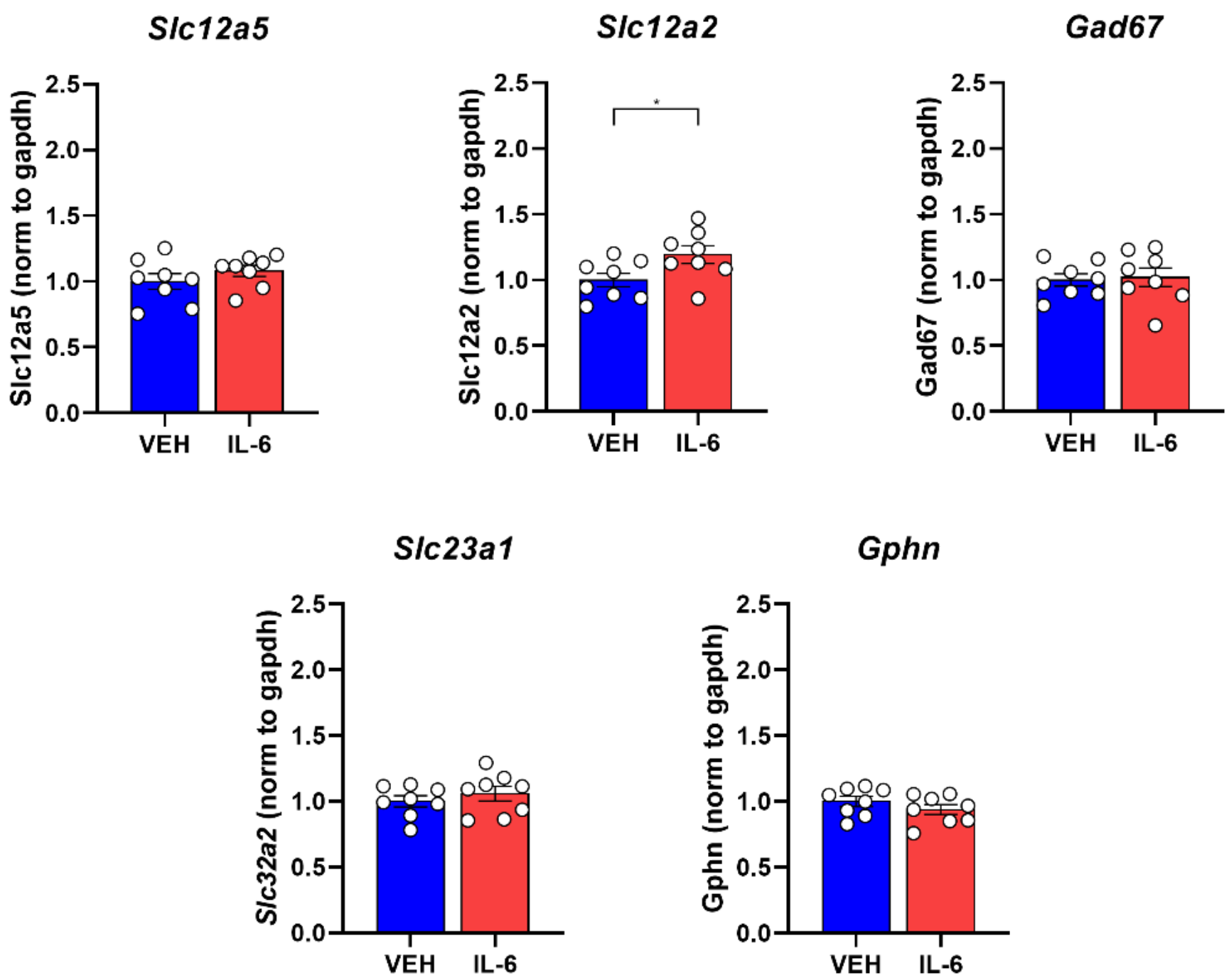


**Fig. R2.4.** *Quantification of mRNA relative expression of chloride transporters KCC2, Slc12a5, and NKCC1, Slc12a2, and GABA-related encoding genes (Gad67, Slc32a1, Gphn) in the hippocampi of Vehicle and IL-6 exposed mice. Data are presented as mean ± SEM of N=8 Vehicle vs N=8 IL-6 postnatally exposed P5 mice from al least three independent experiments. Unpaired t test, ns > p 0.05, * p < 0.05.*

7. It would also be good to strengthen the temporal relationship between IL-6, GABA-related gene expression, and KCC2. By collecting hippocampal tissue from control and IL-6-exposed pups at P3, P5 and P10, and measuring expression of Gad67, Slc32a1, Kcc2, and Stat3 over time, the Authors could confirm sequential expression pattern.

**In agreement with the reviewer's observation, we evaluated the transcriptional levels of several genes at P10, allowing us to provide a more comprehensive picture of the temporal expression pattern of *Kcc2* and other GABA-related genes. Consistent with the results obtained at P5, KCC2 transcript upregulation persisted even at P10, without changes in NKCC1 expression. This new evidence further corroborates our behavioral analysis performed at P10, where *in vivo* blockade of KCC2 activity via VU0463271 rescued the alteration observed in the MOP and USV tests and have been added to the revised version of the manuscript. Notably, other GABA-related genes (*Gad67*, *Slc23a1*, *Stat3*) were found unchanged at P10 (Fig. R2.5).**

**P10 mice**

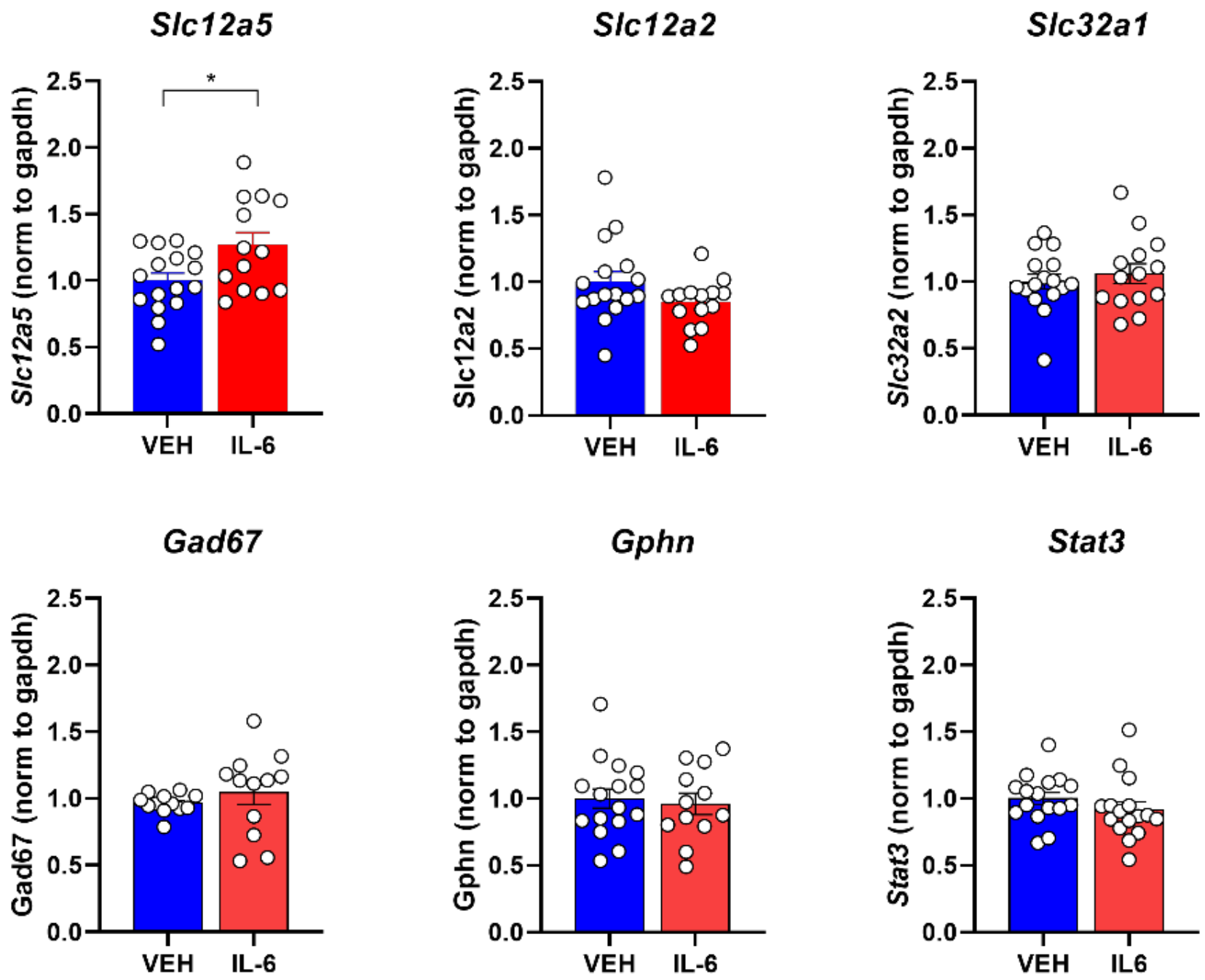


**Fig. R2.5.** *Quantification of mRNA relative expression of chloride transporters KCC2, Slc12a5, and NKCC1, Slc12a2, GABA-related encoding genes (Gad67, Slc32a1, Gphn) and Stat3 in the hippocampi of Vehicle and IL-6 exposed P10 mice. Data are presented as mean ± SEM of N=16 Vehicle vs N=13 IL-6 mice from al least three independent experiments. Unpaired t test, ns > p 0.05, * p < 0.05.*

**Reviewer #3 (Remarks to the Author):**

Desiato, Pozzi, and colleagues describe, in the manuscript titled "Elevated prenatal IL-6 levels disrupt early patterns of hippocampal electrical activity by targeting GABAergic signaling at perinatal stage", the mechanisms by which cytokine interleukin-6 (IL-6) influences early brain development. Using a variety of method including IL-6 injection to pregnant dams at gestation day 15.5 and IL-6 application to neuronal culture with those derived from postnatal days of embryos coupled with electrophysiology and histochemical analyses, the authors show that IL-6 influences GABAergic transmission, leading to the acceleration of the GABA developmental switch and reduction in neural activity, which culminates in abnormal maturation of offspring's hippocampal circuitry. This work added to a growing number of works highlighting the modulatory roles of maternally derived immune molecules in developing neuronal circuits. The strength of this manuscript lies in the elucidation at the molecular level of the effects of IL-6 on the premature developmental switch in GABAergic signaling. Through extensive analyses, the authors, for example, showed that IL-6 treatments lead to premature lowering of chloride ion in neurons and changed expression of various GABA-related genes.

Overall, this is an important work providing novel ideas to the neuroimmunology field in understanding how cytokines influence brain development. I am in support of the publication of this work in essence, but wanted to make the following comments.

**We sincerely appreciate the reviewer's positive comments on our work. Our responses are provided below.**

1. While the authors showed that STAT3 gene expression is increased in neuronal culture following IL-6 treatments, it is not clear whether the STAT3 level is changed in the hippocampal neurons of the affected offspring. In other words, while the authors alluded that IL-6 directly affects GABA-signaling in the brain, they haven't proved this hypothesis. For example, earlier reports have shown that IL-17 in the mother and CCL2 in the offspring may play roles downstream of IL-6. Is it possible that IL-6 mediates the effects through the modulation of other effector molecules, such as IL-17 and CCL2? While it is desirable to test the effects of these immune molecules in influencing GABA-related circuits, the authors at least need to clarify these points in the discussion.

**We thank the reviewer for this question. Concerning other possible molecules acting downstream of IL-6, we have already examined the expression of several immune molecules, as shown in Figure S1F of the manuscript. In particular, in the brain of P5 pups exposed to IL-6 we did not observe changes in the expression of cytokines or chemokine, including IL-6, TNFa, IFNg, IL1b, Ccl4, Ccl11, Cxcl10, Foxp3 and Nos. This finding further supports our previous evidence, [14], which showed no differences in the transcriptional level of mRNAs encoding inflammatory mediators in the adult brains of IL6-exposed offspring. In addition, as suggested by the reviewer, we tested the transcriptional levels of IL17Ra [31] and CCL2 [32] specifically in the hippocampi of pups exposed to IL-6 or vehicle, without seeing any changes in the two groups (Fig. R3.1). This data has been added in the revised version of the manuscript and the revised figure S1.**

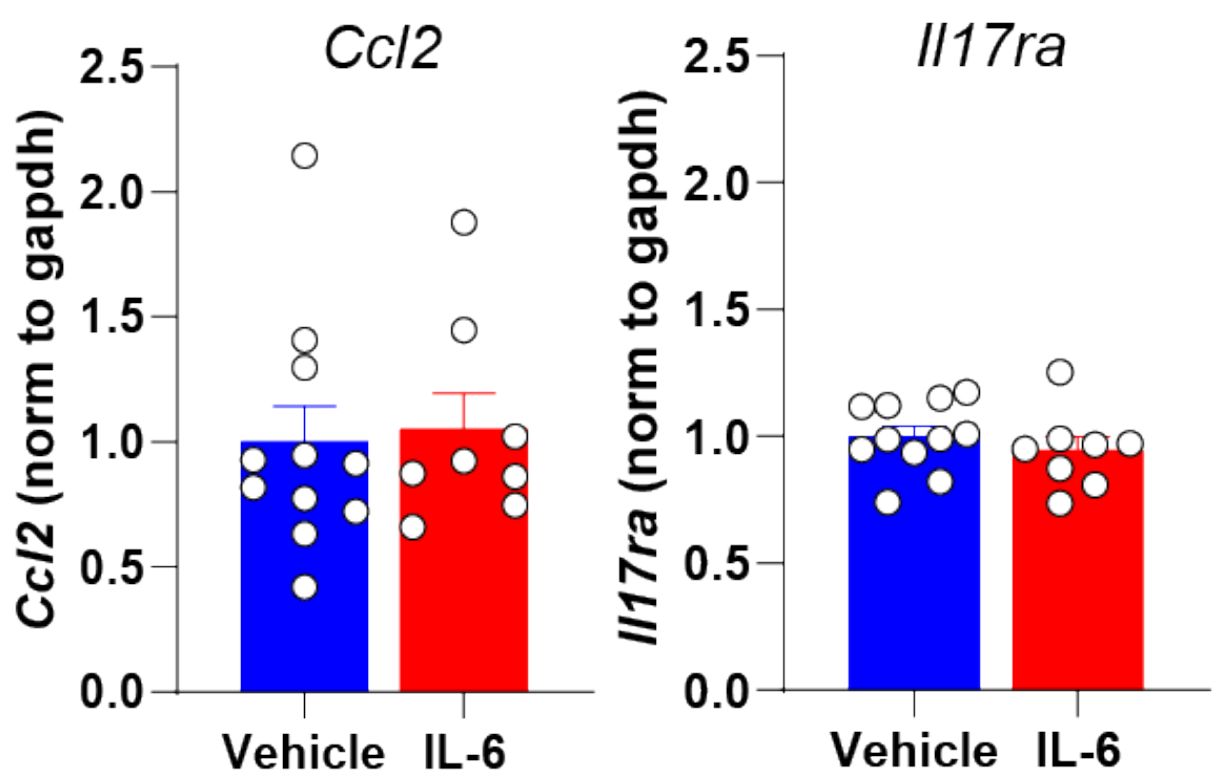


**Fig. R3.1.** *Quantification of mRNA relative expression of immune molecules C-C motif chemokine 2 (Ccl2), and interleukin 17 receptor a (Il17ra) in the hippocampi of Vehicle and IL-6 exposed P5 mice. Data are presented as mean ± SEM of N=11 Vehicle vs N=8 IL-6 mice from al least three independent experiments. Unpaired t test, ns > p 0.05.*

2. Also, what are the long-lasting effects of IL-6 treatments? Did the authors detect changes in the neural activity or GABA-related gene expression in the brains of MIA adult mice?

**We thank the reviewer for this comment. As already pointed out for reviewer#2, the long term consequences of IL-6 model have been extensively evaluated and discussed in our previous work [14]. We previously reported that adult offspring prenatally exposed to IL-6 shown a higher excitatory transmission whilst the GABAergic transmission was not changed. This excitatory/inhibitory unbalance was associated with altered hippocampal-related functional connectivity and behavior. In addition, we performed sociability test in adult mice prenatally exposed to IL-6 or vehicle (See Fig. 2.2) and we found that IL-6–treated mice spent significantly less time in direct object exploration (Fig. R2.2). These data may be explained by different hypotheses, including subtle alterations in sensory processing [28], increased avoidance of inanimate objects [29], or reduced object-directed exploration secondary to an enhanced motivational salience of the social stimulus as observed in mouse models of ASD [30].**

3, While the authors stated that USV phenotypes are not different in P5 control and IL-6 mice, many earlier studies by other groups have reported that maternal immune activation produced significant changes in USV calls. The authors need to clarify why they have seen different results. Is it because they have measured USV calls at earlier time points (P5), while many others did that at later P7-P9?

**We thank the reviewer for raising this important point. In line with the reviewer's suggestion, we repeated the USV measurements at a later developmental time point. Consistent with the maternal odor preference at P10 where IL-6 exposed mice displayed an impairment of this task, the number of USVs was significantly altered at this later stage and could be fully restored by KCC2 inhibition (See point 1 reviewer 2, figure R2.1). This new finding was included in the revised version of the manuscript. We thank the reviewer for this valuable suggestion, which has strengthened the robustness of our manuscript.**

**References:**


1. Bender, R.A., *et al.* Synchronized network activity in developing rat hippocampus involves regional hyperpolarization-activated cyclic nucleotide-gated (HCN) channel function. *The European journal of neuroscience* **22**, 2669-2674 (2005).
2. Menendez de la Prida, L., Bolea, S. & Sanchez-Andres, J.V. Origin of the synchronized network activity in the rabbit developing hippocampus. *The European journal of neuroscience* **10**, 899-906 (1998).
3. Buzsaki, G., Anastassiou, C.A. & Koch, C. The origin of extracellular fields and currents--EEG, ECoG, LFP and spikes. *Nature reviews. Neuroscience* **13**, 407-420 (2012).
4. Haider, B., Schulz, D.P., Hausser, M. & Carandini, M. Millisecond Coupling of Local Field Potentials to Synaptic Currents in the Awake Visual Cortex. *Neuron* **90**, 35-42 (2016).
5. Khazipov, R., Leinekugel, X., Khalilov, I., Gaiarsa, J.L. & Ben-Ari, Y. Synchronization of GABAergic interneuronal network in CA3 subfield of neonatal rat hippocampal slices. *The Journal of physiology* **498 ( Pt 3)**, 763-772 (1997).
6. Ben-Ari, Y., Cherubini, E., Corradetti, R. & Gaiarsa, J.L. Giant synaptic potentials in immature rat CA3 hippocampal neurones. *The Journal of physiology* **416**, 303-325 (1989).
7. Wester, J.C. & McBain, C.J. Interneurons Differentially Contribute to Spontaneous Network Activity in the Developing Hippocampus Dependent on Their Embryonic Lineage. *The Journal of neuroscience : the official journal of the Society for Neuroscience* **36**, 2646-2662 (2016).
8. Garaschuk, O., Hanse, E. & Konnerth, A. Developmental profile and synaptic origin of early network oscillations in the CA1 region of rat neonatal hippocampus. *The Journal of physiology* **507 ( Pt 1)**, 219-236 (1998).
9. Okuyama, T., Kitamura, T., Roy, D.S., Itohara, S. & Tonegawa, S. Ventral CA1 neurons store social memory. *Science* **353**, 1536-1541 (2016).
10. Gkogkas, C.G., *et al.* Autism-related deficits via dysregulated eIF4E-dependent translational control. *Nature* **493**, 371-377 (2013).
11. Ehninger, D., *et al.* Reversal of learning deficits in a Tsc2+/- mouse model of tuberous sclerosis. *Nature medicine* **14**, 843-848 (2008).
12. Patrich, E., Piontkewitz, Y., Peretz, A., Weiner, I. & Attali, B. Maternal immune activation produces neonatal excitability defects in offspring hippocampal neurons from pregnant rats treated with poly I:C. *Scientific reports* **6**, 19106 (2016).
13. Ito, H.T., Smith, S.E., Hsiao, E. & Patterson, P.H. Maternal immune activation alters nonspatial information processing in the hippocampus of the adult offspring. *Brain, behavior, and immunity* **24**, 930-941 (2010).
14. Mirabella, F., *et al.* Prenatal interleukin 6 elevation increases glutamatergic synapse density and disrupts hippocampal connectivity in offspring. *Immunity* **54**, 2611-2631 e2618 (2021).
15. Corradini, I., *et al.* Maternal Immune Activation Delays Excitatory-to-Inhibitory Gamma-Aminobutyric Acid Switch in Offspring. *Biological psychiatry* (2017).

16. Tan, M.S.Y., *et al.* A STAT3-based gene signature stratifies glioma patients for targeted therapy. *Nature communications* **10**, 3601 (2019).
17. Priego, N., *et al.* STAT3 labels a subpopulation of reactive astrocytes required for brain metastasis. *Nature medicine* **24**, 1024-1035 (2018).
18. Shirazi, R., *et al.* Glucagon-like peptide 1 receptor induced suppression of food intake, and body weight is mediated by central IL-1 and IL-6. *Proceedings of the National Academy of Sciences of the United States of America* **110**, 16199-16204 (2013).
19. Silva, V.R., *et al.* Hypothalamic S1P/S1PR1 axis controls energy homeostasis. *Nature communications* **5**, 4859 (2014).
20. Heubl, M., *et al.* GABAA receptor dependent synaptic inhibition rapidly tunes KCC2 activity via the Cl(-)-sensitive WNK1 kinase. *Nature communications* **8**, 1776 (2017).
21. Ganguly, K., Schinder, A.F., Wong, S.T. & Poo, M. GABA itself promotes the developmental switch of neuronal GABAergic responses from excitation to inhibition. *Cell* **105**, 521-532 (2001).
22. Suresh, J., *et al.* Network burst activity in hippocampal neuronal cultures: the role of synaptic and intrinsic currents. *Journal of neurophysiology* **115**, 3073-3089 (2016).
23. Kobayashi, T., *et al.* Revealing single-neuron and network-activity interaction by combining high-density microelectrode array and optogenetics. *Nature communications* **15**, 9547 (2024).
24. Pasquale, V., Martinoia, S. & Chiappalone, M. Stimulation triggers endogenous activity patterns in cultured cortical networks. *Scientific reports* **7**, 9080 (2017).
25. Wagenaar, D.A., Pine, J. & Potter, S.M. An extremely rich repertoire of bursting patterns during the development of cortical cultures. *BMC neuroscience* **7**, 11 (2006).
26. Teppola, H., Acimovic, J. & Linne, M.L. Unique Features of Network Bursts Emerge From the Complex Interplay of Excitatory and Inhibitory Receptors in Rat Neocortical Networks. *Frontiers in cellular neuroscience* **13**, 377 (2019).
27. Takeda, K., *et al.* Targeted disruption of the mouse Stat3 gene leads to early embryonic lethality. *Proceedings of the National Academy of Sciences of the United States of America* **94**, 3801-3804 (1997).
28. Velloso, F.J., Zaritsky, R., Houbeika, R.Y., Rios, N. & Levison, S.W. Interleukin-6 produces behavioral deficits in pre-pubescent mice independent of neuroinflammation. *Brain, behavior, and immunity* **126**, 275-288 (2025).
29. Arakawa, H. Revisiting sociability: Factors facilitating approach and avoidance during the three-chamber test. *Physiology & behavior* **272**, 114373 (2023).
30. Stoppel, D.C. & Anderson, M.P. Hypersociability in the Angelman syndrome mouse model. *Experimental neurology* **293**, 137-143 (2017).
31. Choi, G.B., *et al.* The maternal interleukin-17a pathway in mice promotes autism-like phenotypes in offspring. *Science* **351**, 933-939 (2016).
32. Cui, J., *et al.* Inflammation of the Embryonic Choroid Plexus Barrier following Maternal Immune Activation. *Developmental cell* (2020).

**REVIEWER COMMENTS**

**Reviewer #1 (Remarks to the Author):**

While the authors have now addressed most of the concerns raised, several issues related to MEA recordings still need clarification.

1. The methods lack details on how the reported MFPR values are obtained (how many electrodes/area were analysed per recording, were the frequency counts from all electrodes averaged etc).

**We thank the reviewer for this comment and apologize if this aspect was still not completely clear. In the "methods" section we described in detail how MFPR values were obtained as follows*:***

**"*Recordings and LFP detection were performed using BrainWave 5 software from 3Brain and referred to as Mean Field Potential Rates (MFPR) values. LFP were identified using the Hard Threshold algorithm, (High Threshold=200 µV, Low Treshold= -200 µV, Energy Window=40ms, Max Wave Duration=80ms, refractory period=5ms).***

**If we understand correctly, the reviewer's concern is that silent electrodes may have been included in the analysis, thus potentially affecting the results. However, in the methods we also reported that: "*Only electrodes with a MFPR greater than 0.8 Field Potential (Fp)/minute have been considered for the analysis."* Therefore, inactive (silent) electrodes were excluded and did not influence the results.**
**Regarding "how many electrodes/area were analyzed per recording", this information was already provided in the Figure S1A of the revised manuscript, which shows the number of active electrodes for each experiment. This parameter was consistent across recordings.**
**Concerning the statement "were the frequency counts from all electrodes averaged", the values shown in the graphs represent the average firing frequency across all active electrodes within each recording. To improve clarity, we are willing to explicitly include this statement in the Methods section.**

2. The authors indicate in the methods that 'At the end of each recording session, a high-definition (HD) image of the slice was taken using HD digital microscope (Bysameyee) and subsequently superimposed to the activity map ensuring a precise electrical-anatomical correlation'. Thus, it should be possible to find in which hippocampal subfield the electrodes were located. This is important, because CA3 typically is more active compared to CA1, and the localization of the electrodes selected for analysis can make a difference.

**We thank the reviewer again for raising this point and apologize for the lack of clarity in our previous comment. The image acquired at the end of the recording sessions was obtained using a camera that enables the acquisition of a bright-field image of the recorded slice with the sole purpose of identifying the hippocampus among other anatomical regions without performing a precise mapping of its subfields. In addition, the bright-field images we acquired with this device did not allow us to precisely identify the hippocampal subfields, as shown in the representative images below.**

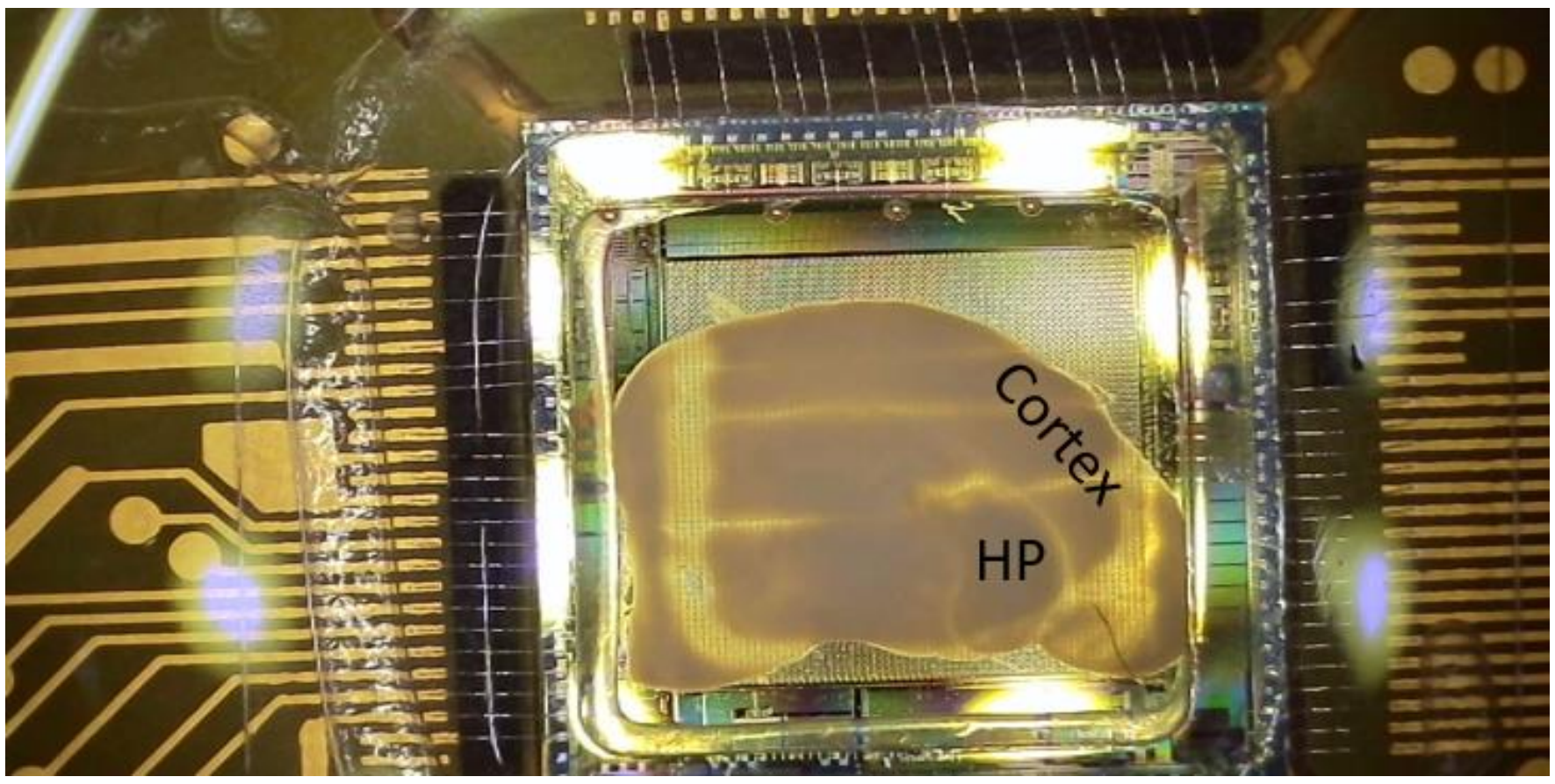


**For this purpose, a retrospective immunofluorescence analysis of the recorded slice using cell-specific markers immediately after the recording session would represent a more rigorous approach to accurately identify the cellular composition of the distinct anatomical regions. As we recognize that this represents a limitation of the technological approach used, we have explicitly acknowledged it in the Methods section. However, we agree with the reviewer that this is an important point, and for that reason, we would like to further investigate this specific aspect in a dedicated follow-up study. We therefore greatly appreciate the reviewer's insightful suggestion, which will help guide and improve our work in this direction.**

3. The manuscript remains confusing in terms of terminology related to network activity. The authors conclude in the results '…IL-6 exposure reduces hippocampal spiking activity at P5, without affecting the synchrony of the network activity.' However, throughout the text they refer to 'altered hippocampal network activity', or in some cases 'spiking activity'; the terminology should be consistent throughout.

**The reviewer is correct, and we apologize for the lack of clarity in the terminology used throughout the manuscript. We have thoroughly revised the text, replacing terms such as "spiking activity" and "network activity" with more appropriate terminology to avoid ambiguity and more accurately reflect the data presented. In particular, we now use the terms "LFP occurrence rate" (or LFP event frequency) and network synchrony to dissect the changes observed at P5. In contrast, at P3, where both MFPR and SNE frequency are increased, we refer to this as an enhancement of "hippocampal network activity," as both parameters are affected. We sincerely appreciate the reviewer's comment since we believe that after this revision the manuscript improved for clarity and accurateness, better reflecting the data presented.**

4. In the revised text, the authors now refer to the intensity and duration of the LFP discharge as a measure of the strength of synaptic signaling, but there is no data on LFP amplitude or duration in the manuscript. Was this parameter affected by the treatments?

**We apologize for this lack of clarity. The "amplitude" of the LFPs was analyzed but not reported in the manuscript; however, as shown in the figure below, this parameter was not altered under any condition. This data will be included in the revised version of the manuscript as supplementary figure 1C.**

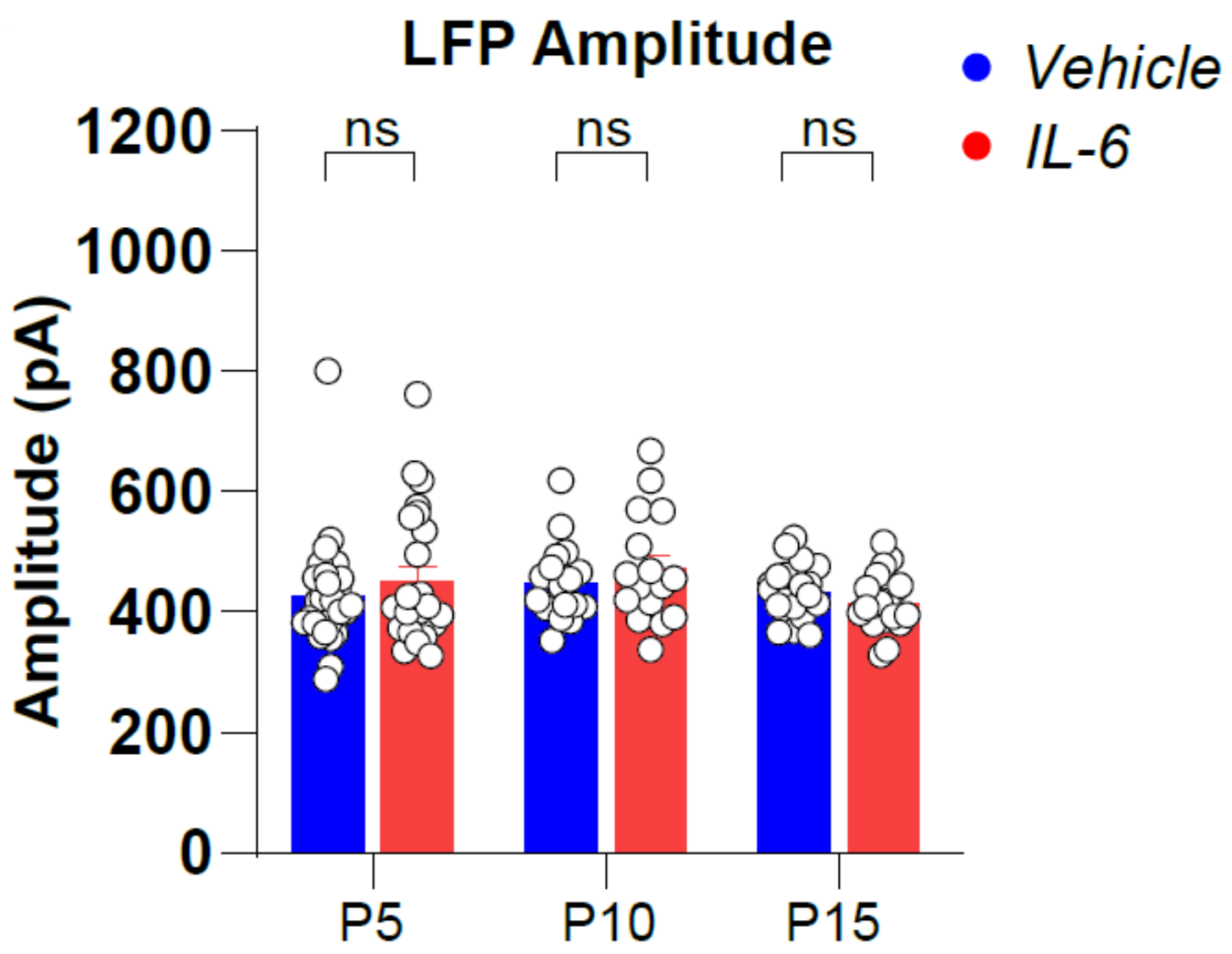


**Regarding the term "duration" we did not refer to the duration of individual LFP events, which was not measured, but rather to the duration of LFP bursts, a parameter that is directly related to LFP frequency (MFPR). However in light of the reviewer's comment, we recognize that the sentence "…the intensity and duration of the associated discharge (the MFPR)…" could be potentially misleading and it will therefore be replaced with the following sentence (full sentence provided with changes in *italic*): "While the occurrence of SNEs *may* reflect the intrinsic tendency of specific leader neurons to spontaneously fire [82-84], *the amplitude and frequency (MFPR) of LFPs may reflect* the strength of synaptic excitatory signaling as previously reported [82 85 86] ". We believe that this revised sentence is clearer and provides a more cautions and accurate description of the phenomenon. We thank the reviewer again for raising this point, which has helped us improve the clarity and accuracy of the manuscript.**

5. Minor: Original comment #4 required the condition (baseline vs drug) to be indicated in the raster plots of the figure 3, the labeling of the insets was ok already in the original version.

**We apologize for misinterpreting the reviewer's original request. The correct condition has now been clearly indicated in the raster plots in Figure 3 and 5.**

6. Minor: The quality of the traces in the figure S4 should be checked (this could be an issue with the PDF conversion)

**We have checked the quality of the traces in Figure S4, which have now been improved.**

**Reviewer #2 (Remarks to the Author):**

The authors have substantially strengthened the manuscript and addressed most of my major concerns through meaningful new experiments and clearer mechanistic framing. Most importantly, they now provide a much-improved developmental sequence showing that enhanced GABAergic transmission is already present at P3, preceding KCC2 upregulation at P5 and later behavioral abnormalities at P10. This temporal refinement significantly improves the central mechanistic argument. The addition of new in vivo experiments, particularly the P10 behavioral analyses (MOP and USV) together with pharmacological rescue by KCC2 inhibition, represents a substantial improvement over the original submission and provides stronger functional relevance. The new picrotoxin intervention further supports the proposed causal relationship between early GABAergic activity and later KCC2 dysregulation in vivo. These additions move the work beyond its previous heavy dependence on ex vivo systems.

That said, some limitations remain. My original request for more definitive in vivo mechanistic precision – particularly direct cell-type-specific validation of STAT3/GABAergic neuron involvement – has only been partially addressed. While the authors provide reasonable technical explanations for why conditional or selective in vivo approaches were not feasible, this remains an important unresolved limitation. Similarly, long-term circuit and behavioral consequences are better contextualized, but much of this still relies on prior published work rather than direct incorporation into the current manuscript.

Overall, however, the revision is substantially improved, more mechanistically coherent, and considerably stronger than the initial submission. Although not every point has been fully resolved at the highest mechanistic level, the authors have addressed the core concerns sufficiently for publication, provided that remaining limitations are clearly acknowledged in the discussion.

I therefore recommend acceptance after minor revision. Specifically, the authors should explicitly temper their claims regarding direct in vivo cellular specificity and clearly acknowledge that STAT3/GABAergic neuron-selective causality remains to be demonstrated. They should also ensure that the discussion more clearly distinguishes between findings directly established in the current manuscript and conclusions inferred from prior published work, particularly regarding long-term outcomes. Finally, a brief clarification of these remaining mechanistic limitations would improve interpretive balance without requiring substantial additional experimentation.

Reviewer 1's technical concerns regarding electrophysiology, chloride measurements, and developmental timing appear to have been addressed thoroughly with substantial new controls and experiments. Reviewer 3's broader questions regarding downstream immune mediators and behavioral interpretation were also addressed reasonably well, though – as with my comments – some mechanistic specificity remains inferential rather than definitive.

**We sincerely thank the reviewer for the positive assessment of our revisions and for recognizing the value of the improved developmental sequence described, in vivo behavioral assays, and pharmacological interventions. We are very grateful for the constructive feedback throughout this review process and for the recommendation for acceptance. Concerning the reviewer's insight regarding the remaining limitations, we agree that achieving cell-type-specific precision and tracking long-term consequences are important objectives for future studies.**

**Specifically, regarding the STAT3/GABAergic specificity, a direct in vivo cell-type-specific validation represents the necessary next step to fully demonstrate this mechanism. We have now explicitly acknowledged this limitation to our discussion (Sentence provided in bold in the first page of discussion): "It should be noticed that although our ex vivo and in vitro data strongly suggest a STAT3-dependent enhancement of GABAergic activity triggered by IL-6, a direct in vivo evidence of this specific mechanism remains to be fully demonstrated. Further studies employing inducible, cell-type-specific conditional knockout models will be necessary to causally define the in vivo role of STAT3 in GABAergic neurons within this developmental window."**

**Regarding the specific long-term neuronal circuit modifications and behavioral consequences, we agree that relying on prior literature to bridge these long-term effects constitutes an indirect inference rather than a direct relationship. For that reason, we have edited the Discussion section to clearly distinguish between our direct experimental findings and the conclusions we infer based on our previous study (Sentence provided in bold in the last page of discussion): "However, while the present study demonstrates the functional and behavioral consequences of maternal IL-6 elevation in offspring up to P10, the specific and direct long-term behavioral alterations in fully mature offspring remain to be thoroughly evaluated with dedicated longitudinal studies."**

**By openly acknowledging these points in the manuscript, we believe the final text offers a more cautions and objective presentation of our findings.**

**Reviewer #3 (Remarks to the Author):**

The authors addressed all the critiques I raised. I no longer have any comments.

**We sincerely thank the reviewer for the time, constructive feedbacks, and thoughtful insights throughout the review process. Her/him comments and suggestions have significantly strengthened the quality and clarity of our manuscript.**
